# Transition From Wild to Domesticated Pearl Millet (*Pennisetum glaucum)* Revealed in Ceramic Temper at Three Middle Holocene Sites in Northern Mali

**DOI:** 10.1007/s10437-021-09428-8

**Published:** 2021-03-16

**Authors:** Dorian Q. Fuller, Aleese Barron, Louis Champion, Christian Dupuy, Dominique Commelin, Michel Raimbault, Tim Denham

**Affiliations:** 1 Institute of Archaeology, University College London, 31–34 Gordon Square, London, WC1H 0PY UK; 2 School of Cultural Heritage, Northwest University, Xi’an, China; 3 Department of Archaeology, Max Planck Institute for the Science of Human History, Jena, Germany; 4 School of Archaeology and Anthropology, Australian National University, Banks Building, Canberra, ACT 2601 Australia; 5 Institute of Archaeological Sciences, Goethe University, Norbert-Wollheim-Platz 1, 60629 Frankfurt am Main, Germany; 6 Laboratoire Archéologie et Peuplement de l’Afrique (APA), Anthropology Unit of the Department of Genetics and Evolution (GenEv), University of Geneva, Geneva, Switzerland; 7 Institut des Mondes Africains (IMAF, UMR 8171, CNRS), Paris, France; 8 Laboratoire Méditerranéen de Préhistoire Europe Afrique (LAMPEA, UMR 7269 CNRS, Aix-en-Provence, France

**Keywords:** Plant domestication, Neolithic, Later Stone Age, Archaeobotany, MicroCT, *Cenchrus americanus*

## Abstract

Imprints of domesticated pearl millet (*Pennisetum glaucum* (L.) R. Br.) spikelets, observed as temper in ceramics dating to the third millennium BC, provide the earliest evidence for the cultivation and domestication process of this crop in northern Mali. Additional sherds from the same region dating to the fifth and fourth millennium BC were examined and found to have pearl millet chaff with wild morphologies. In addition to studying sherds by stereomicroscopy and subjecting surface casts to scanning electron microscopy (SEM), we also deployed X-ray microcomputed tomography (microCT) on eleven sherds. This significantly augmented the total dataset of archaeological pearl millet chaff remains from which to document the use of the wild pearl millet as ceramic temper and the evolution of its morphology over time. Grain sizes were also estimated from spikelets preserved in the ceramics. Altogether, we are now able to chart the evolution of domesticated pearl millet in western Africa using three characteristics: the evolution of nonshattering stalked involucres; the appearance of multiple spikelet involucres, usually paired spikelets; and the increase in grain size. By the fourth millennium BC, average grain breadth had increased by 28%, although spikelet features otherwise resemble the wild type. In the third millennium BC, the average width of seeds is 38% greater than that of wild seeds, while other qualitative features of domestication are indicated by the presence of paired spikelets and the appearance of nondehiscent, stalked involucres. Nonshattering spikelets had probably become fixed by around 2000 BC, while increases in average grain size continued into the second millennium BC. These data now provide a robust sequence for the morphological evolution of domesticated pearl millet, the first indigenous crop domesticated in western Africa.

**Supplementary Information:**

The online version contains supplementary material available at 10.1007/s10437-021-09428-8.

## Résumé

Des empreintes d’épillets de mil domestiqué (*Pennisetum glaucum* (L.) R. Br.) observées dans des céramiques datées du 3^e^ millénaire av. J.-C. provenant du nord du Mali constituent les plus anciens témoins de la mise en culture et de la domestication de cette céréale. Des tessons supplémentaires issus de la même région se rapportant aux 5^e^ et 4^e^ millénaires av. J.-C. ont été examinés et ont révélé des empreintes de balle de mil de morphologies sauvages. En plus de l’observation de leurs surfaces par stéréo-microscopie, et de l’observation des moulages d’empreintes au microscope à balayage, onze d’entre eux ont fait l’objet des micro-tomographies aux rayons X (microCT). Ces examens enrichissent considérablement l’ensemble des données archéologiques concernant l’utilisation du mil sauvage comme dégraissant végétal et son évolution morphologique à travers le temps. La taille des grains a aussi été estimée à partir des épillets conservés dans la céramique. En tenant compte des données enregistrées lors d’études antérieures, nous pouvons désormais retracer l’évolution du mil domestiqué en Afrique de l’Ouest à travers trois caractéristiques : l’évolution des involucres pédonculés à égrenage non-spontané; l’apparition d’involucres multiples par épillets, des épillets appariés le plus souvent; l’augmentation de la taille des grains au vu de leur largeur. Déjà au 4^e^ millénaire avant J.-C., la largeur moyenne des grains a augmenté de 28% bien que les caractéristiques de l'épillet ressemblent au type sauvage. Au 3^e^ millénaire avant J.-C., elle est 38% supérieure à celle du morphotype sauvage, tandis que des caractéristiques qualitatives de la domestication sont avérées par la présence d’épillets appariés et par celle d’involucres pédonculés à égrenage non-spontané. La non-déhiscence des épillets est un caractéristique de la domesticité qui s’est probablement fixé vers 2000 avant J.-C., tandis que l’augmentation de la taille moyenne des grains s’est poursuivie tout au long du 2^e^ millénaire av. J.-C. Ces données fournissent désormais une séquence robuste concernant l’évolution morphologique du mil, la première céréale indigène domestiquée en Afrique de l’Ouest.

## Introduction

**Fig. 1:**
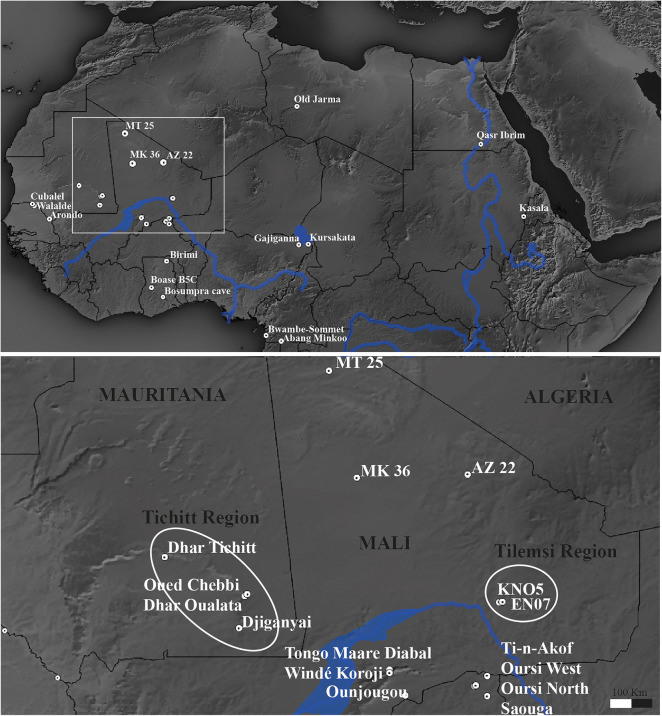
Map of sites with important archaeological pearl millet evidence, including metrical or morphological domestication evidence. Lower map indicates area of detail, including sites with new evidence reported in this article (MT25, MK36, and AZ22)

Pearl millet (*Pennisetum glaucum* (L.) R. Br., [syn. *Cenchrus americanus* (L.) Morrone]), is one of the most consumed staple crops of sub-Saharan Africa and tropical India (Brunken et al. 1977; Stevens and Fuller 2018). West African agricultural developments began with pearl millet domestication, although the documentation of its domestication process and predomestication exploitation is limited. Once domesticated, pearl millet was integrated within the pastoral subsistence system, providing a potent economic package ready for dispersal. The oldest evidence for this situation is documented in the Lower Tilemsi valley (Mali), where pastoralism and pearl millet cultivation were integrated alongside fishing, hunting, and collecting from 2500 BC (Fig. 1; Manning and Fuller 2014; Manning et al. 2011). A subsequent rapid spread eastward across the Sahara has been postulated based on evidence for domesticated forms reaching eastern Sudan by approximately 1850 BC (Beldados et al. 2018; Winchell et al. 2018) and India, via maritime links, by ca. 1700 BC (Boivin and Fuller 2009; Pokharia et al. 2014).

The wild progenitor of the cultivated pearl millet species has been identified as *Pennisetum violaceum* (Lam.) Rich. [syn. *P. americanum* subsp. *monodii* (Maire) Brunken] (Brunken 1977; Brunken et al. 1977; D'Andrea and Casey 2002). The natural distribution of this species is restricted to the Sahelian zone, from Senegal to northern Sudan (Brunken 1977; Harlan 1992; Upadyaya et al. 2017). However, it is often inferred that domestication occurred in the western part of this range, between Niger and Mauritania (Clotault et al. 2012; Dupuy 2014; Fuller 2003; Fuller and Hildebrand 2013; Tostain 1998). The modeling of modern genomic data fits with the hypothesis of a southwestern Saharan origin for the domesticated form, whence pearl millet spread westward to Mauritania and southward into the savanna south of the Niger River bend (Burgarella et al. 2018).

Here, we report new evidence for wild pearl millet, dating back to the middle Holocene (~5000 BC), from northeast Mali within the western Saharan zone, and the subsequent appearance of domesticated traits by mid-third millennium BC. These data derive from the conventional study of impressions on sherd surfaces (see Fuller et al. 2007) and microCT-scanning of sherds’ interior content (following the recently developed methods by Barron and Denham 2018; Barron et al. 2017, 2020a, 2020b). We then combine these data with the available long-term archaeological evidence in western Africa to identify the evolutionary trends of pearl millet’s domestication and diversification.

## Defining and Documenting Domestication in Pearl Millet

**Fig. 2:**
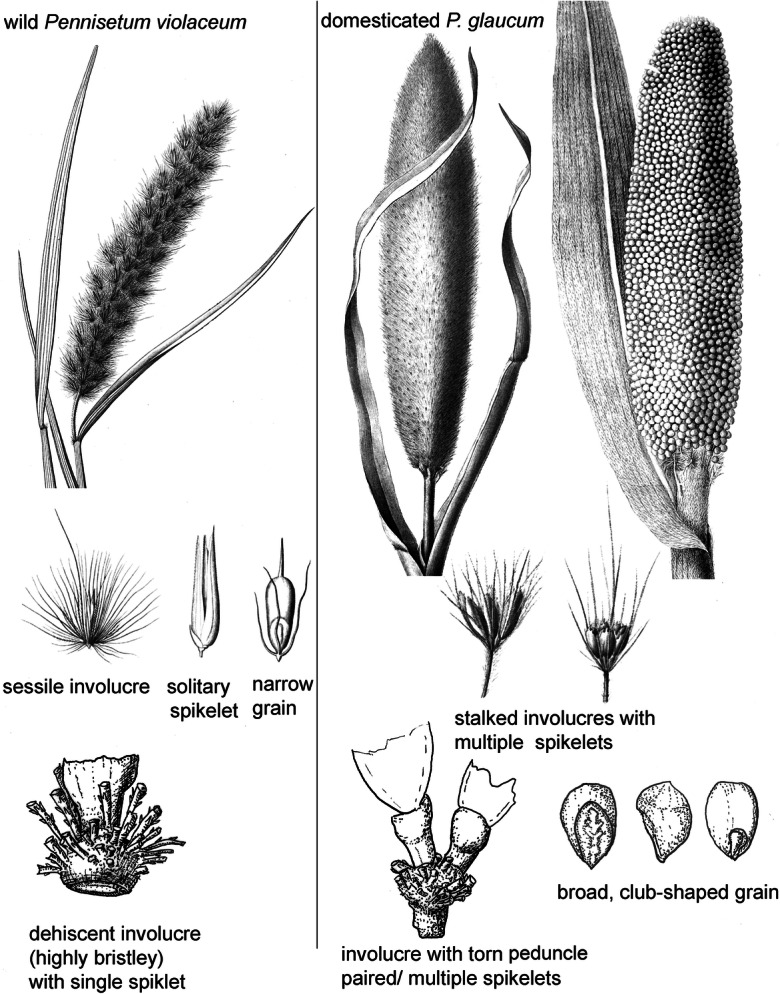
The morphology of pearl millet, comparing wild (*Pennisetum violaceum*) and domesticated (*P. glaucum*) forms. Spike and involucre of *P. violaceum* after Kunth (1835); *P. glaucum*, middle, and stalked involucres after Engler (1895); *P. glaucum* spike, right, after Reale Accademia di Scienze, Lettere ed Arti (1786). Chaff and grain drawings in lower row by DQ Fuller

Pearl millet shares many of the same morphological changes known for other cereal domestications (Fuller 2007; Fuller and Allaby 2009; Harlan et al. 1973). In the case of pearl millet, some of these key traits are illustrated in Fig. 2. Some traits, such as the increase in spike length and the increase in grains per harvested head, are not detectable archaeologically. Also difficult to document is the general reduction in the number and length of bristles, which tend to be longer and more prominent in the wild races and more “primitive” in cultivated races. Bristles are reduced, however, alongside increasing grain plumpness in more advanced landraces. Among cultivars, there is a spectrum of variation in how tightly clasped grains are within the husk. The extent to which grains extend beyond the husk is indicative of free-threshing forms. In contrast, the longer-clasping chaff, typical of wild *Pennisetum violaceum* and some of the more bristly (“primitive”) cultivars, requires distinct dehusking processes after threshing. Thus, postdomestication evolution includes a reduction in husk length and bristle length, which correlates to pearl millet spikes that are free-threshing and requiring less labor for processing. Although racial subclassifications of pearl millet have been proposed (Brunken et al. 1977), the full spectrum from hulled to free-threshing forms is included in the widespread, basic *Pennisetum* typhoides race.

Two traits are readily documented archaeologically in cereals. The first, often regarded as the *sine qua non* of cereal domestication, is the evolution of nonshattering spikes, making seed dispersal reliant on human harvesting, threshing, and sowing. In *Pennisetum*, wild dispersal is through shattering (abscission) of the involucre, including bristles and spikelet. In domesticated forms, the involucre has developed a nondehiscent stalk (peduncle) below the bristles; the peduncle is torn due to threshing, and this characterizes domestication (see Fig. 2; Brunken et al. 1977; Dupuy 2017; Manning et al. 2011; Poncet et al. 2000). The second trait is an increase in grain size (grain volume), which occurred through increases in breadth and thickness. In pearl millet, grain breadth and thickness are strongly correlated (usually 1:1), so documenting just one of these dimensions is sufficient for comparing grain size. The enlarged seeds of domesticated pearl millet become markedly obovate or “club-shaped” (D'Andrea et al. 2001; Neumann et al. 1996; Zach and Klee 2003). Grain size increase can be documented through measurements on preserved grains or measurable spikelets in ceramic impressions. Previous studies suggested that a significant increase in *Pennisetum* grain size was delayed until after primary domestication (in terms of seed dispersal) and that there may have been several regional processes for the selection of grain enlargement (Fuller 2007; Manning et al. 2011). However, this inference may be due, in part, to the lack of evidence for most of the domestication processes that took place before 2500 BC. New data reported below allows us to reassess these processes with evidence between 5000 BC and 2000 BC. Another change with domestication is the tendency of floret duplication, such that domesticated pearl millet involucres typically have two spikelets, and sometimes three to nine spikelets (Godbole 1928), as opposed to the typically single-grain, single spikelet involucre of wild *P. violaceum* (Brunken 1977; Fuller et al. 2007). All these three traits of domestication—nonshattering, grain size, and paired spikelets—are examined in this article.

## Materials and Methods

The sites AZ22, MT25, and MK36, were found in 1980 and 1982 by Centre National de la Recherche Scientifique (CNRS) missions, directed by Nicole Petit-Maire (Petit-Maire 1991; Petit-Maire and Riser 1983). During these missions, thousands of sherds were collected from dozens of sites. Currently, those sherds are part of the LAMPEA collection in Aix-en-Provence. Dominique Commelin (1984) and Michel Raimbault (1994) carried out the analysis of this ceramic collection.

The AZ22 site is in Oued Oukechert, near the Erg Ine Sakhane. Hearth charcoals gave a single conventional radiocarbon date of 5500–4950 cal BC (Gif-5228 6340±130 BP; Commelin et al. 1993). Currently, we cannot confirm that the sherds are contemporaneous with the hearth charcoals. However, due to the presence of lithic artifacts, including scrapers and denticulated blades, we infer a middle Holocene origin for the assemblage (Raimbault 1994, p. 814-816). The MT25 site is located near the Holocene lake, Oum el Assel, in the far northwestern region of Mali, named Erg Chech. This settlement comprises mainly scrapers and notched flakes (Raimbault 1994, p. 338-352). The organic material from a potsherd allowed a direct date of 4240–3090 cal BC (Pa-1023 4890±230 BP; Commelin et al. 1993). The site MK36 is located south of the two previous sites, more precisely on the southern border of the Erg Jmeya (Raimbault 1994, p. 542-548). Organic vegetal chaff in a spherical pottery fragment (decorated with dotted flames) was radiocarbon dated to 3020–1940 cal BC (Pa-1065 3795±200 BP; Commelin et al. 1993). As Manning et al. (2011) later noted, a fraction of the organic contents used for the AMS dating may contain old carbon and thus the radiocarbon ages may be some centuries older than the actual age of the ceramic. Nevertheless, MK36 is not older than 3020 BC and is perhaps closer to the middle or the end of the third millennium BC.

These sherds were reexamined for vegetable temper impressions because casts have proven useful for identifying remains of pearl millet elsewhere in West Africa (e.g., Fuller et al. 2007; Klee et al. 2000; Manning et al. 2011), and sorghum domestication in eastern Sudan (Barron et al. 2020b; Winchell et al. 2017, 2018). However, not all vegetable-tempered pottery produces identifiable food plant evidence (Fuller 2013; McClatchie and Fuller 2014). A total of 193 sherds collected from the surface of AZ22, 212 sherds from the surface of MT25, and 1601 sherds from the surface of MK36 were examined under a stereomicroscope at ×5–×20 magnifications by Louis Champion. After analysis, twelve sherds from AZ22, two sherds from MT25, and ten sherds from MK36 were selected for further study and were brought to London to cast surface impressions and for scanning electron microscopy (SEM).

Casting and SEM examination began with the sherds from AZ22. Casts of the grain/chaff impressions were made using a vinyl polysiloxane dental molding agent, which was applied to the surface of the potsherds. To have a clean and detailed cast, each sherd was cast three times. These casts were reexamined under the same stereomicroscope, and promising casts were selected for detailed examination by SEM. Recently, a new, nondestructive technique for visualizing the casts of organic temper within sherds, using high-resolution X-ray tomography acquired from microCT-scanning, has been pioneered at Australian National University (Barron et al. 2017; Barron and Denham 2018). Hence, we decided to explore one sherd from AZ22, using this method to assess the potential increase in data per sherd. The approach can produce a much larger archaeobotanical assemblage from each sherd and provide a more reliable documentation of morphological features in three dimensions (Barron et al. 2020a, 2020b). A study on Sudanese Neolithic sherds tempered with sorghum recovered 12 times more plant remains through microCT than through conventional surface casts. This greatly reduced the proportion of indeterminate remains regarding domesticated or wild status (Barron et al. 2020b). The promise of this approach also led us to examine all ten MK36 sherds using the microCT scanning method.

Sherds were scanned at the National Laboratory for X-ray Micro Computed Tomography (CT Lab) at the Australian National University. The AZ22 sherd was mounted in an aluminum tube (55-mm diameter) and stabilized with packing foam. It was scanned using a Heliscan high-resolution microCT system at 100 kV and a current of 65 mA for 11 h with a 3 mm aluminum filter, resulting in a voxel size of 22 μm (Latham et al. 2008; Myers et al. 2011). The MK36 sherds were divided into two groups, based on sherd size, and mounted in aluminum tubes with diameters of 20 mm and 40 mm, and stabilized with packing foam. These were then scanned using the same Heliscan high-resolution microCT system with a 3-mm aluminum filter at the respective energies of 80 kV and 100 kV, currents of 60 mA and 65 mA, and durations of 10 and 14 h. These resulted in voxel sizes of 7.67 μm and 15.59 μm, respectively. The resultant datasets were rendered using the open-source visualization software Drishti (v. 2.6.5) and Drishti Paint (v. 2.6.5) (Limaye 2012). The distribution of organic inclusions of lower density than the surrounding clay matrix was initially visualized in low resolution (1/64th) before individual inclusions were identified, segmented, and visualized at high resolution (see Barron and Denham 2018).

The maximum breadth of intact spikelets was measured for the impressions and microCT-scan visualizations. Spikelet maximum breadth is a close approximation of maximum grain breadth. As grain thickness and breadth are strongly correlated (Fuller 2007; Zach and Klee 2003), it is sufficient to use breadth to infer grain size differences between populations and change through time. For comparison, data from the literature was gathered to expand our datasets (e.g., Manning et al. 2011). In order to compare impression data with charred grain metrics (which are affected by shrinkage), impression metrics were reduced by 10% as a correction for the plausible effects of charring (see Fuller 2007). Assemblage means are plotted against a median estimate of site age (following the protocol of Fuller et al. 2012, 2014). When not available from primary sources, we estimated standard deviations based on sample size, following the factors in Pearson and Hartley (1976).

## Results

### AZ22

**Fig. 3:**
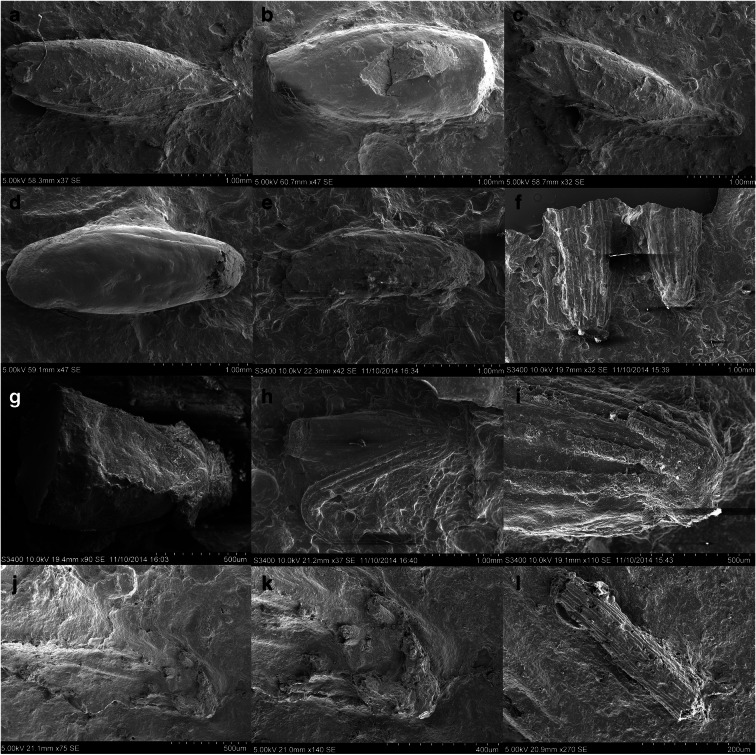
Example of surface chaff impressions from AZ22 studied by SEM: **a**–**e** single-grained spikelets (**a**–**c** AZ sherd 11; **d**–**e** AZ22 sherd 123); **f** involucres with bristle bases, indeterminate type left, and sessile right (AZ22 sherd 11); **g** solitary spikelet and bristles of involucre on indeterminate type (AZ22 sherd 27); **h**–**i** solitary spikelet in sessile involucre (AZ22 sherd 1); **j**–**k** solitary spikelet and bristles of involucre on indeterminate type (AZ22 sherd 12/13); **l** bristle fragment (AZ22 sherd 12/13)

Out of twelve sherds selected for casting from AZ22, ten produced plant impressions on the surface that were to some degree identifiable (Tables 1 and 2). These included remains that appear to be *Panicum* sp. (*n*=1) (Fig. 3b), an unidentified small grass grain (*n*=1), and, of particular significance, predominant *Pennisetum* remains, including several bristles and eleven grains in the husk (Fig. 3a), as well as lemma/palea fragments. However, none of these is diagnostic of wild or domesticated status. One sherd, however, preserved a complete *Pennisetum* involucre, including chaff and bristles and a sessile base (Fig. 3d) indicative of a shattering wild-type panicle (Fig. 3e). This appears to be a single-grained involucre, which is also typical of wild *Pennisetum* (Brunken et al. 1977; Marchais 1994), as opposed to the domesticated crop in which the majority (~80%) of involucres have two or more grains (Fuller et al. 2007; Godbole 1928; Manning and Fuller 2014). Therefore, the overall assemblage of surface impressions suggests exploitation of *Pennisetum* and the use of processed waste as ceramic temper. However, only a single specimen from the sherd surfaces could be determined as wild or domesticated status*.*

**Table 1:** Observations of impressions identified from AZ22, MT25, and MK36 sherds, including observation from surface impressions (SEM) and microCT (CT). For images from CTscans see OSM Images

Site name	Sherd no.	Method	Involucre w/ torn peduncle	Involucre w/ smooth base	Involucre base w/bristles (indet)	Bristle(s)	Paired spikelets	Spikelet	Lemma/palea frag.	Spikelet: *Echinochloa* (wild) in husk	Spikelet: cf. *Panicum* sp.	Spikelet *Sorghum* sp. (wild)	Other poaceae	Notes
AZ22	1	SEM		1										
AZ22	3	SEM												Culm node
AZ22	11	SEM		1	1			4						
AZ22	27	SEM		2	1	15		1						
AZ22	27	ct		30		100s		15						All with smooth involucre with single spikelet
AZ22	117	SEM				10								
AZ22	123	SEM						5						
AZ22	184	SEM									1			
AZ22	187	SEM						1						
AZ22	192	SEM												1 indeterminate grain
AZ22	12/13	SEM			1	2		7	2					Possible wild type involucre(?), with solitary spikelet
MT25	140	SEM				2		1						
MT25	175	SEM				2						1		Sorghum spikelet 2.4 mm wide
MK36	276	ct	2	1	1	3	2							Ripped involucre w/ paired spikelet in same impression; smooth abscission scar involucre w/ paired spikelet in same impression
MK36	295	ct						2						
MK36	304	ct						1						
MK36	369	ct							1					
MK36	439	ct		1										
MK36	460	ct												No inclusions
MK36	543	ct						1		1				
MK36	587	ct												No inclusions
MK36	728	ct				1								
MK36	1237	ct	2		1		1	3						Solitary spikelet in involucre with torn peduncle
AZ22	totals		0	33	3	27	0	32	2	0	1			
MT25	totals		0	0	1	4	0	1	0	0	0			
MK36	totals		4	2	2	4	3	7	1	1	0

**Table 2:** Summary of data on domestication features, including non-shattering and paired spikelets, based on data from this study and published assemblages from Fuller et al. (2007), Manning et al. (2011)

Site	Involucre w/ torn peduncle	Involuc. w/ smooth base	Involucre base w/bristles (indet)	Paired spikelets	Solitary spikelet	Spikelet (indet)	Median age estimate	Min. % paired (or 2+) spikelets	Mean torn peduncle	Max. torn peduncle	Min. torn peduncle
AZ22		33	3		27	6	4500 BC	0	0%	(0%)	0%
MT25			1		1	6	3500 BC	0	0%	(0%)	0%
Karkarichinkat Nord (KN05)			1			2	2500 BC	0	0%	(0%)	0%
MK36	4	2	2	3	1	7	2200 BC	27.3%	66.7%	83.3%	57.1%
Jsmagamag (JS07)	2		9	1		6	1950 BC	14.3%	(100%)	100%	18.2%
Ebeleit (EB07)	6		2	1		11	1900 BC	8.3%	100%	100%	0.75%
Er Negf (EN07)	2	1	8	2		5	1800 BC	28.6%	67.7%	90.9%	18.2%
Djiganyai	1		2	3			1795 BC	100%	66.7%	100%	33.3%
Oued Bou Khzama	1		1	3			500 BC	100%	75%	100%	50%

**Fig. 4:**
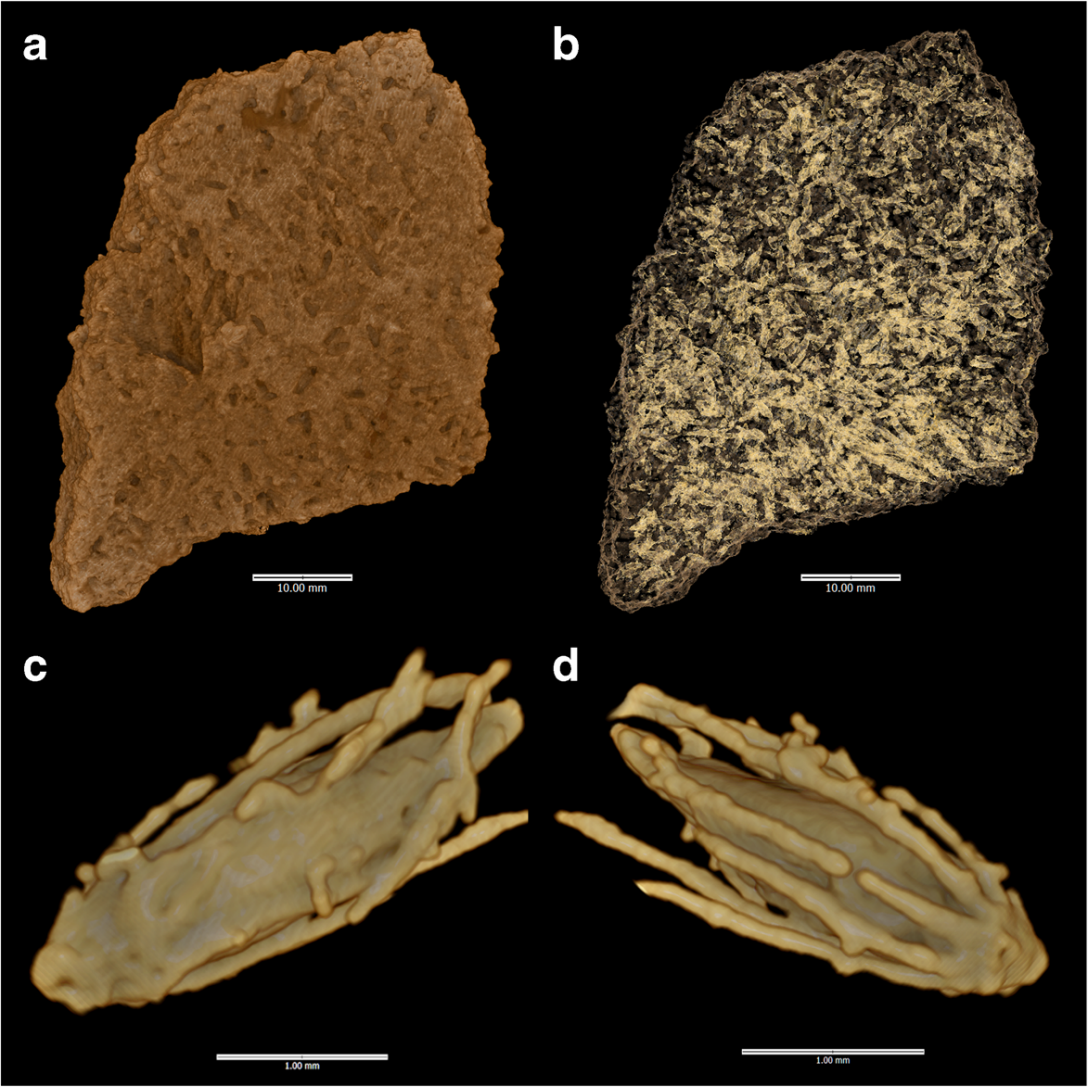
MicroCT visualizations for AZ22, sherd 27: **a** whole-sherd exterior surface; **b** all organic inclusion casts. Lower row presents dorsal (**c**) and ventral views (**d**) of a sessile (wild-type) involucre cast (Inclusion 1) containing a single spikelet. For more examples, see Supplementary Images.

**Fig. 5:**
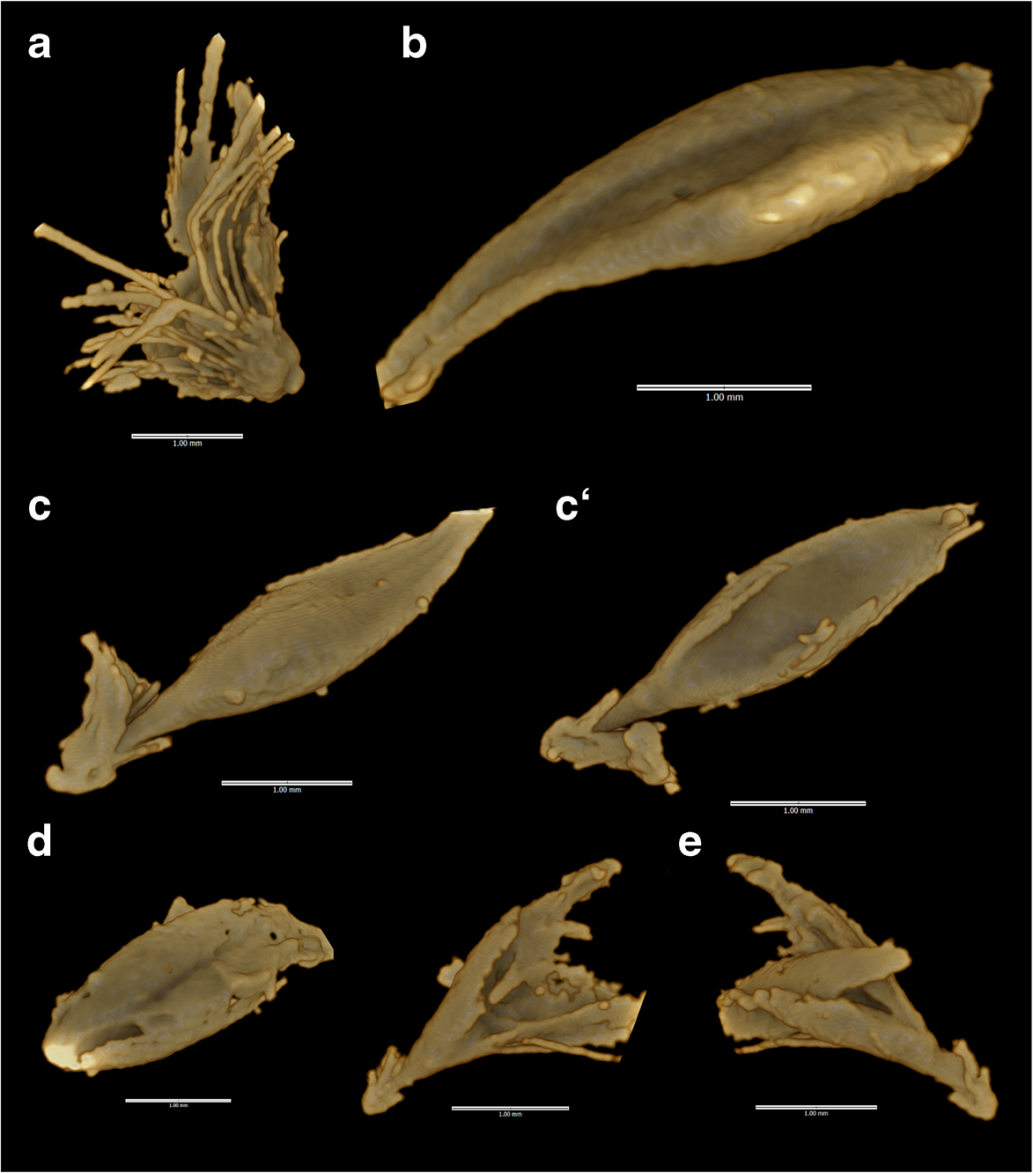
MicroCT visualizations for MK36, sherd 1237: **a** stalked involucre with bristles (nonshattering, domesticated type) (Inclusion 1); **b** spikelet (Inclusion 3); **c** spikelet in involucre, seen in lateral view, suggesting paired spikelet form torn involucre stalk (**c**′ = ventral view of the same) (Inclusion 11); **d** single spikelet (Inclusion 12); **e** two rotated views of spikelet in involucre, suggesting solitary spikelet and sessile (wild type) involucre (Inclusion 13). For the complete dataset, see Supplementary Images

Results from microCT-scanning and visualization of voids confirm intensive use of pearl millet chaff tempering and the presence of purely shattering wild-type involucres. Many hundreds of spikelets and involucres are present within sherd 27 from AZ22 (Fig. 4). A subsample of 30 complete spikelets was selected for detailed imaging and three-dimensional assessment. The results indicated single grained spikelets within bristles of the involucre; and smooth-based, nonstalked involucres that are morphologically wild (Fig. 5). Combining the microCT and surface cast datasets, we documented 31 wild type involucres and 26 examples of solitary spikelets, but no example of domesticated (stalked) involucre or paired spikelets. The archaeobotanical data are thus consistent with a purely wild pearl millet population.

It should be noted that the grain shape of these impressions is narrow and lacks the thickened, club-shaped morphology that is typical of domesticated grains (see D'Andrea et al. 2001; Manning and Fuller 2014; Zach and Klee 2003). Twenty-eight measurements, representing maximum grain breadth, were obtained from spikelet impressions and the microCT visualizations to study grain sizes. Grain breadths ranged from 0.69 mm to 1.08 mm, with an average of 0.89 mm, which falls in the range expected of wild millet, i.e., 0.5–1 mm (Fuller 2007; Zach and Klee 2003), and the average is 0.80 mm when reduced by 10% (to make them comparable to charred material).

### MT25

Only a few sherds from the assemblage at MT25 exhibited obvious chaff-tempering. Of these, two sherds were cast for surface impressions (Tables 1 and 2). These showed obvious *Pennisetum* bristles, a single involucre apex with bristle bases and a solitary spikelet, and five additional spikelets, but there are no involucre base remains that demonstrate domestication status. Breadth measurements on these spikelets ranged from 0.7 mm to 1.2 mm (average of 0.97 mm). While the average falls within the range of modern wild *P. violaceum*, the upper part of the range (>1 mm) suggests a deviation from the expected wild range. The mean is 0.87 mm when reduced by 10%, which is comparable to the reported average of early domesticated carbonized grains (~0.9 mm) (D'Andrea et al. 2001; Zach and Klee 2003).

### MK36

From MK36, ten sherds were chosen based on the promising surface impressions. These were studied through microCT scanning (Tables 1 and 2). Out of seven involucres within the resultant visualized assemblage, four had torn peduncles of the domesticated type, while two showed stalkless, dehiscent types (Fig. 5). In three cases, paired spikelets were indicated, while one had a solitary spikelet. Seven additional spikelets were indeterminate as to pairing. Therefore, at a minimum of 27% (3 out of 11), spikelets came from pairs. Breadth could be measured on nine visualized spikelets, giving a 0.93–1.53 mm range (average 1.14 mm), and the mean is 1.03 mm when reduced by 10%, indicating that these grains fall clearly within the domesticated size range. The pearl millet data from these three sites are summarized in Table 2 alongside similar data published from Mali and Mauritania.

## Discussion and Further Analysis

In the fifth millennium BC in northern Mali, represented by AZ22, the overall plant assemblage suggests that *Pennisetum* dominated other small grasses that were incorporated into some sherds. The AZ22 assemblage provided a point in time and space when the collection of wild *Pennisetum* was an important part of the food economy, and its processing produced abundant chaff that was utilized as ceramic temper. The AZ22 data is part of a small but growing evidence of wild *Pennisetum* utilization in Africa. The evidence has also been reported from southwest Libyan sites of the late Acacus period (6500–5000 BC), including Ti-n-Torha, Uan Muhuggiag, and UanTabu, where charred seeds of wild *Pennisetum* were recovered (Mercuri 2001; Wasylikowa 1993), and Takrakori where desiccated *Pennisetum* grains and chaff were found (Mercuri et al*.* 2018). However, these cases plausibly represent the desert grass, *P. divisum*, rather than the crop progenitor *P. violaceum*. Taken together, though, these finds point to traditions of wild *Pennisetum* harvesting across the central and western Sahara during the Early to Middle Holocene, which could constitute the cultural background for the later cultivation of *Pennisetum violaceum* and its subsequent domestication (Dupuy 2014; Manning and Fuller 2014). In the case of the Acacus region of Libya, however, *Pennisetum* sp. drops out of use by ca. 6200 BC, with subsequent plant exploitation and probable cultivation focusing on small millets, such as *Panicum* and *Echinochloa* (Mercuri et al. 2018). Indeed, Winchell et al. (2018) suggest three distinct, yet parallel, pathways to cereal cultivation in Africa that were each initially based on different grasses: small millet cultivation in the central Sahara, sorghum in the eastern Sahel, and pearl millet in the western Sahel.

**Fig. 6:**
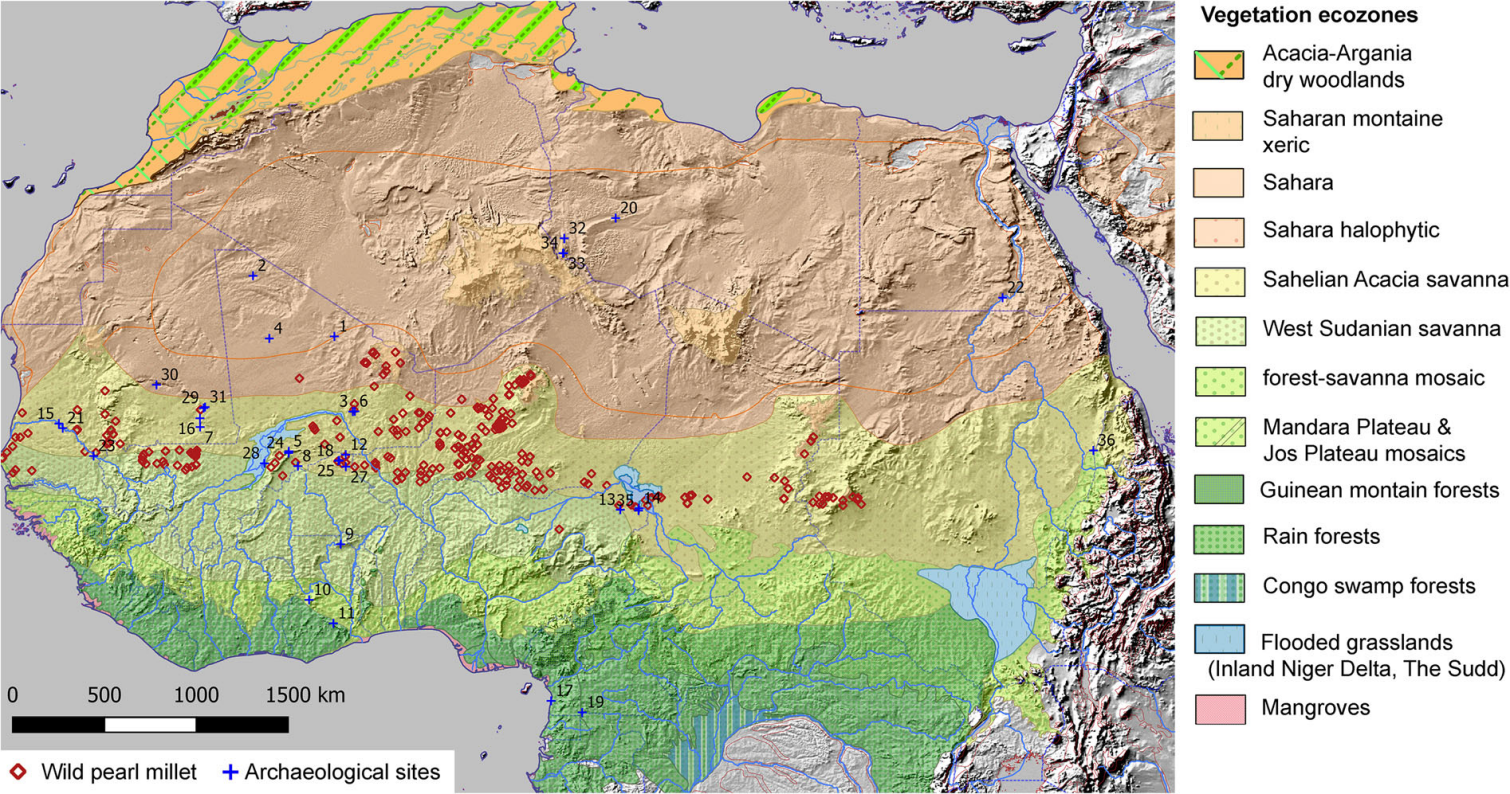
Map of distribution of modern wild pearl millet in relation to modern vegetation zones (after Olson et al*.* 2001), also showing locations of archaeological sites with early *Pennisetum* discussed in this article (numbers correspond to those in Table 3). For sources of ecozone boundaries and wild pearl millet, see “Acknowledgements”. The coordinates of modern wild pearl millet populations are in Supplementary Table S1; and archaeological sites with wild pearl millet are in Supplementary Table S2.

Wild pearl millet was plausibly a grain staple millennia before the earliest evidence of its domestication in the later third millennium BC. From this time onward, morphological changes that are characteristic of the domestication process are documented at many sites, including MT25 and MK36. These data support the initiation of pearl millet cultivation and domestication in the western Sahara desert/Sahel region, in what is today northern Mali. This archaeobotanical interpretation is consistent with a recent genomic study about the location of pearl millet’s initial domestication (Burgarella et al. 2018). It appears that the initial exploitation of pearl millet took place north of its modern wild range (Fig. 6). This northerly location accorded with the expected northward displacement of grasslands during the African Humid Period, which ended around 5000 years ago (De Menocal 2015; Manning and Timpson 2014). As the Sahara expanded and the Sahelian vegetation zone retreated south, pearl millet would have already been under early predomestication cultivation.

The fourth millennium BC dataset from site MT25 is consistent with a morphologically wild population, although it is a very small sample. Grain breadth here was larger on average than recorded at AZ22, marked by an increase in the mean grain breadth of ~28%. The latest data from MK36 indicates that the pearl millet that was being exploited in far northern Mali during the later third millennium BC was undergoing domestication. This site lies north of both the modern wild distribution (Fig. 6) and around the northern limits of traditional cultivation (Bindinger et al. 1982; Wotzka 2019), suggesting that domestication of pearl millet initially took place in the southwestern part of the “Green Sahara,” prior to final aridification during the middle Holocene (De Menocal 2015). Nonshattering forms were present, which exhibited stalked involucres that are expected to exist and be selected only in contexts of routine cultivation and human harvesting. However, the cooccurrence of these domesticated morphotypes with wild morphotypes could indicate that these populations were in the middle of the domestication episode. In addition, paired spikelets indicate selection for this trait, which increases yield per spike. In wild populations, multigrain involucres are expected to be rare as they will make diaspores less aerodynamic and less easily dispersed and buried, whereas human harvesting removed any deleterious consequences for the plant. Also, grain breadth had increased in terms of average size and maximum breadth by comparison to the earlier data from AZ22 and MT25. The average grain breadth at MK36 was ~38%, larger than AZ22.

The archaeobotanical evidence from MK36 suggests an ongoing domestication process. The insights from the better-studied regions like southwest Asia indicate that the MK36 materials were in the “Entrenched Pre-Domestication Cultivation” phase. During this period, domestication traits like nonshattering occur at a rate of 20–80% in the cereal population, and grain sizes increase on average by 20–40% (Fuller et al. 2018). Based on such southwest Asian comparisons, we can hypothesize that cultivation had likely begun approximately 1000–1500 years earlier, by ~4000 BC, possibly overlapping with or just postdating the AZ22 evidence.

In order to assess the wider domestication process of pearl millet, we can consider these new data alongside previously published evidence. This includes previous work on ceramic impressions that provide some information on chaff features, including nonshattering involucres and paired/multiple spikelets (Table 2). Prior work includes evidence from the Lower Tilemsi Valley, where eight out of the nine diagnostic impressions were of the domesticated type around the end of the third millennium BC (Manning et al. 2011). In addition, evidence from Mauritania (1900–1500 BC), although not documented quantitatively, indicates the predominance of stalked, nonshattering involucres and double-grained involucres (Amblard and Pernès 1989; Fuller et al. 2007; MacDonald et al. 2009).

**Fig. 7:**
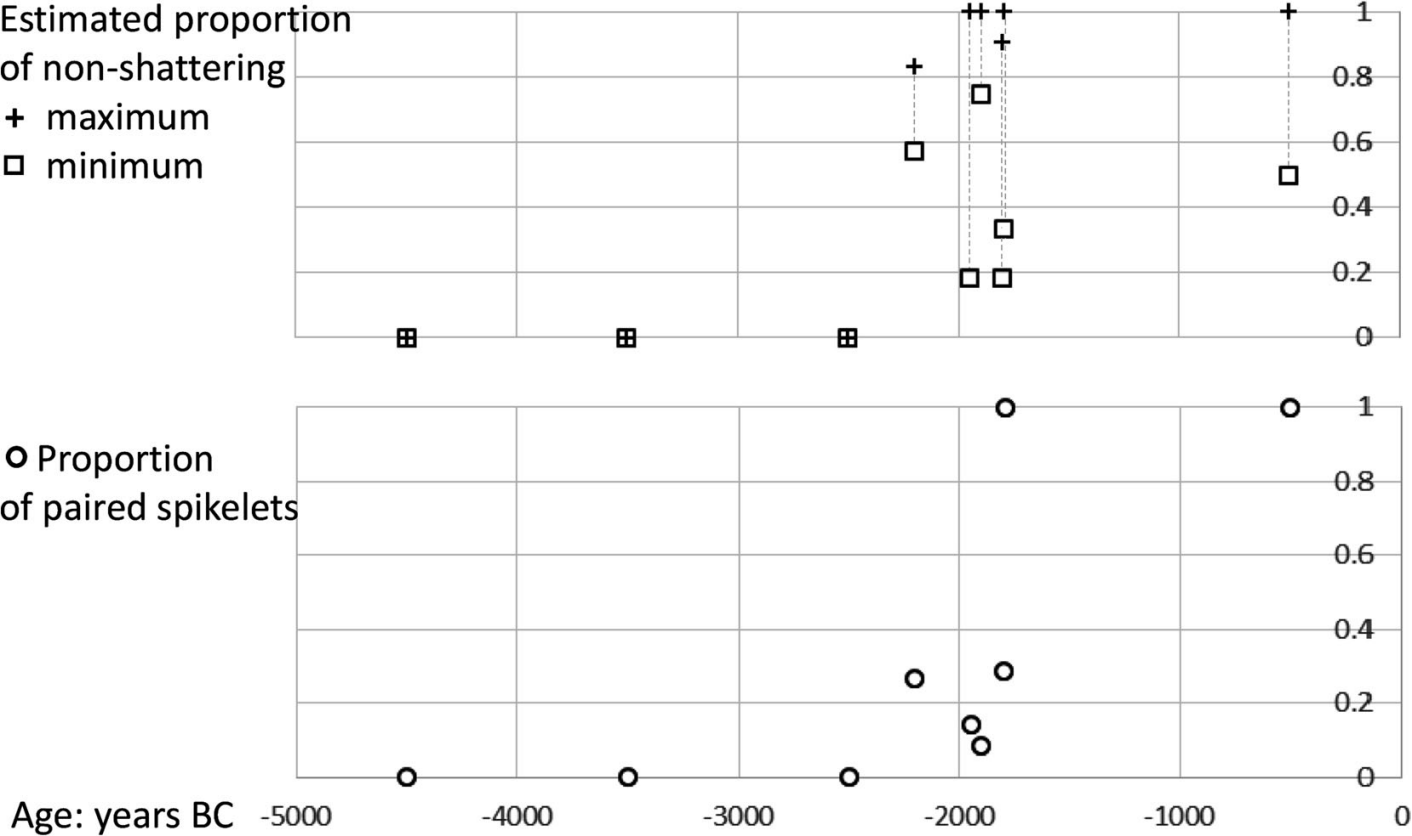
Trends in domestication traits based on early impression data, plotted against median calendrical age. Upper graph indicates maximum and minimum estimates of the proportion of non-shattering (stalked involucres). Lower graph indicates observed proportion of paired spikelets. Data: Table 2

The limited quantitative data for shattering/nonshattering involucres have been used to plot estimates of the minimum and maximum nonshattering proportions (taking into account indeterminate remains), using median age estimates for each site (Fig. 7: top). In addition, the proportion of paired as opposed to solitary spikelets has also been estimated, which similarly indicates divergence from solitary spikelets before the end of the third millennium BC and a dominance of paired spikelets from the first half of the second millennium BC (Fig. 7: bottom). Although the data are limited, the trends from both eastern Mauritania and the Lower Tilemsi Valley in Mali indicate the end of the domestication process, in terms of selection for nonshattering, took place between the third and second millennium BC. Both regions seem to have received, from earlier cultural traditions such as those represented by MK36 and MT25, cultivated pearl millet that was undergoing domestication.

**Fig. 8:**
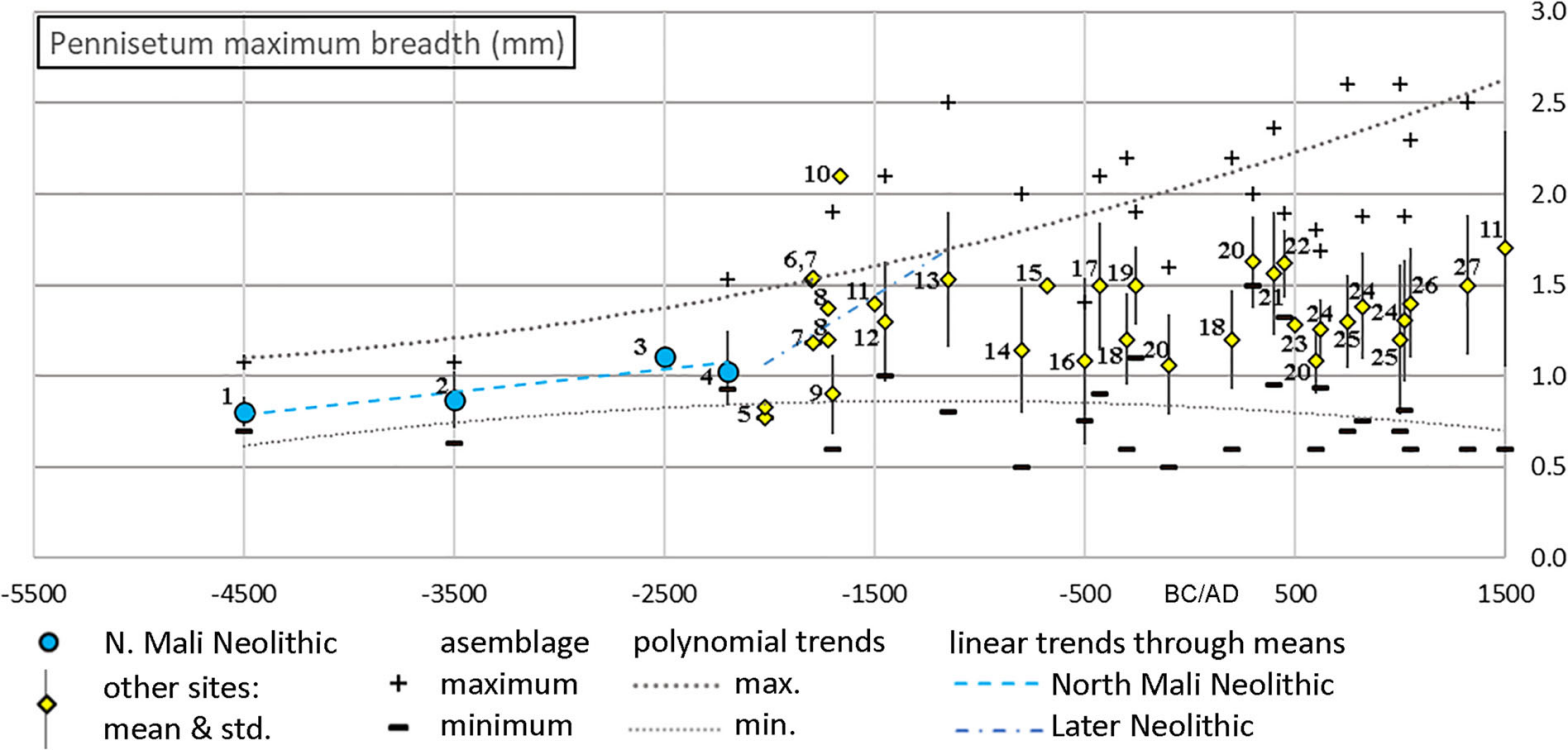
Grain size change in archaeological pearl millet: polynomial trends lines through the maximum and minimum archaeological grain sizes have been determined by default settings in Microsoft Excel. Linear regression lines illustrate two phases of evolutionary trends in average grain size increase, during domestication (based on AZ22, MT25, MK36 and KN05) and subsequent secondary enlargement (based on MK36, Er Negf, Ounjougou, Brimi, Bosumpra Cave, Ti-n-Akof and Ganjiganna). Impression measurements and desiccated grains (Qasr Ibrim) have been reduced by 10% to make them more comparable to charred material. Sites numbered as per Table 3

In order to further track the domestication and postdomestication evolutionary processes in pearl millet, we reviewed the evidence for grain size change (Fig. 8). We have compiled published measurements and, when not available, have made new estimates from published images with scales (Table 3). By recording means, standard deviations, and assemblage extremes (maximum and minimum) and plotting these against median ages for a site or site phase, long term trends are evident. Impressions and desiccated grains were reduced by 10% to make measurements more comparable to charred grains (Fuller 2007). Spikelet maximum breadth, taken from surface impressions, is regarded as equal to the breadth of grains. While such data can be expected to incorporate some noise due to the vagaries of carbonization, we can still expect to record long-term trends in average size and range (Bonhomme et al. 2016; Fuller et al. 2014, 2017). Our total African dataset of pearl millet grain metrics comprises 2736 measurements, dating from ca. 4500 BC to 1500 AD, including assemblages from 27 sites across mainly the western African region (Fig. 1). Although the sample size for many assemblages is small, these data nevertheless allow some assessment of long-term chronological trends in pearl millet grain size and determine how this trait evolved in relation to domestication and postdomestication evolution (Fig. 8).

**Table 3:** Summary of grain size (maximum breadth) data from archaeological pearl millet across western and Saharan Africa. Desiccated grins (Qasr Ibrim) and estimates from pottery impression spikelets corrected by −10% to improve comparability to charred grains. In some cases measurements have been estimated from photographs in the original source

No	Site	Median age (-BC/ AD)	Breadth Ave	Stdev	Min	Max	*n*	Source
1	AZ22	−4500	0.803	0.082	0.692	1.077	28	This study
2	MT25	−3500	0.870	0.158	0.630	1.080	6	This study
3	Karkarichinkat Nord (KNO5)	−2500	1.110			1.110	1	Manning and Fuller (2014)
4	MK36	−2200	1.046	0.208	0.931	1.526	9	This study
5	Winde Koroji	−2025	0.770		0.770		1	MacDonald et al. (2017)
5	Winde Koroji	−2025	0.825			0.825	1	MacDonald et al. (2017)
6	Er Negf (EN07)	−1800	1.530			1.530	1	Manning and Fuller (2014)
7	Djiganyai	−1795	1.54			1.540	1	Fuller et al. (2007)
7	Djiganyai	−1795	1.181		1.181		1	Fuller et al. (2007)
8	Ounjougou	−1725	1.371			1.371	1	Ozainne et al. (2009)
8	Ounjougou	−1725	1.200				1	Ozainne et al. (2009)
9	Birimi	−1700	0.900	0.220	0.600	1.900	376	D'Andrea et al. (2001)
10	Boase: B5C	−1665	2.100			2.100	1	D'Andrea et al. (2006)
11	Bosumpra cave	−1500	1.400				1	Oas et al. (2015)
12	Ti-n-Akof	−1450	1.300	0.330	1.000	2.100	18	Kahlheber (2004)
13	Gajiganna	−1150	1.530	0.374	0.800	2.500	30	Kahlheber (2004)
14	Kursakata (weighted ave)	−800	1.142	0.346	0.500	2.000	41	Zach and Klee (2003)
15	Walalde	−675	1.500				1	Murray and Déme (2014)
16	Oued Bou Khzama	−500	1.081	0.457	0.758	1.404	3	After Fuller et al. (2007)
17	Bwambe-Sommet	−425	1.500	0.346	0.900	2.100	15	Kahlheber et al. (2009)
18	Oursi West	−300	1.200	0.252	0.600	2.200	800	Kahlheber (2004)
19	Abang Minkoo	−260	1.500	0.214	1.100	1.900	18	Kahlheber et al. (2009)
20	Old Jarma	−100	1.062	0.278	0.500	1.600	21	Pelling (2007); Fuller et al. (2007)
18	Oursi West	200	1.200	0.270	0.600	2.200	400	Kahlheber (2004)
20	Old Jarma	300	1.625	0.250	1.500	2.000	4	Pelling (2007); Fuller et al. (2007)
21	Cubalel	400	1.562	0.340	0.951	2.360	35	New metrics; cf. Murray et al. (2007)
22	Qasr Ibrim	450	1.620	0.185	1.320	1.890	10	Steele and Bunting (1982)
23	Arondo	500	1.280				1	Zach and Klee (2003)
20	Old Jarma	600	1.082	0.176	0.600	1.800	254	Pelling (2007); Fuller et al. (2007)
24	Tongo Maare Diabal (1–2)	625	1.256	0.166	0.938	1.688	41	Champion (2019)
25	Oursi North	750	1.300	0.256	0.700	2.600	60	Kahlheber (2004)
24	Tongo Maare Diabal (3)	825	1.383	0.292	0.750	1.875	25	Champion (2019)
25	Oursi North	1000	1.200	0.410	0.700	2.600	262	Kahlheber (2004)
24	Tongo Maare Diabal (4–5)	1025	1.304	0.333	0.813	1.875	30	Champion (2019)
26	Saouga	1050	1.400	0.300	0.600	2.300	90	Kahlheber (2004)
27	Toguere Doupwil	1325	1.500	0.385	0.600	2.500	143	Bedaux et al. (1978)
11	Bosumpra Cave	1500	1.700	0.646	0.600	4.000	5	Oas et al. (2015)

Three major patterns can be discerned in the pearl millet grain size data (Fig. 8). First, there is a clear trend for the average grain breadth to increase in size from the beginning of the time series: from 0.8 mm before 4000 BC to between 1.3 mm and 1.7 mm after AD 750. The overall trend in increasing size is statistically significant. The trend was tested with a Mann–Kendall Trend test, which tests for directional (monotonic) trends in time series data (Mann 1945), performed in Past 3.1 software (Hammer et al. 2001). The test shows that the trend is significantly directional (*p*=0.0026798 for a nondirectional, random trend). Second, the overall variation in size around the mean increases over time, a phenomenon also noted in grain size data for wheat and barley (Fuller et al. 2017). Third, the upper range—maximum breadth measurements—tends toward larger sizes, whereas the minimum sizes remain about the same (note polynomial trend lines in Fig. 8). Taking the upper limits of grain size, one can contrast the maximum size of ~1.5 mm at ca. 2200 BC with ~2.1 mm at ca. 1660 BC and increasing to 2.5 mm by 1100 BC.

Considering the earlier part of this time series in more detail, we can suggest two phases in the trend for size increase: (1) an early phase of the Later Stone Age trend (4500–2200 BC), that we associate with the initial domestication process, in which there were size increases of ~0.01% per year; and (2) a terminal phase of the Later Stone Age size trend (2500–1150 BC), in which the rate of increase became ~0.04% per year. These two phases of size change can be translated into estimates of *Haldane* rate, which measures the shift in the distribution of a phenotypic trait per generation (see Purugganan and Fuller 2011) from ~6.4×10^−3^ haldanes (earlier) to 11.4×10^−3^ (later). These rates of size increase are comparable to the rates of change estimated for several other grain domesticates (Fuller et al. 2014). Subsequent to this, there were no directional trends in the average, but rather an interassemblage variation with means in breadth fluctuating between ~1.1 mm and ~1.7 mm. Similar interassemblage variations have been observed in wheat and barley grain sizes in Europe and West Asia, which show a directional increase during the Pre-Pottery Neolithic (9000–6000 BC) and subsequently much more variation in the mean and maximum size (6000–3000 BC) during postdomestication (Fuller et al. 2017).

The interassemblage variation in pearl millet after ca. 1500 BC shows evidence of postdomestication diversification. Recent work on domestication genetics has highlighted distinct evolutionary phases of domestication and diversification (Meyer and Purugganan 2013), with the latter including genetic changes and adaptations particular to some regional populations of the crop. Significant evolution usually occurred as crops were translocated and adapted to a diverse geographical and cultural distribution (Fuller and Lucas 2017; Gepts 2014). The four cultivated races, with differing geographies, are characterized by differences in grain shape (Brunken et al. 1977), indicating that postdomestication evolution indeed involved grain size variation. None of these varieties became as small and thin as wild forms, although long, thin grains characterize race Leonis in western coastal Africa (e.g., Sierra Leone). As pearl millet spread from the Sahelian range of its wild progenitor (95% of wild populations occur from 12.4°–19° N), it encountered much higher rainfall levels and differing seasonality. Its dispersal into the equatorial wet tropics brought about selection for loss of day-length sensitivity. This necessitated the development of early-flowering varieties that could be sown and harvested before the peak of rainfall in late summer instead of late-flowering varieties that share with the wild form flowering tied to shortening days at the end of summer (Clotault et al. 2012; Dussert et al. 2015). While modern genomic studies have revealed many other loci that underwent selection as regional varieties differentiated, such as spike length (related to yield), root system size (related to drought tolerance), and bristle length (related in resistance to some insects), it remains the case that “the genetic basis of environmental adaptation in pearl millet has not been well understood” (Serba et al. 2020).

There are likely trends toward larger grains of pearl millet varieties that spread south into the wetter tropics and desert oases. In the dataset reviewed here, sites south of Latitude 10° N have above-average grain breadths (>1.29 mm, e.g., Abang Minko, Bwambe-Sommet, Bosumpra, Boase B5C). The same is for sites in desert latitudes such as Old Jarma and Qasr Ibrim, which might reflect varieties adapted to cultivation with irrigation. Previously identified variation in Indian archaeological pearl millet (Fuller 2007; Manning et al. 2011), between smaller grains in semiarid western India versus much plumper grains in the better-watered Ganges plains, could suggest a similar pattern. These cases support the hypothesis that pearl millet varieties grown under and adapted to wetter conditions had more plump grains, on average. It is unknown at this stage how much of this is underpinned by genetic changes as opposed to phenotypic plasticity, which is also increasingly discussed as a potential factor in some changes under cultivation (Denham et al. 2020; Larson et al. 2014; Piperno 2017). The data compiled in this study provide an initial and partial understanding of the trends of grain size change connected to domestication due to the wide dispersal and diversification of pearl millet. They call for systematic recording of grain size across many more sites.

## Conclusions

The research on pearl millet impressions and inclusions in ceramics highlights the great potential of studying ceramic assemblages in regions like Northern Mali for chaff-tempering remains. Such data may provide crucial evidence on past plant use and regional pathways to agriculture. This study also highlights how much additional data becomes available by using new microCT scanning methods to look inside sherds (as per Barron et al. 2020a; Barron et al. 2020b). This offers much greater whole-sherd, quantitative potential for archaeobotanical studies than the traditional study of impressions by surface casting. By reanalyzing ceramic collections from northern Mali, using new methods, we have generated new data on pearl millet domestication.

Our evidence indicates that wild pearl millet was being exploited in northern Mali in the fifth millennium BC and that predomestication cultivation was probably established sometime in the fourth millennium BC. The appearance of morphological domestication traits is clearly established by the middle of the third millennium BC. The domesticated pearl millet was introduced to adjacent regions, including the Hodh depression of Mauritania and the Lower Tilemsi Valley in Mali toward the end of the third millennium BC; and southern Mali, northern Ghana, and the Lake Chad region by the first half of the second millennium BC. These data provide an improved understanding of how pearl millet evolved domestication traits, including nonshattering spikes, paired/multiple spikelets, and larger grain sizes. Grain metrics indicate a general trend of increasing grain breadth during predomestication cultivation (4500–2500 BC), with acceleration in average size increase from the mid-third millennium BC to the second millennium BC. This trend toward increasing average grain size and the upper limits of size range is, therefore, a reasonable proxy for inferring the domestication process. However, the increasing variability in grain size and other modalities of diversification through time was also affected by the dispersal of the crop to different ecologies. We have also been able to update and improve estimates for the evolutionary rate of grain size increase in pearl millet, making it the best-documented indigenous plant domestication process in Africa.

## Supplementary Information

ESM 1 (DOCX 266 kb)

ESM 2 (PDF 7241 kb)
