# Supplementary material for: Transition From Wild to Domesticated Pearl Millet (Pennisetum glaucum) Revealed in Ceramic Temper at Three Middle Holocene Sites in Northern Mali
Source: Afr Archaeol Rev. 2021 Mar 16;38(2):211–30. doi: 10.1007/s10437-021-09428-8 (PMC8550313; doi:10.1007/s10437-021-09428-8)
Supplement: Supplementary file 1 — (DOCX 266 kb) [file 10437_2021_9428_MOESM1_ESM.docx]

**Transition from wild to domesticated pearl millet (*Pennisetum glaucum)* revealed in ceramic temper at three Middle Holocene sites in Northern Mali**

Additional Supplementary Information: S1, S2, S3

**Table S1. Wild pearl millet collection accession coordinates**. IP refers to accession number in the International Research Institute for the Semi-Arid Tropics (ICRISAT), Hyderabad, India. Data from the GENESYS Global Portal on Plant Genetic Resources, <http://www.genesys-pgr.org> [8 April 2020]

| Accession No. | Longitude | Latitude |
| --- | --- | --- |
| IP 21704 | 10.3 | 11.3 |
| IP 21703 | 14.03 | 12.4 |
| IP 21702 | 14.03 | 12.4 |
| IP 22019 | 24.18 | 12.47 |
| IP 21699 | 13.22 | 12.47 |
| IP 21698 | 13.22 | 12.47 |
| IP 21536 | 14.63 | 12.47 |
| IP 21535 | 14.63 | 12.47 |
| IP 21700 | 13.82 | 12.5 |
| IP 21701 | 13.82 | 12.5 |
| IP 21738 | 25.08 | 12.53 |
| IP 22018 | 23.19 | 12.54 |
| IP 22017 | 23.02 | 12.56 |
| IP 21718 | 16.58 | 12.57 |
| IP 21717 | 16.58 | 12.57 |
| IP 22159 | 16.56 | 12.58 |
| IP 21531 | 22.72 | 12.62 |
| IP 21737 | 24.18 | 12.72 |
| IP 21534 | 14.53 | 12.73 |
| IP 21740 | 24.85 | 12.78 |
| IP 21739 | 24.85 | 12.78 |
| IP 21723 | 17.57 | 12.78 |
| IP 21724 | 17.57 | 12.78 |
| IP 22016 | 21.29 | 12.8 |
| IP 21726 | 21.29 | 12.8 |
| IP 21725 | 21.29 | 12.8 |
| IP 21727 | 21.3 | 12.81 |
| IP 21705 | 14.76 | 12.83 |
| IP 21741 | 24.92 | 12.85 |
| IP 21736 | 23.7 | 12.85 |
| IP 21735 | 23.7 | 12.85 |
| IP 21707 | 14.83 | 12.85 |
| IP 21706 | 14.83 | 12.85 |
| IP 22013 | 15.29 | 12.86 |
| IP 21711 | 15.29 | 12.86 |
| IP 21712 | 15.29 | 12.86 |
| IP 21713 | 16.45 | 12.87 |
| IP 21714 | 16.45 | 12.87 |
| IP 21742 | 24.67 | 12.9 |
| IP 21734 | 23.47 | 12.9 |
| IP 21733 | 23.47 | 12.9 |
| IP 21732 | 23.32 | 12.9 |
| IP 21731 | 23.03 | 12.93 |
| IP 21710 | 15.16 | 12.93 |
| IP 21709 | 15.16 | 12.93 |
| IP 22015 | 16.69 | 12.95 |
| IP 22014 | 16.69 | 12.95 |
| IP 21715 | 16.69 | 12.95 |
| IP 21716 | 16.8 | 12.98 |
| IP 21687 | 9.109999 | 13.21 |
| IP 21689 | 9.520001 | 13.27 |
| IP 21721 | 21.5 | 13.3 |
| IP 21720 | 21.5 | 13.3 |
| IP 21719 | 21.5 | 13.3 |
| IP 21681 | 7.480001 | 13.33 |
| IP 21978 | 8.670001 | 13.36 |
| IP 21977 | 8.279999 | 13.36 |
| IP 22005 | 12.63 | 13.45 |
| IP 21976 | 7.71 | 13.46 |
| IP 21686 | 8.729999 | 13.46 |
| IP 21523 | 11.71 | 13.53 |
| IP 21609 | 11.53 | 13.62 |
| IP 21545 | 3.5 | 13.63 |
| IP 21722 | 21.33 | 13.67 |
| IP 21679 | 7.329999 | 13.67 |
| IP 21685 | 8.369999 | 13.7 |
| IP 21680 | 7.73 | 13.7 |
| IP 21544 | 2.369999 | 13.7 |
| IP 21546 | 3.779999 | 13.78 |
| IP 21543 | 2.589999 | 13.8 |
| IP 21980 | 7.010001 | 13.82 |
| IP 22160 | 20.83 | 13.83 |
| IP 21708 | 19.49 | 13.87 |
| IP 21642 | 5.349999 | 13.88 |
| IP 21691 | 9.329999 | 13.89 |
| IP 21591 | 5.519999 | 13.93 |
| IP 21530 | -3.23 | 13.93 |
| IP 21678 | 6.9 | 13.95 |
| IP 21547 | 3.519999 | 13.95 |
| IP 21684 | 8.5 | 14.01 |
| IP 21606 | 11.88 | 14.03 |
| IP 21979 | 7.900001 | 14.04 |
| IP 21537 | 3.970001 | 14.04 |
| IP 21985 | 5.949999 | 14.05 |
| IP 21590 | 6 | 14.05 |
| IP 21692 | 9.859999 | 14.06 |
| IP 21665 | -16.4232 | 14.0765 |
| IP 21542 | 2.779999 | 14.1 |
| IP 21541 | 2.390001 | 14.1 |
| IP 21690 | 9.180001 | 14.12 |
| IP 21682 | 7.789999 | 14.14 |
| IP 21683 | 8.159999 | 14.16 |
| IP 21677 | 7.239999 | 14.16 |
| IP 21548 | 3.539999 | 14.16 |
| IP 21676 | 7.449999 | 14.18 |
| IP 21522 | -15.5 | 14.18 |
| IP 22021 | -7.98 | 14.22 |
| IP 22020 | -7.98 | 14.22 |
| IP 21521 | -16.3 | 14.22 |
| IP 21538 | 4.099999 | 14.27 |
| IP 21561 | 1.330001 | 14.31 |
| IP 21539 | 4.460001 | 14.31 |
| IP 22022 | -7.9 | 14.33 |
| IP 21529 | -3.8 | 14.35 |
| IP 22023 | -7.67 | 14.37 |
| IP 21688 | 0.3 | 14.37 |
| IP 21552 | 0.3 | 14.37 |
| IP 21550 | 6.989999 | 14.38 |
| IP 21986 | 6.25 | 14.42 |
| IP 21675 | 7.840001 | 14.42 |
| IP 22059 | -8.38 | 14.44 |
| IP 21610 | 7.389999 | 14.44 |
| IP 21582 | 0.779999 | 14.45 |
| IP 21556 | 1.299999 | 14.45 |
| IP 21549 | 3.67 | 14.46 |
| IP 22026 | -7.47 | 14.47 |
| IP 22025 | -7.47 | 14.47 |
| IP 22024 | -7.47 | 14.47 |
| IP 22058 | -9.02 | 14.48 |
| IP 22057 | -9.02 | 14.48 |
| IP 21585 | 5.400001 | 14.48 |
| IP 21983 | 1.299999 | 14.5 |
| IP 22054 | -9.92 | 14.51 |
| IP 21641 | 5.349999 | 14.52 |
| IP 22055 | -9.38 | 14.53 |
| IP 21553 | -0.33 | 14.53 |
| IP 21528 | -3.61 | 14.55 |
| IP 21984 | 1.299999 | 14.57 |
| IP 21559 | 1.299999 | 14.57 |
| IP 21557 | 1.299999 | 14.57 |
| IP 21558 | 1.299999 | 14.57 |
| IP 21982 | -0.15 | 14.58 |
| IP 21554 | -0.15 | 14.58 |
| IP 21526 | -2.6 | 14.59 |
| IP 22004 | 8.770001 | 14.6 |
| IP 22056 | -9.08 | 14.63 |
| IP 22053 | -10.07 | 14.63 |
| IP 22052 | -10.07 | 14.63 |
| IP 21981 | -0.53 | 14.64 |
| IP 21551 | -0.53 | 14.64 |
| IP 21524 | 8.470001 | 14.65 |
| IP 22051 | -10.09 | 14.7 |
| IP 21589 | 6.42 | 14.7 |
| IP 21588 | 6.280002 | 14.75 |
| IP 21586 | 5.730001 | 14.75 |
| IP 21587 | 5.769999 | 14.75 |
| IP 21663 | -16.97 | 14.8 |
| IP 21662 | -16.93 | 14.8 |
| IP 21540 | 2.049999 | 14.8 |
| IP 22012 | -17.0523 | 14.8023 |
| IP 21743 | 6.019999 | 14.82 |
| IP 22050 | -10.08 | 14.85 |
| IP 22049 | -10.08 | 14.85 |
| IP 22027 | -7.49 | 14.86 |
| IP 21608 | 8.869999 | 14.87 |
| IP 21607 | 8.869999 | 14.87 |
| IP 21605 | 8.869999 | 14.87 |
| IP 21560 | 1.919999 | 14.87 |
| IP 21555 | -0.17 | 14.87 |
| IP 21527 | -3.39 | 14.92 |
| IP 21584 | 5.550001 | 14.95 |
| IP 22048 | -10.02 | 14.98 |
| IP 22047 | -10.02 | 14.98 |
| IP 22035 | -7.55 | 14.98 |
| IP 22034 | -7.52 | 14.98 |
| IP 22033 | -7.52 | 14.98 |
| IP 22029 | -7.46 | 14.99 |
| IP 22028 | -7.46 | 14.99 |
| IP 21730 | 22.3 | 15 |
| IP 22037 | -7.67 | 15.01 |
| IP 22036 | -7.67 | 15.01 |
| IP 22003 | 8.479999 | 15.01 |
| IP 22002 | 8.479999 | 15.01 |
| IP 22032 | -7.53 | 15.05 |
| IP 22041 | -7.73 | 15.07 |
| IP 22038 | -7.73 | 15.07 |
| IP 21655 | -12.37 | 15.07 |
| IP 21661 | -16.72 | 15.08 |
| IP 22046 | -9.78 | 15.1 |
| IP 21525 | 5.670001 | 15.1 |
| IP 22031 | -7.48 | 15.12 |
| IP 21658 | -12.9 | 15.12 |
| IP 22044 | -8.96 | 15.14 |
| IP 22045 | -8.96 | 15.14 |
| IP 22042 | -8.96 | 15.14 |
| IP 22043 | -8.96 | 15.14 |
| IP 22030 | -7.43 | 15.18 |
| IP 22040 | -8.28 | 15.19 |
| IP 22039 | -8.28 | 15.19 |
| IP 21573 | 5.530001 | 15.25 |
| IP 21574 | 5.530001 | 15.25 |
| IP 21579 | 6.110001 | 15.27 |
| IP 21578 | 6.110001 | 15.27 |
| IP 21744 | 5.199999 | 15.28 |
| IP 21581 | 5.800001 | 15.33 |
| IP 21660 | -16.44 | 15.37 |
| IP 21583 | 5.369999 | 15.37 |
| IP 21569 | 4.469999 | 15.37 |
| IP 21568 | 4.48 | 15.43 |
| IP 21576 | 6.300001 | 15.46 |
| IP 21577 | 6.300001 | 15.46 |
| IP 21575 | 6.300001 | 15.46 |
| IP 21580 | 6.030001 | 15.47 |
| IP 21518 | -1.17 | 15.48 |
| IP 21519 | -1.17 | 15.48 |
| IP 22154 | -11.83 | 15.5 |
| IP 22155 | -11.83 | 15.5 |
| IP 22153 | -11.83 | 15.5 |
| IP 21650 | -11.18 | 15.5 |
| IP 21657 | -11.72 | 15.54 |
| IP 21656 | -11.72 | 15.54 |
| IP 21567 | 4.449999 | 15.55 |
| IP 21729 | 22.62 | 15.63 |
| IP 21572 | 6.199999 | 15.8 |
| IP 21664 | -16.3022 | 15.8185 |
| IP 22065 | -0.42 | 15.82 |
| IP 22063 | -0.42 | 15.82 |
| IP 22064 | -0.42 | 15.82 |
| IP 21659 | -16.07 | 15.82 |
| IP 21728 | 22.73 | 15.83 |
| IP 21697 | 22.73 | 15.83 |
| IP 21571 | 5.550001 | 15.83 |
| IP 22149 | -11.83 | 15.92 |
| IP 22150 | -11.83 | 15.92 |
| IP 22151 | -11.83 | 15.92 |
| IP 21629 | 2.400001 | 15.92 |
| IP 21570 | 5.369999 | 15.95 |
| IP 21654 | -11.52 | 15.97 |
| IP 21653 | -11.52 | 15.97 |
| IP 21520 | -15.69 | 16.02 |
| IP 21566 | 4.48 | 16.03 |
| IP 21564 | 4.48 | 16.05 |
| IP 21565 | 4.800001 | 16.08 |
| IP 21674 | 2.799999 | 16.1 |
| IP 21630 | 1.450001 | 16.1 |
| IP 21596 | 6.699999 | 16.12 |
| IP 21645 | -13.3 | 16.15 |
| IP 21666 | 2.369999 | 16.17 |
| IP 21628 | 2.18 | 16.17 |
| IP 22060 | -1.65 | 16.18 |
| IP 21652 | -11.62 | 16.18 |
| IP 21647 | -13.27 | 16.2 |
| IP 21646 | -13.3 | 16.2 |
| IP 21597 | 7.170001 | 16.2 |
| IP 21997 | 7.380012 | 16.24 |
| IP 21599 | 7.300001 | 16.24 |
| IP 21998 | 7.440001 | 16.26 |
| IP 21601 | 7.440001 | 16.26 |
| IP 22061 | -1.7 | 16.27 |
| IP 21673 | 3.430001 | 16.27 |
| IP 21999 | 7.539999 | 16.3 |
| IP 21991 | 6.5 | 16.32 |
| IP 21992 | 6.5 | 16.32 |
| IP 22062 | -1.75 | 16.37 |
| IP 21996 | 7.429999 | 16.37 |
| IP 21993 | 7.219999 | 16.38 |
| IP 21973 | 73.45 | 16.42 |
| IP 21696 | 73.45 | 16.42 |
| IP 21994 | 7.380001 | 16.45 |
| IP 21602 | 8 | 16.5 |
| IP 21672 | 3.630001 | 16.53 |
| IP 21631 | 7.369999 | 16.58 |
| IP 21651 | -11.4 | 16.61 |
| IP 21995 | 7.72 | 16.62 |
| IP 22009 | -0.58 | 16.63 |
| IP 21600 | 7.530001 | 16.68 |
| IP 21627 | 2.2 | 16.7 |
| IP 21671 | 3.880001 | 16.73 |
| IP 21598 | 7.5 | 16.75 |
| IP 21595 | 6.849999 | 16.87 |
| IP 21670 | 3.950001 | 16.88 |
| IP 21649 | -11.92 | 16.95 |
| IP 21745 | 7.929999 | 16.98 |
| IP 21667 | 2.65 | 17 |
| IP 21562 | 4.989999 | 17.01 |
| IP 21563 | 4.989999 | 17.01 |
| IP 21669 | 3.5 | 17.02 |
| IP 21644 | -15.9716 | 17.02 |
| IP 21668 | 3.420001 | 17.03 |
| IP 21990 | 7.530002 | 17.08 |
| IP 22158 | -7.25 | 17.17 |
| IP 22156 | -7.25 | 17.17 |
| IP 22157 | -7.25 | 17.17 |
| IP 21648 | -13.27 | 17.17 |
| IP 21612 | 0.25 | 17.17 |
| IP 21989 | 6.67 | 17.2 |
| IP 21988 | 6.329999 | 17.22 |
| IP 21594 | 6.329999 | 17.22 |
| IP 22010 | 7.030001 | 17.25 |
| IP 21593 | 6.469999 | 17.27 |
| IP 21592 | 6.289999 | 17.29 |
| IP 21611 | 0.280001 | 17.45 |
| IP 21643 | 5.530002 | 17.67 |
| IP 21987 | 7.079999 | 17.7 |
| IP 21613 | 2.999999 | 17.83 |
| IP 22011 | 7.849999 | 17.88 |
| IP 21639 | 7.829999 | 17.88 |
| IP 21640 | 7.869999 | 17.9 |
| IP 22152 | -11.92 | 18.08 |
| IP 21614 | 1.25 | 18.3 |
| IP 21638 | 7.829999 | 18.33 |
| IP 21637 | 8.13 | 18.37 |
| IP 21636 | 8.470001 | 18.43 |
| IP 21635 | 8.550001 | 18.48 |
| IP 21634 | 8.630001 | 18.53 |
| IP 21603 | 8.420001 | 18.55 |
| IP 21633 | 8.380001 | 18.62 |
| IP 22000 | 8.569999 | 18.63 |
| IP 21632 | 8.569999 | 18.63 |
| IP 22066 | -2.41 | 18.69 |
| IP 22001 | 8.920001 | 18.8 |
| IP 21616 | 1.700001 | 18.88 |
| IP 21604 | 8.899999 | 18.88 |
| IP 22006 | 1.830001 | 19.05 |
| IP 21617 | 1.830001 | 19.05 |
| IP 21618 | 1.83 | 19.3 |
| IP 21625 | 0.879999 | 19.43 |
| IP 21624 | 0.879999 | 19.43 |
| IP 22007 | 2.529999 | 19.45 |
| IP 21619 | 2.529999 | 19.45 |
| IP 21623 | 0.769999 | 19.47 |
| IP 21615 | 0.85 | 19.48 |
| IP 21626 | 1.320001 | 19.85 |
| IP 21621 | 1.169999 | 19.95 |
| IP 22008 | 1.22 | 19.97 |
| IP 21622 | 1.22 | 19.97 |
| IP 21620 | 2.289999 | 19.97 |

**Table S2. Archaeological site coordinates.** Sites with grain size information, plotted on map in Figure

| Site | Number | Median Age | Latitude | Longitude |
| --- | --- | --- | --- | --- |
| AZ22 | 1 | -4500 | 20.75 | -0.7 |
| MT25 | 2 | -3500 | 23.72777 | -4.68056 |
| Karkarichinkat Nord (KNO5) | 3 | -2500 | 17.078 | 0.203 |
| MK36 | 4 | -2200 | 20.65 | -3.88333 |
| Winde Koroji | 5 | -2025 | 15.13 | -2.93 |
| Er Negf (EN07) | 6 | -1800 | 17.08776 | 0.302333 |
| Djiganyai | 7 | -1800 | 16.3223 | -7.26936 |
| Ounjougou | 8 | -1725 | 14.4 | -2.48333 |
| Birimi | 9 | -1700 | 10.56483 | -0.39257 |
| Bosumpra cave | 11 | -1500 | 6.697998 | -0.74691 |
| Boase: B5C | 10 | -1665 | 7.852497 | -1.93448 |
| Ti-n-Akof | 12 | -1450 | 14.96388 | -0.14346 |
| Gajiganna | 13 | -1150 | 12.26081 | 13.28911 |
| Kursakata | 14 | -800 | 12.32725 | 14.20201 |
| Walalde | 15 | -675 | 16.49036 | -14.1761 |
| Oued Bou Khzama | 16 | -500 | 16.75245 | -7.27575 |
| Bwambe-Sommet | 17 | -425 | 2.89353 | 9.90334 |
| Oursi West | 18 | -300 | 14.6616 | -0.4986 |
| Abang Minkoo | 19 | -260 | 2.3294 | 11.42325 |
| Old Jarma | 20 | -100 | 26.54425 | 13.06249 |
| Cubalel | 21 | 400 | 16.26571 | -13.9878 |
| Qasr Ibrim | 22 | 450 | 22.64943 | 31.99301 |
| Arondo | 23 | 500 | 14.89998 | -12.4797 |
| Tongo Maare Diabal | 24 | 625 | 15.04532 | -2.94019 |
| Oursi North | 25 | 750 | 14.68414 | -0.46247 |
| Saouga | 27 | 1050 | 14.37294 | -0.14049 |
| Toguere Doupwil | 28 | 1325 | 14.53642 | -4.12100 |
| Dhar Oualata | 29 | -1800 | 17.25096 | -7.09779 |
| Dhar Tichitt | 30 | -1800 | 18.37374 | -9.40328 |
| Oued Chebbi | 31 | -1800 | 17.3 | -7.025 |
| Ti-N-Torha/Two Caves | 32 | -5000 | 25.55058 | 10.54604 |
| Uan Muhuggiag | 33 | -6500 | 24.84266 | 10.51367 |
| Uan Tabu | 34 | -6500 | 24.83806 | 10.48406 |
| Mege | 35 | -625 | 12.22061 | 14.18262 |
| Kasala | 36 | -1800 | 15.17029 | 36.43536 |

**Supplementary Figure S3.** A scatter-plot of average grain width in archaeological assemblages against latitude. Approximate latitude range of wild distribution indicated in yellow highlight. Average grain width of all assemblages indicated by vertical dashed line. Least squares regression shows week correlation between larger grain size and more southerly distribution.

**
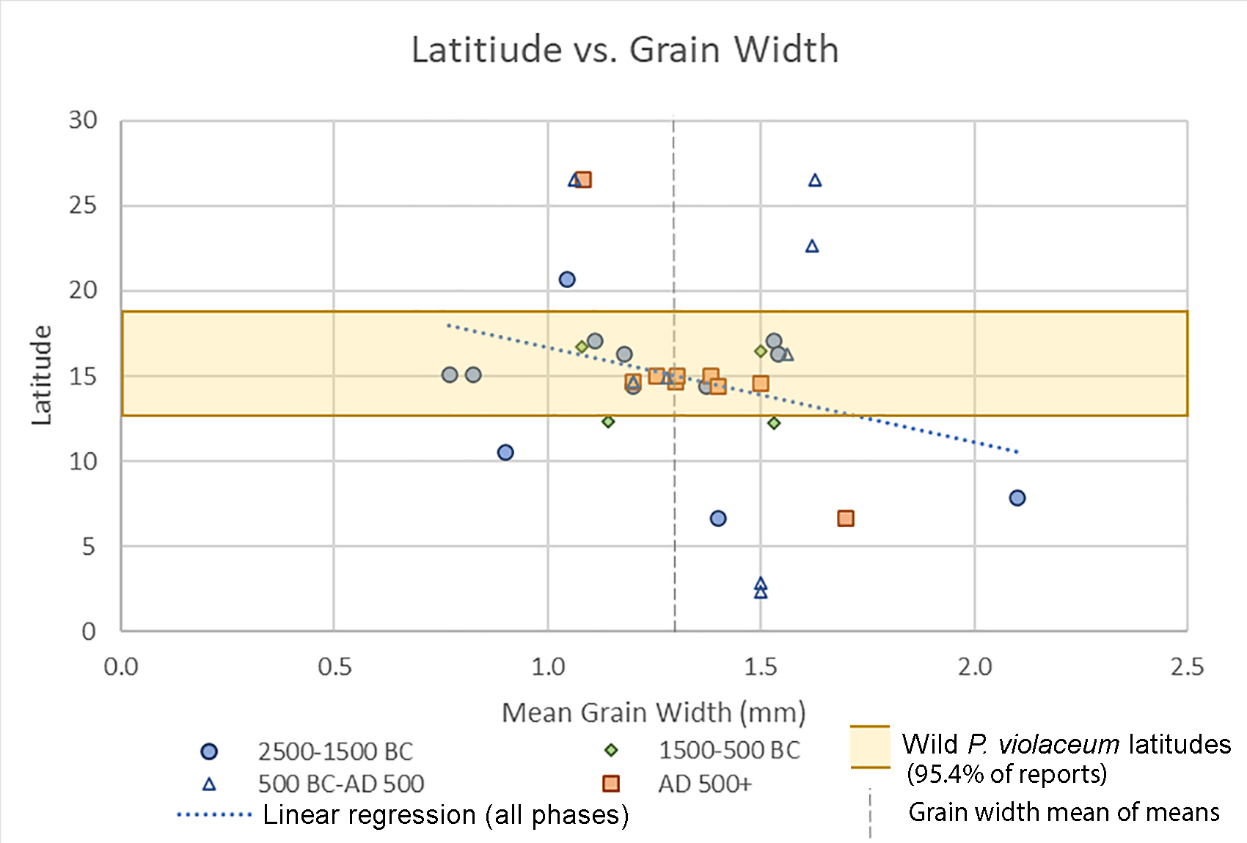
**
