# Supplementary material for: Transition From Wild to Domesticated Pearl Millet (Pennisetum glaucum) Revealed in Ceramic Temper at Three Middle Holocene Sites in Northern Mali
Source: Afr Archaeol Rev. 2021 Mar 16;38(2):211–30. doi: 10.1007/s10437-021-09428-8 (PMC8550313; doi:10.1007/s10437-021-09428-8)

Supplementary material for **Transition from wild to domesticated Pearl Millet (*Pennisetum glaucum)* revealed in ceramics tempers from three Middle Holocene sites in Northern Mali**

This supplement includes images of each sherd subjected to micro CTscanning and examples of extracted plants remains from the resulting high-resolution tomography. A brief typological characterization of each sherd is also provided. All remains are identified as pearl millet (*Pennisetum glaucum/ violaceum*), unless otherwise indicated.

MK36

Sherd 1237

Pivoting impression A+B Fishnet with a curved edged spatula
Impression pivotante A+B Fishnet avec spatule à front courbe (https://lampea.cnrs.fr/cerafim/spip.php?article72)

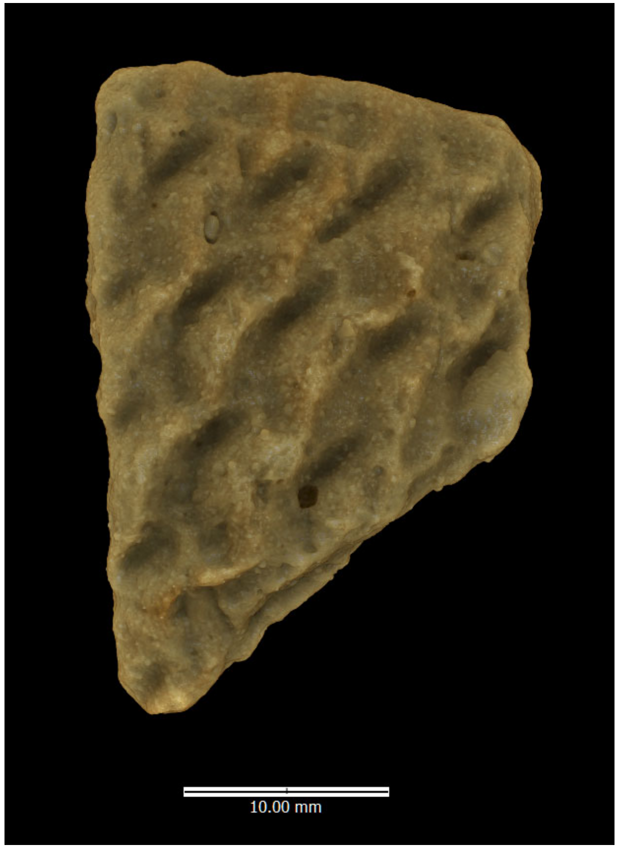

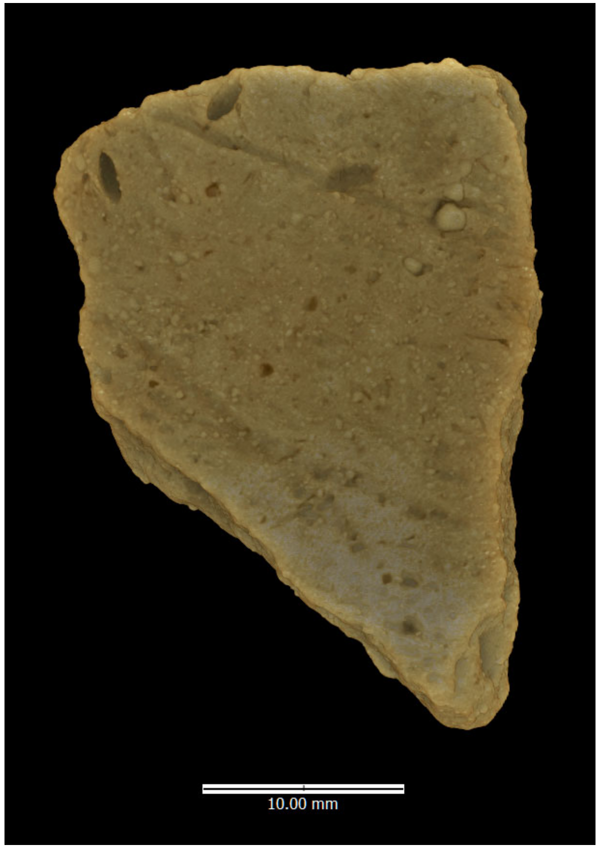

Inclusion 1

Involucre base with torn peduncle

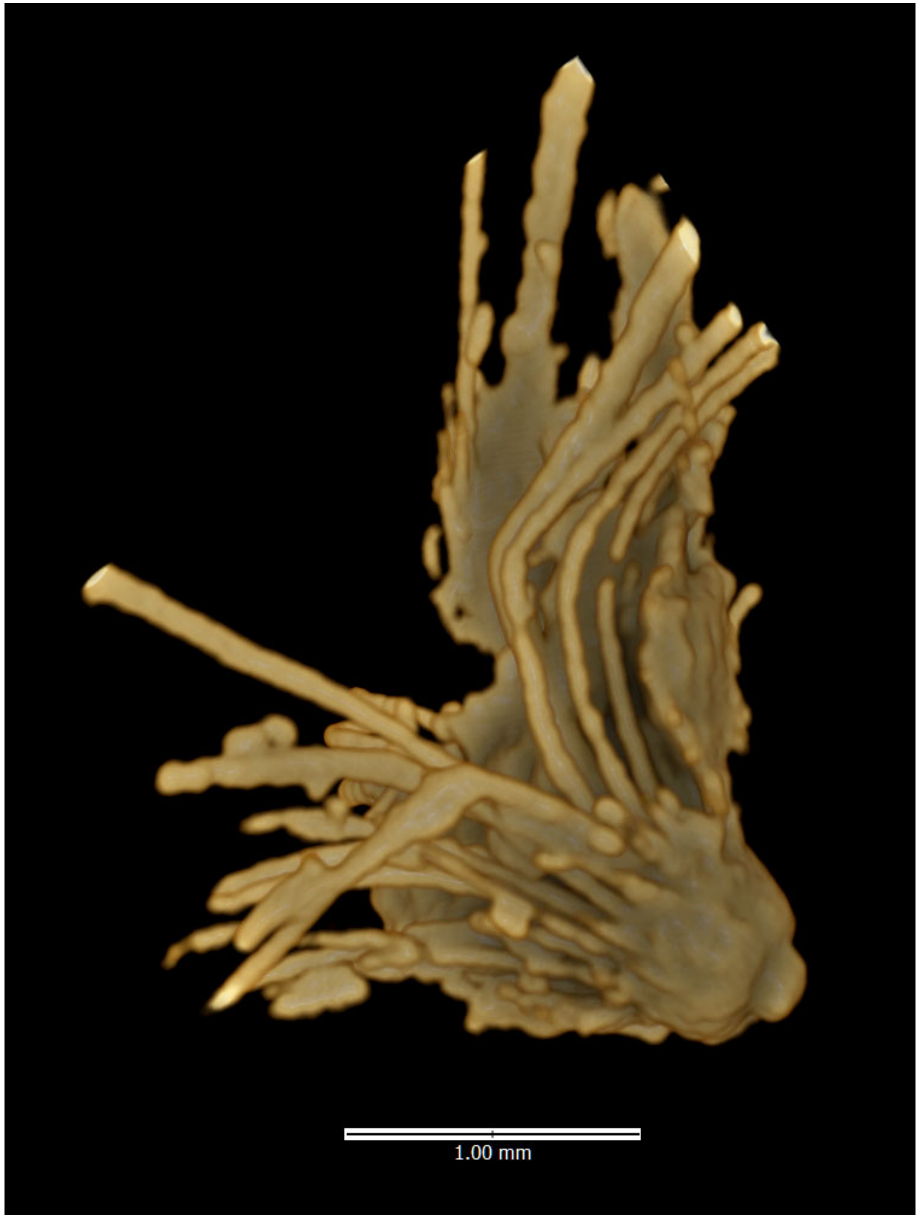

Inclusion 3. Two views of spikelet

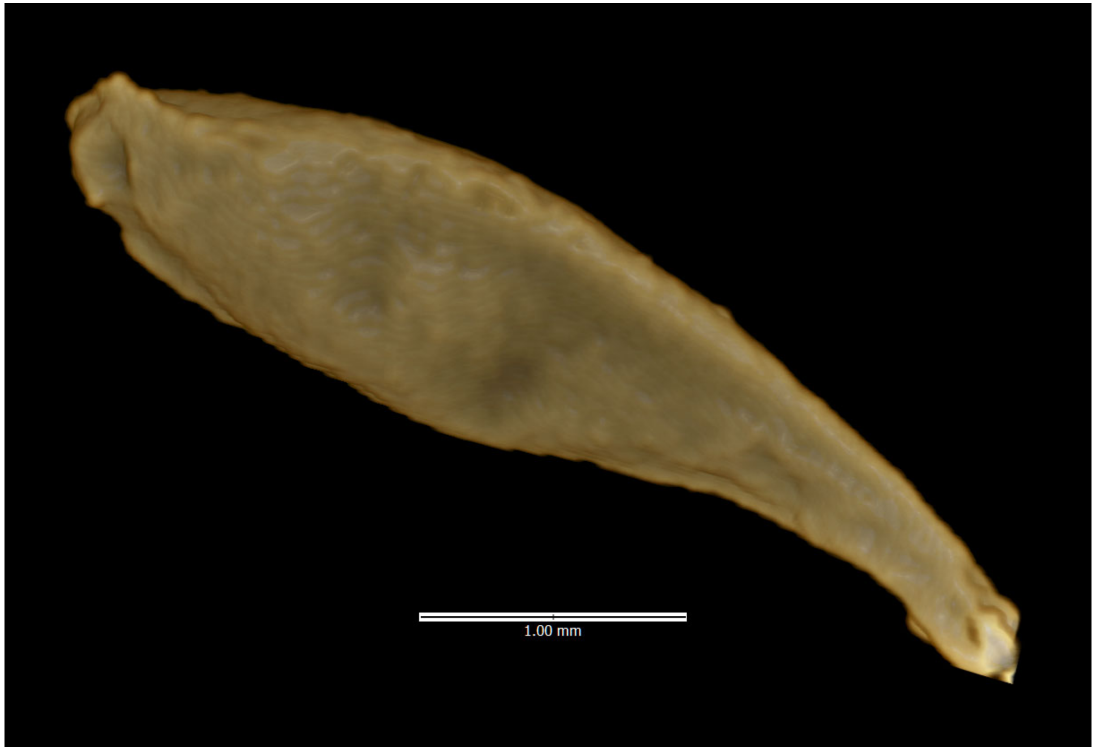

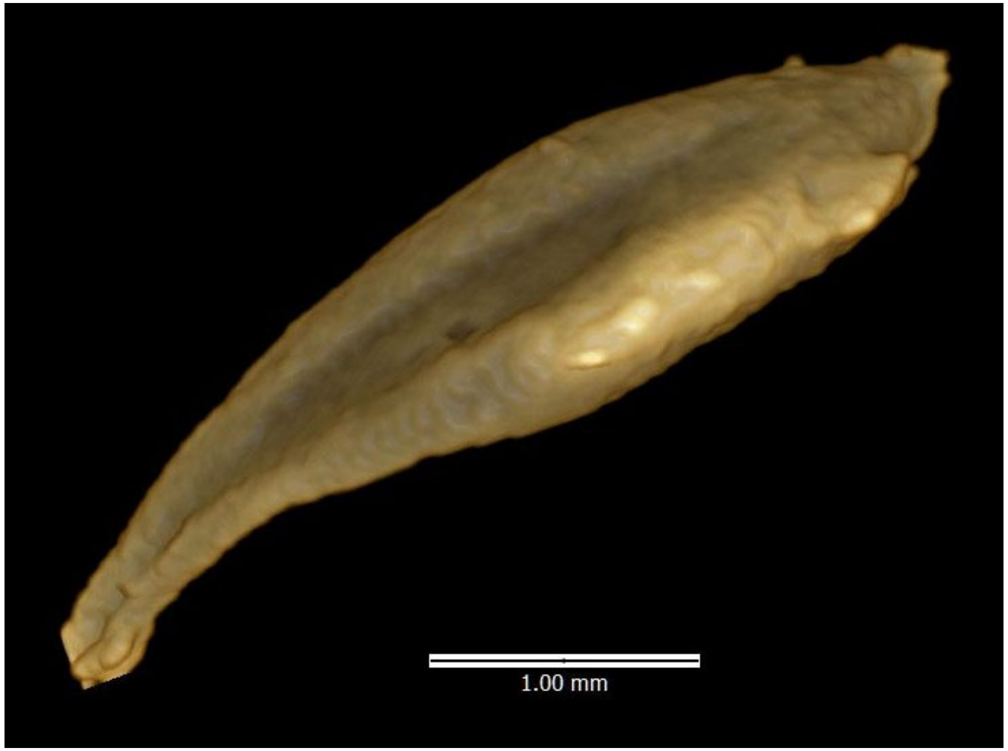

Inclusion 11. Two views of spikelet, from paired spikelets

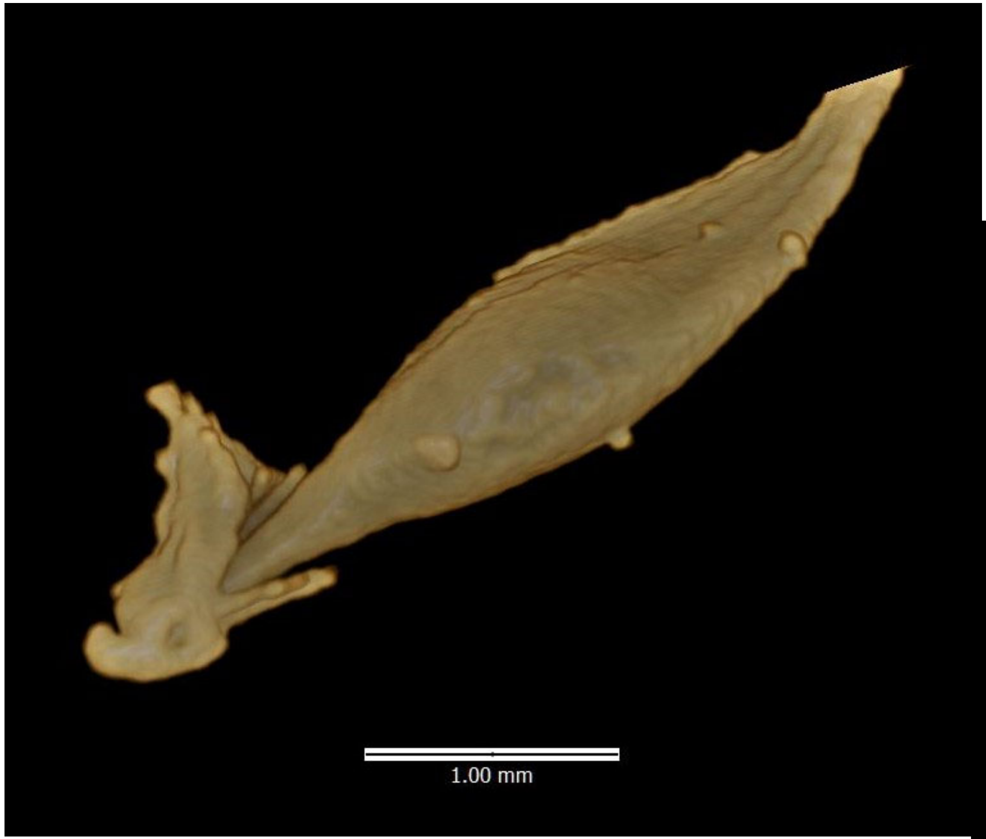

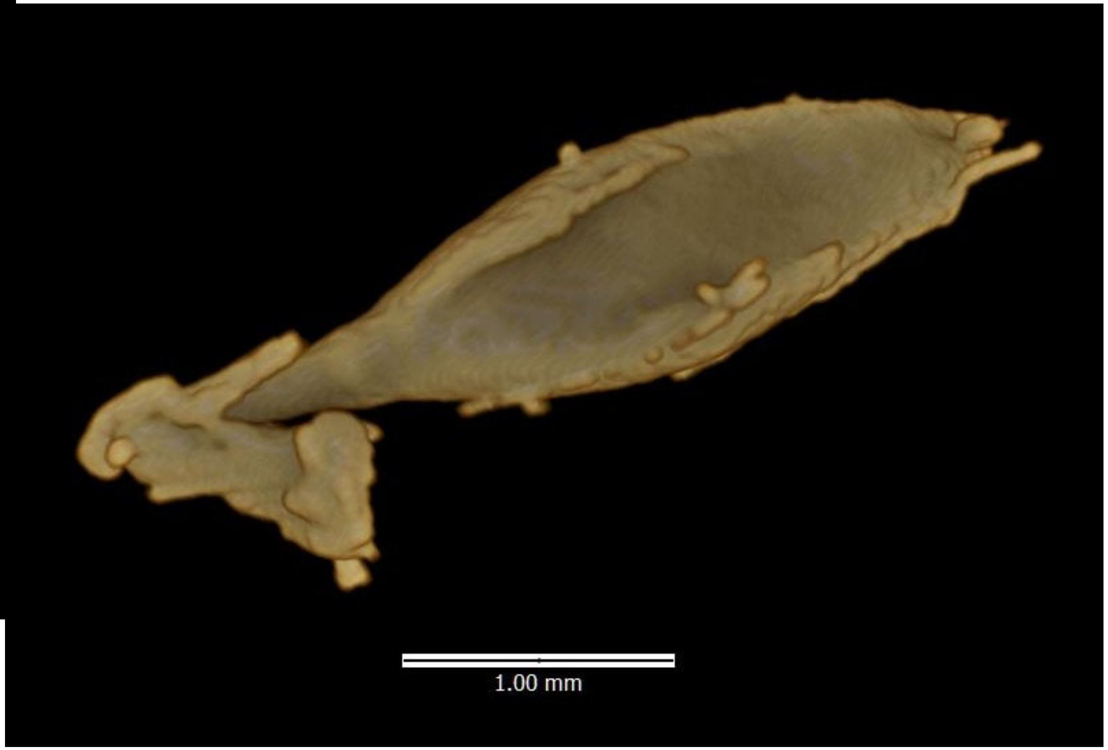

Inclusion 12. Two views, spikelet fragment

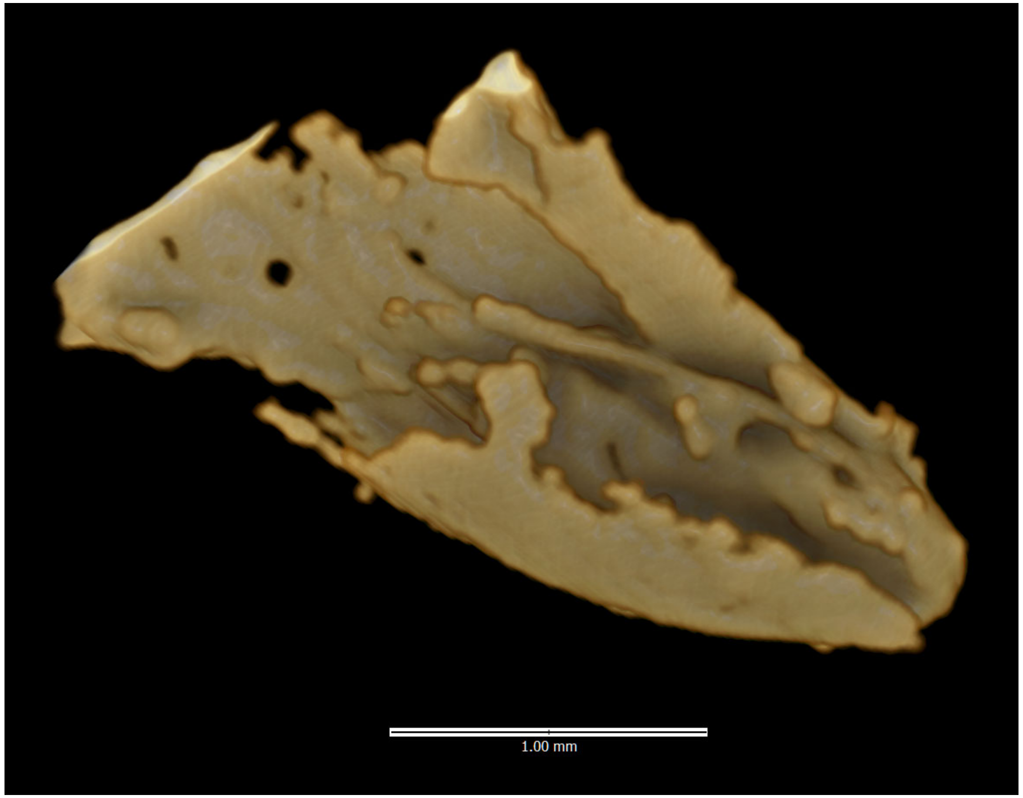

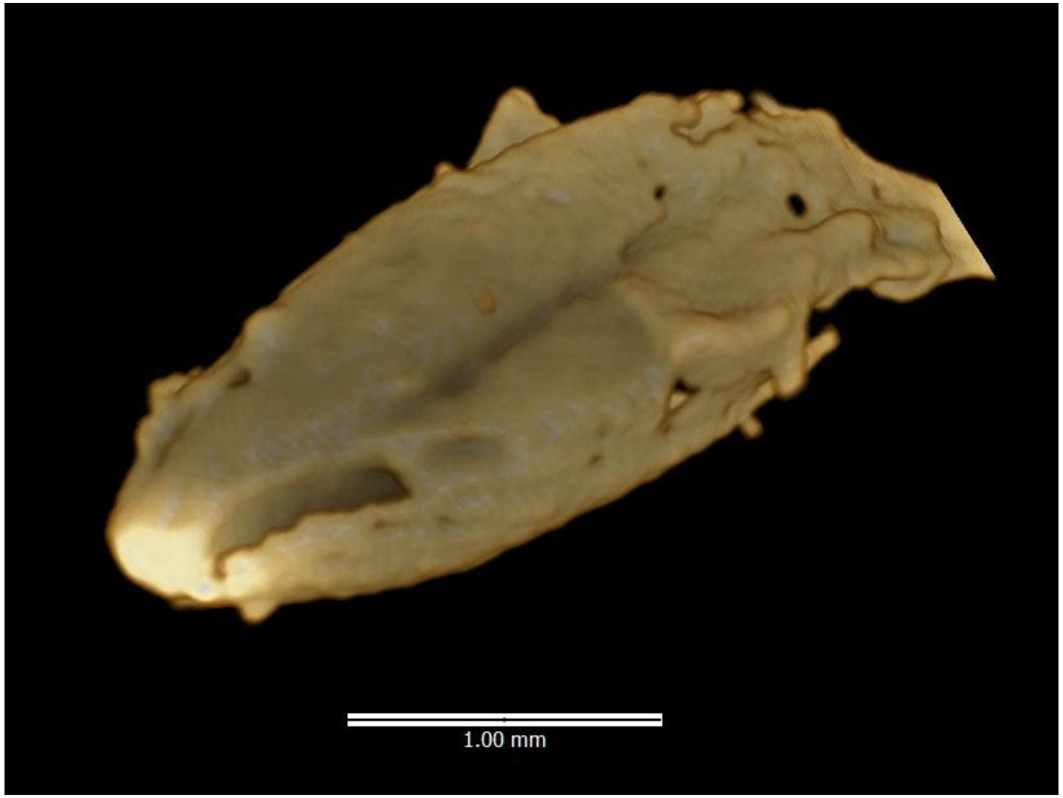

Inclusion 13. Two views of solitary spikelet in fragment of probabl sessile involucre base, with preserved bristles.

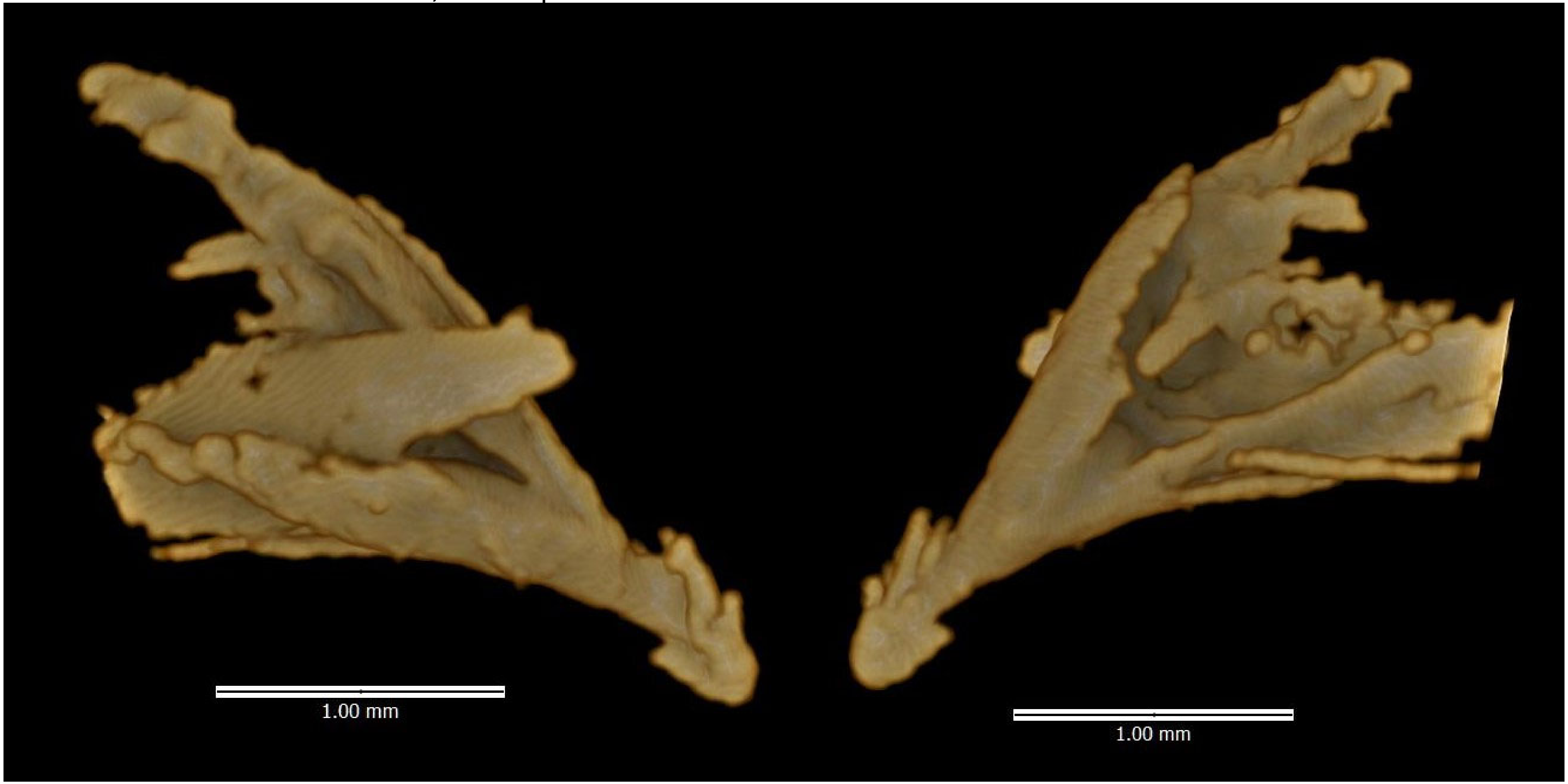

Sherd 295

Eroded surface

Surface érodée,
Décor non identifiable

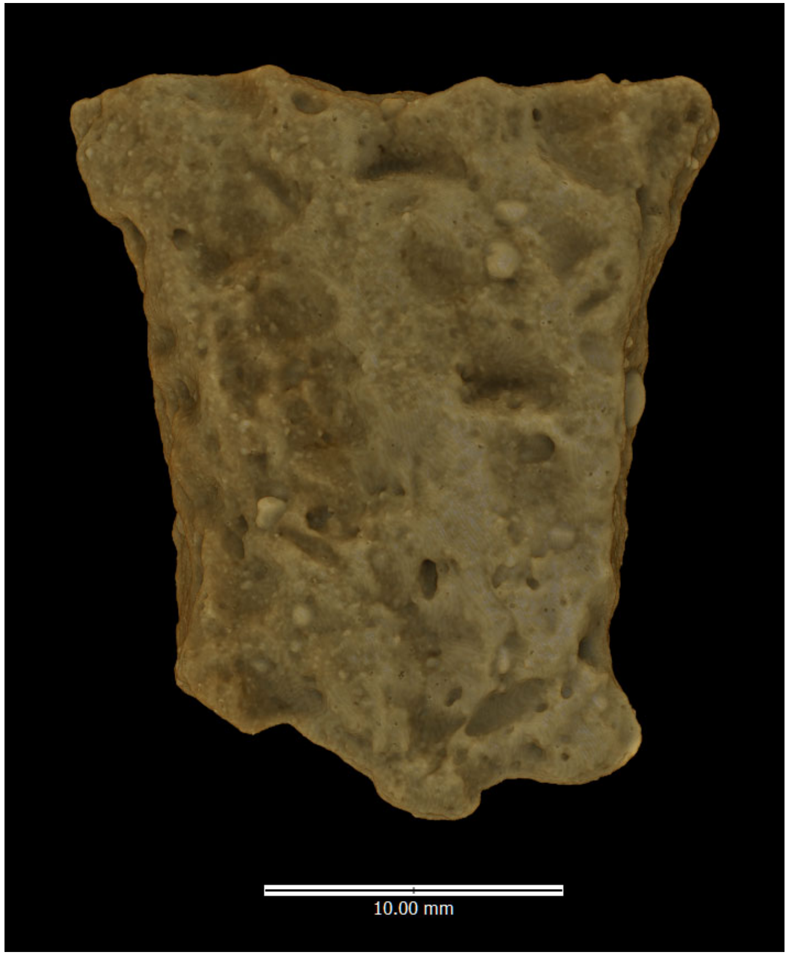

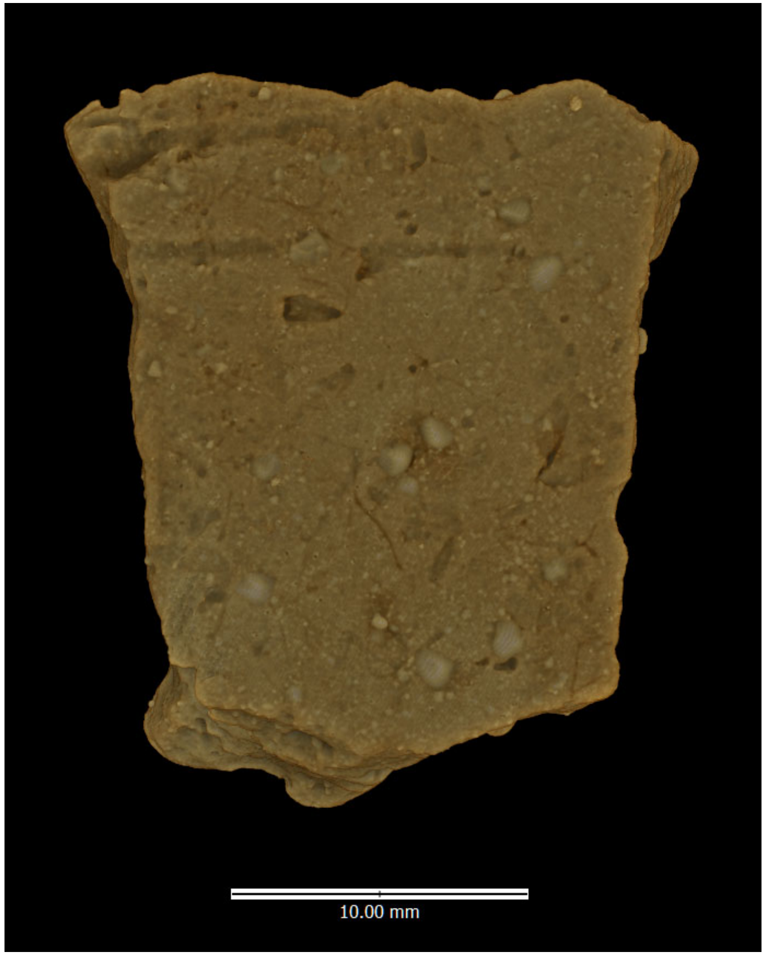

Inclusion 31. Two views, spikelet.

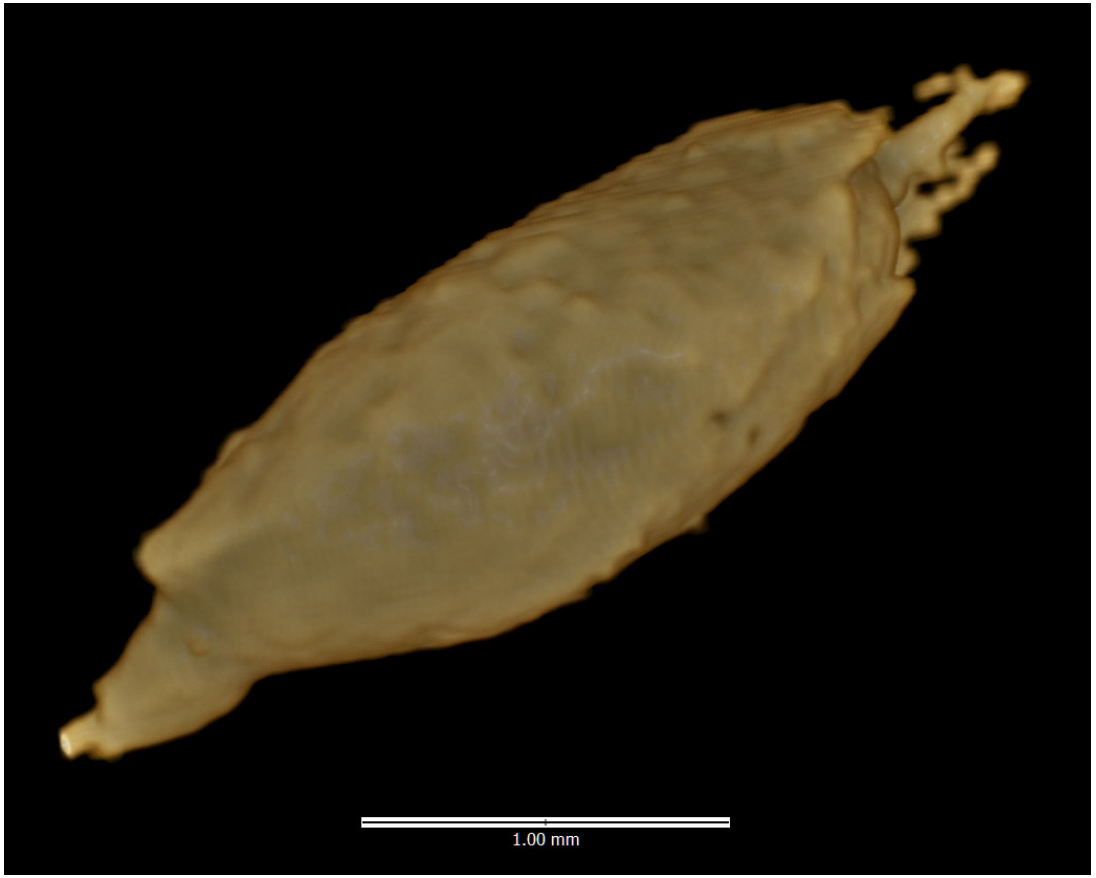

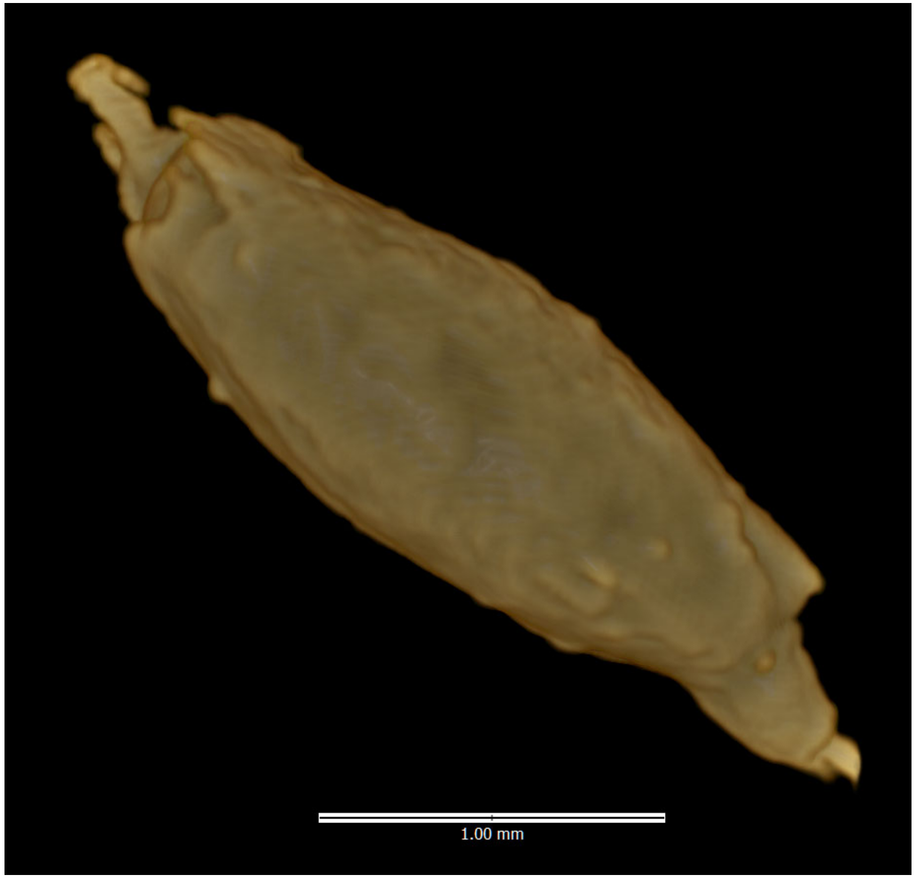

Inclusion 32. Two views, spikelet

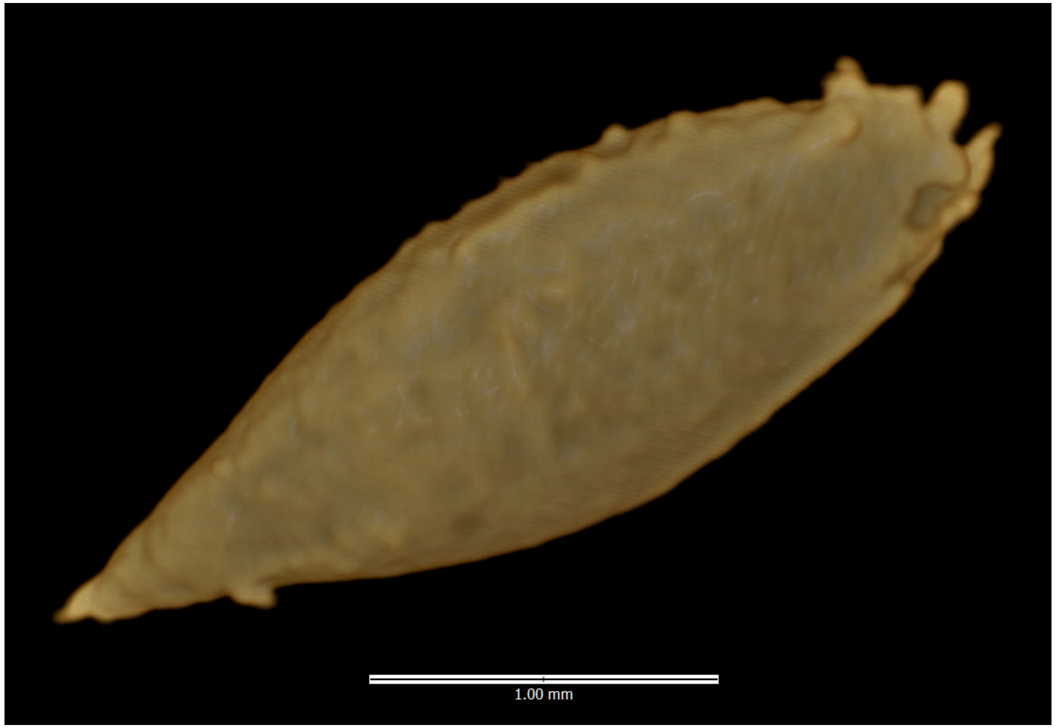

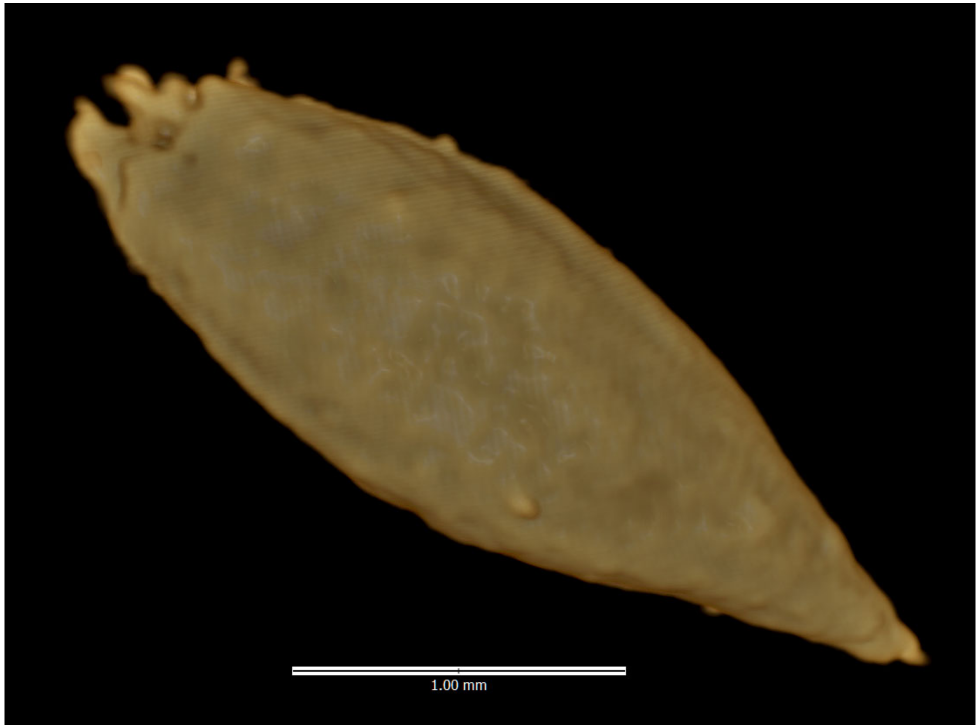

Sherd 304

Eroded surface

Surface érodée,
Décor non identifiable

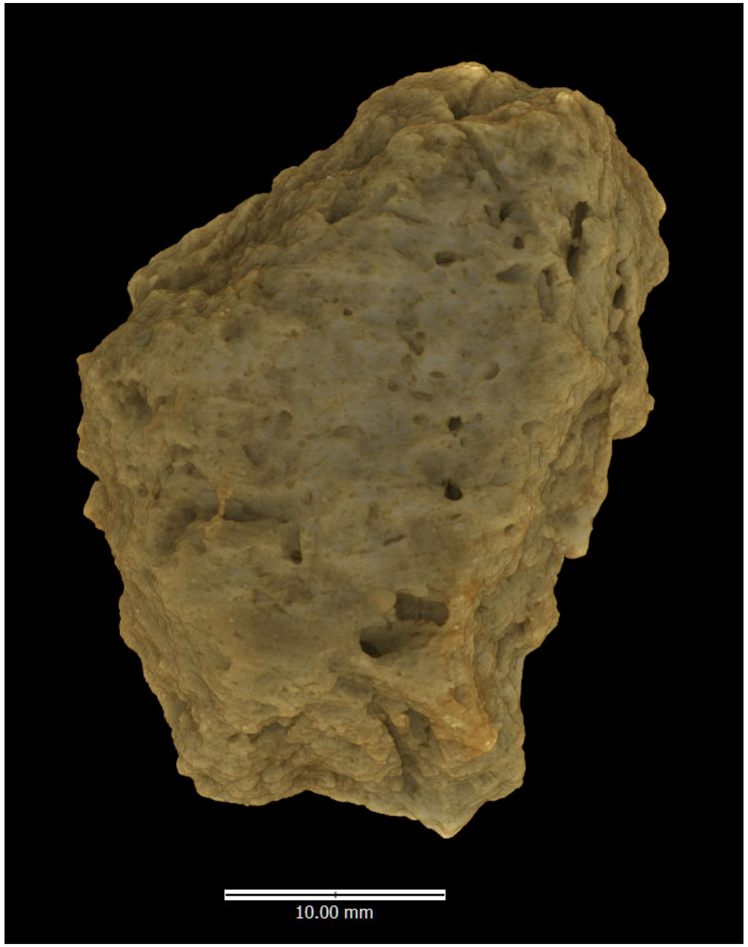

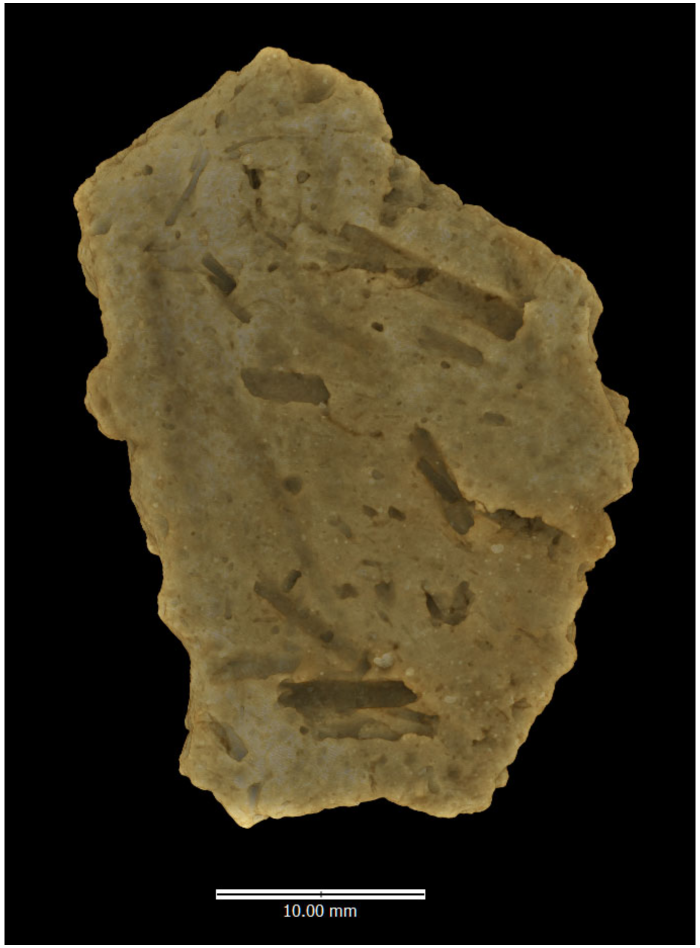

Inclusion 8. Two views, spikelet

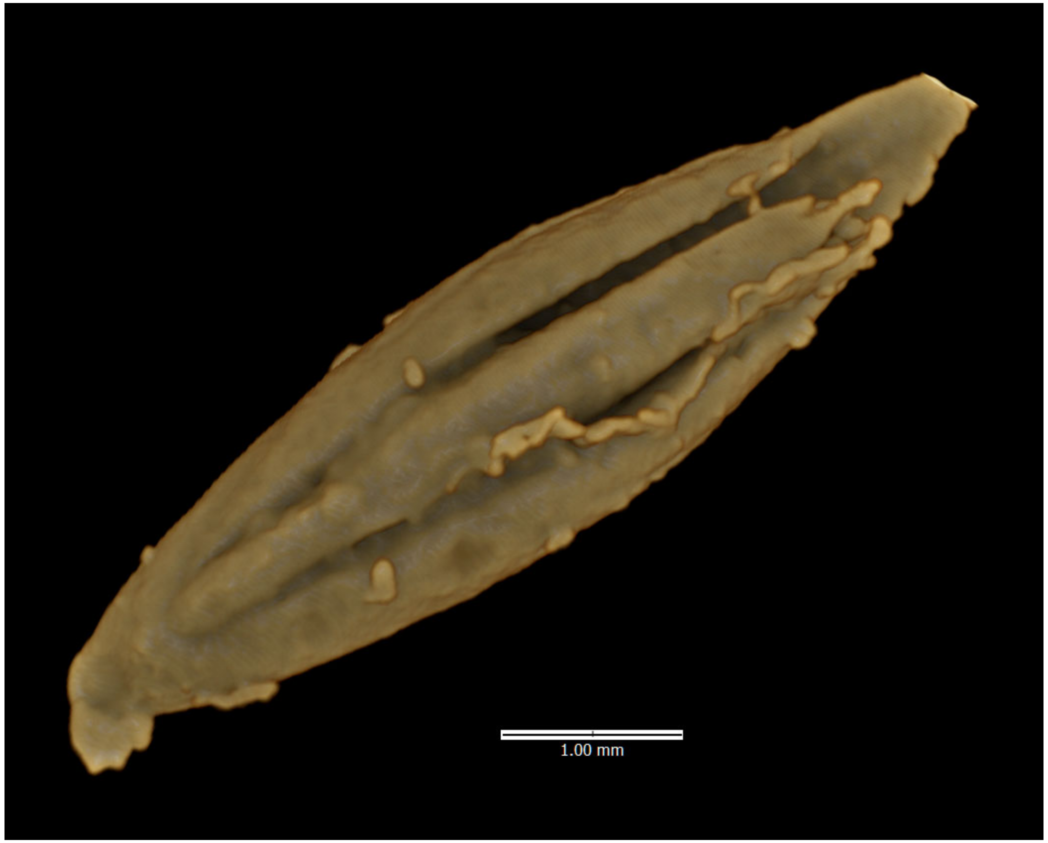

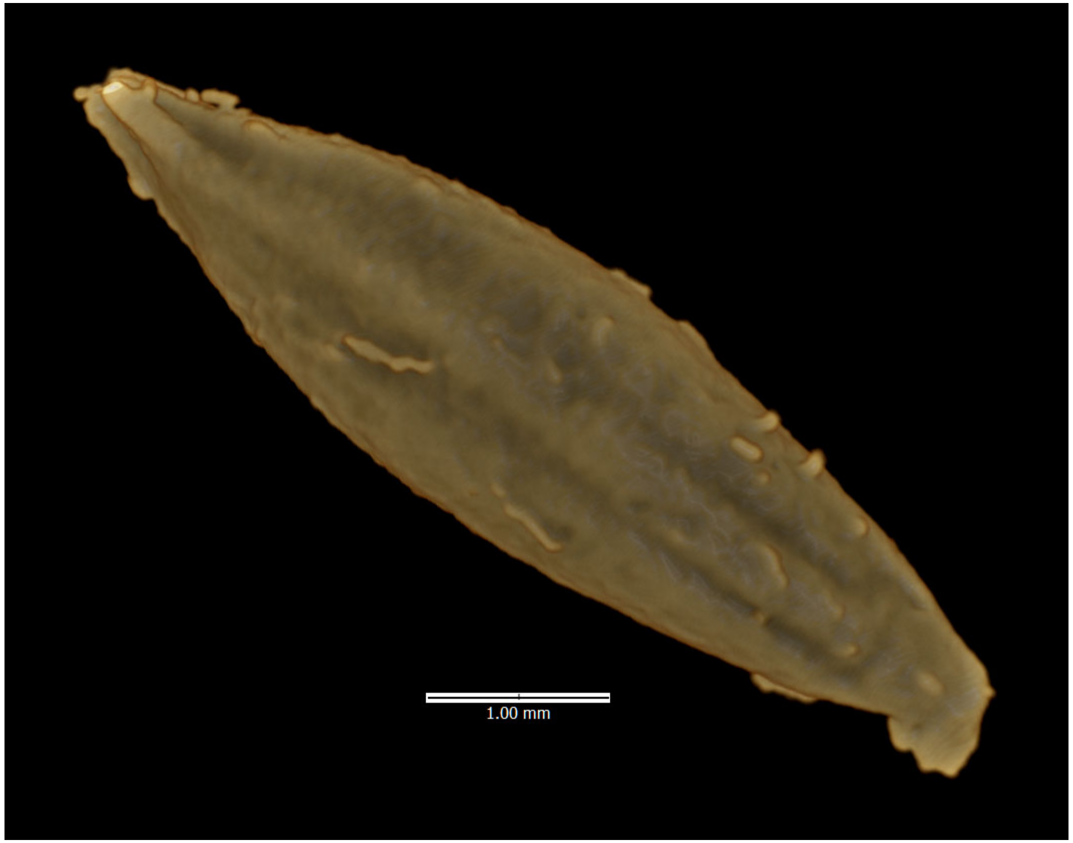

Sherd 369

Eroded surface

Surface érodée,
Décor non identifiable

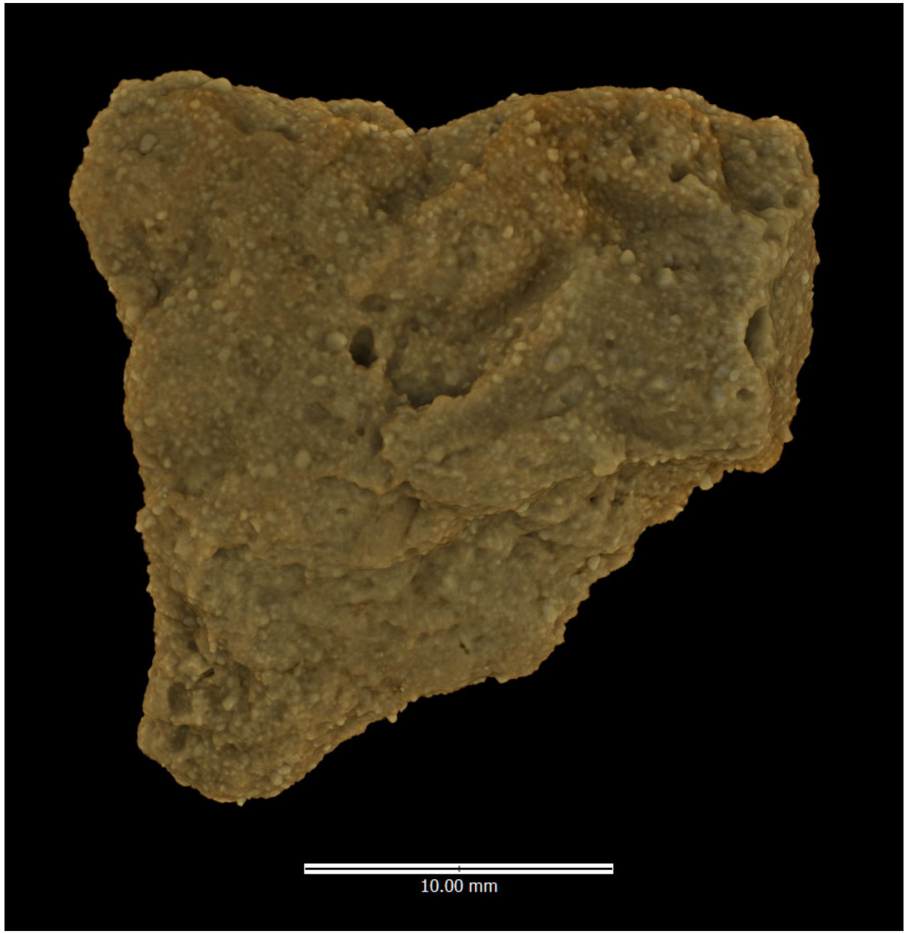

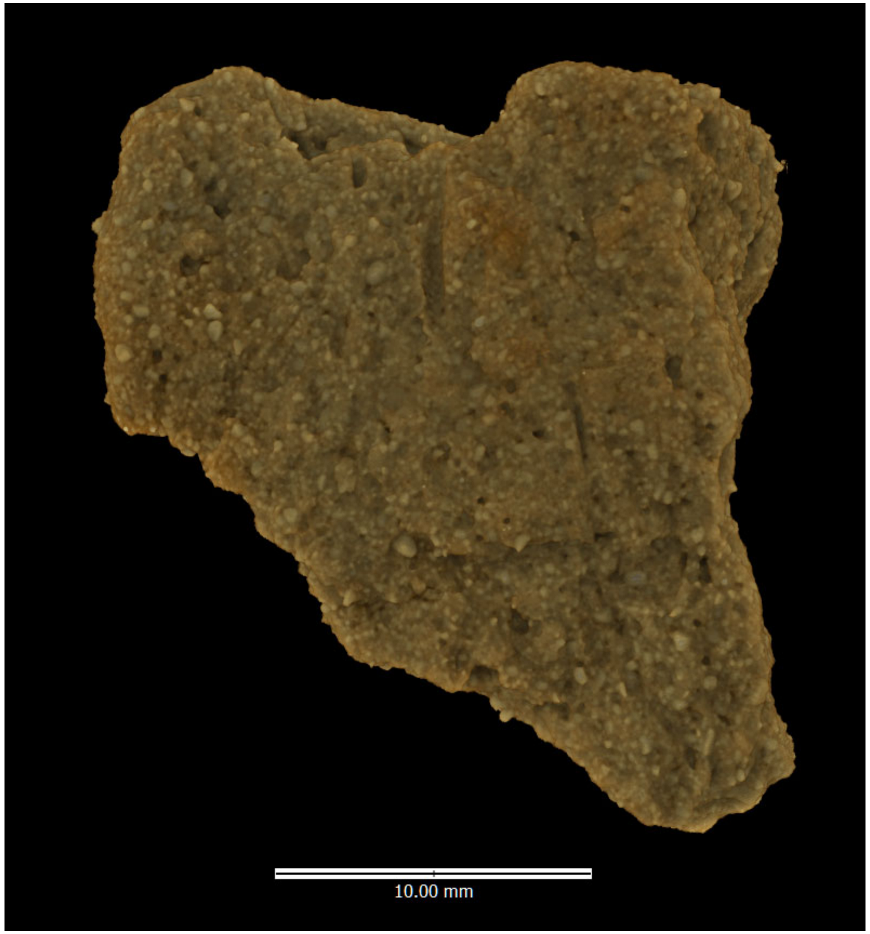

Inclusion 4. Two views, fragmentary spikelet (lemma and palea)

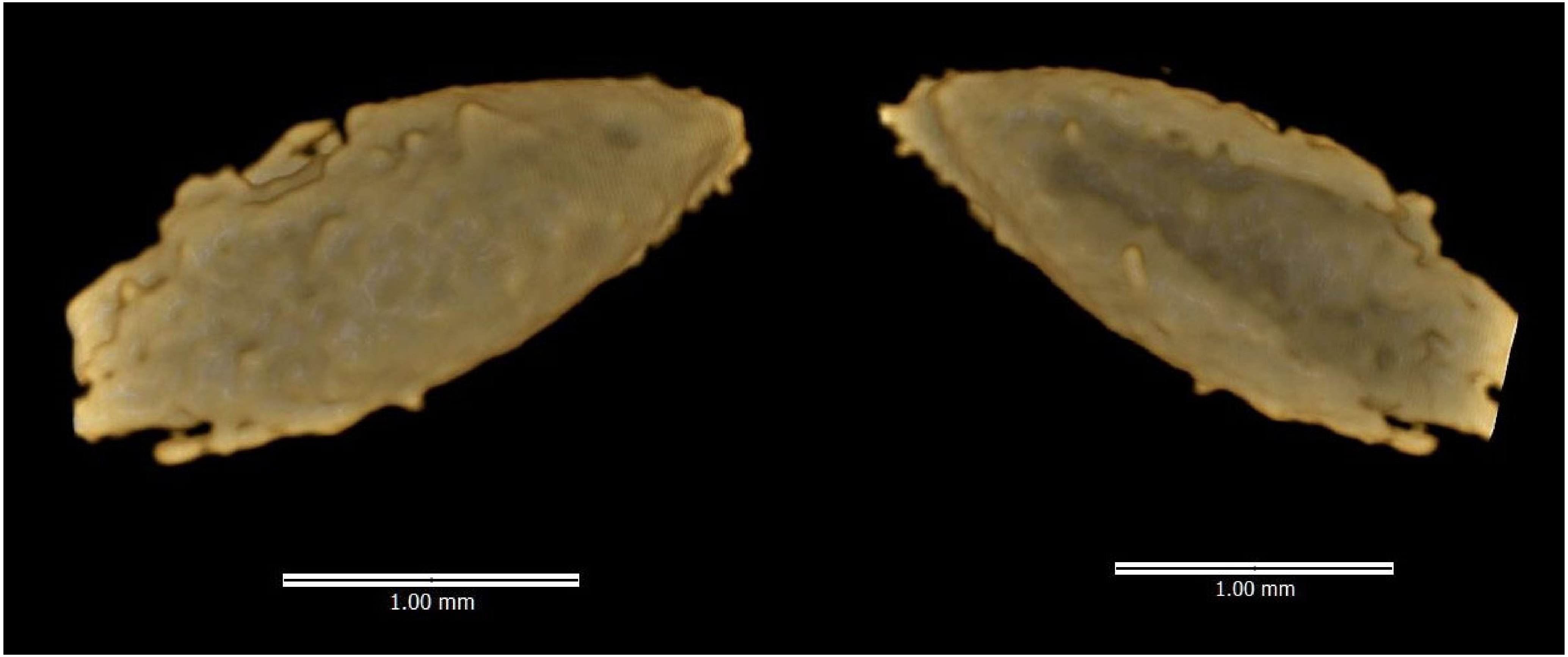

Sherd 728

Decoration:
Perpendicular simple impression with round edged point

Impression simple perpendiculaire avec poinçon à partie active mousse [(https://lampea.cnrs.fr/cerafim/spip.php?article232)](https://lampea.cnrs.fr/cerafim/spip.php?article232)

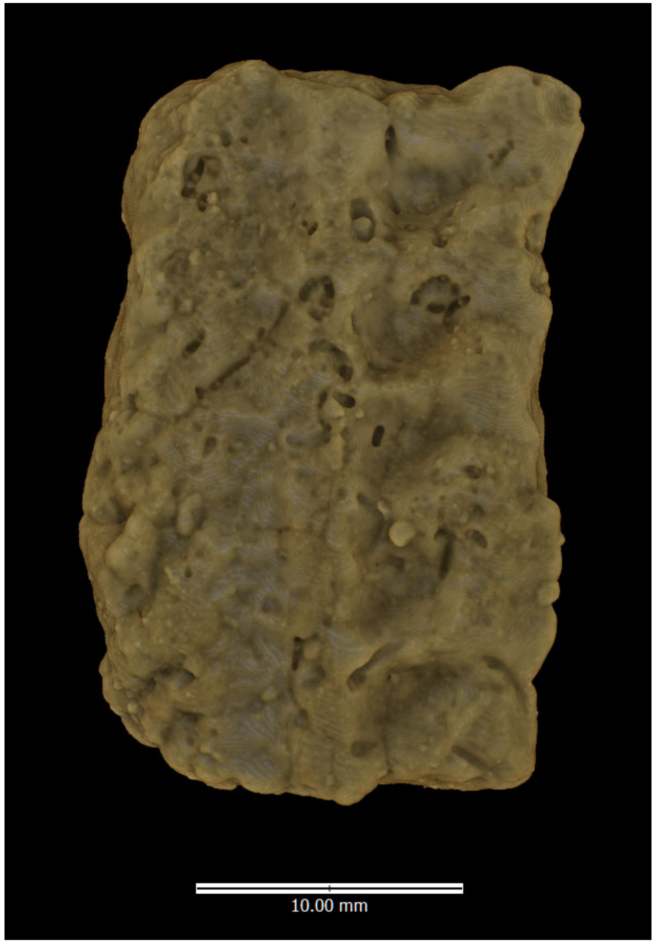

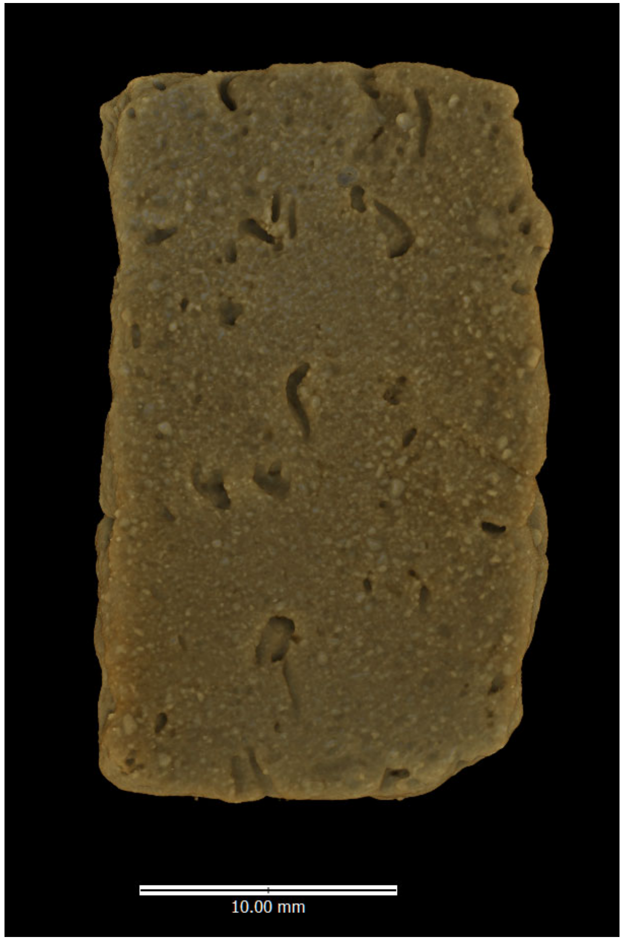

Inclusion 5.

Bristle fragments from involucre base

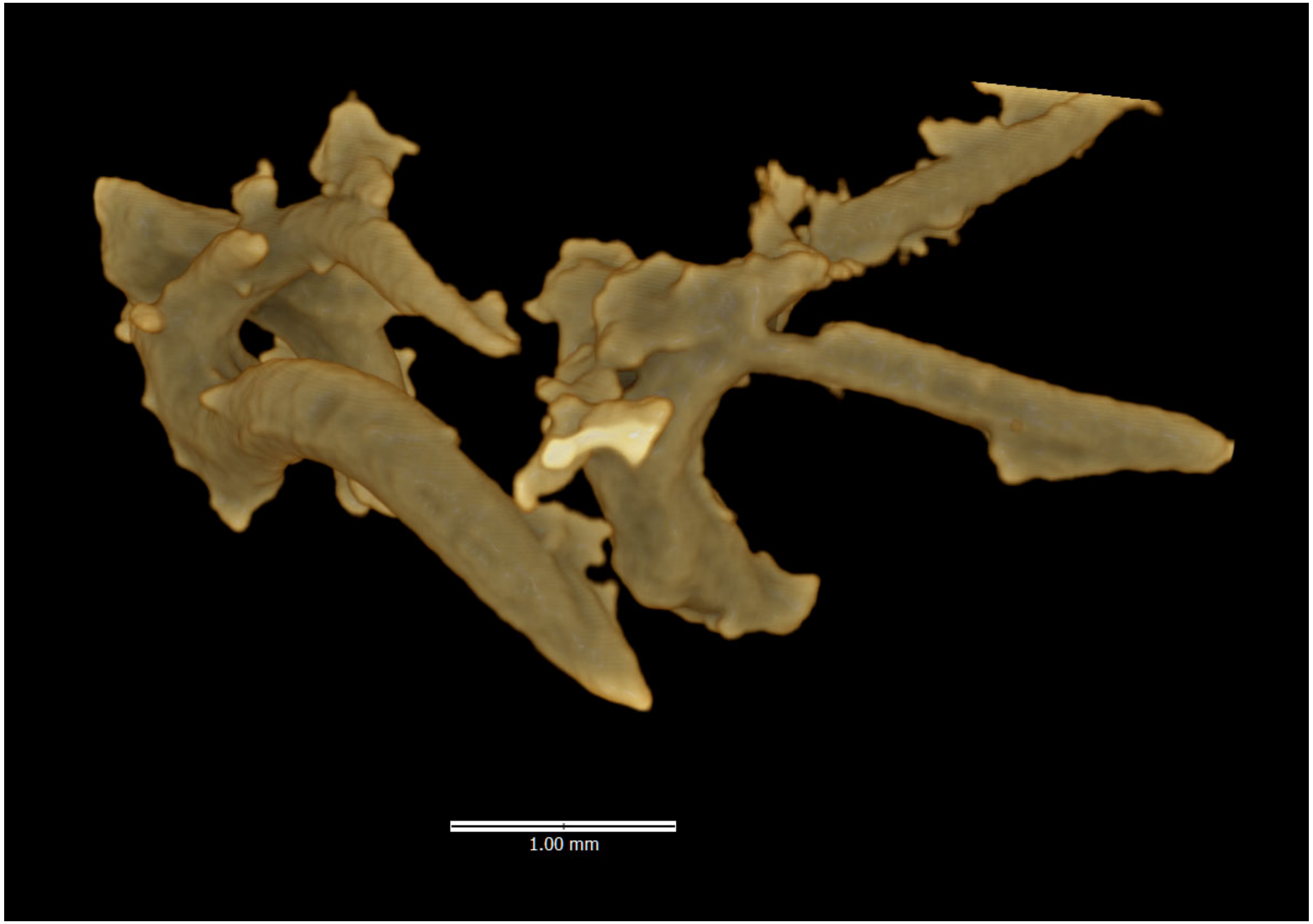

Sherd 543

Decoration:
Pivoting impression A+B Leiterband with a multiple teeth (dots and dashes) comb

Impression pivotante A+B Leiterband avec un peigne à dents multiples mixtes. (https://lampea.cnrs.fr/cerafim/spip.php?article242)

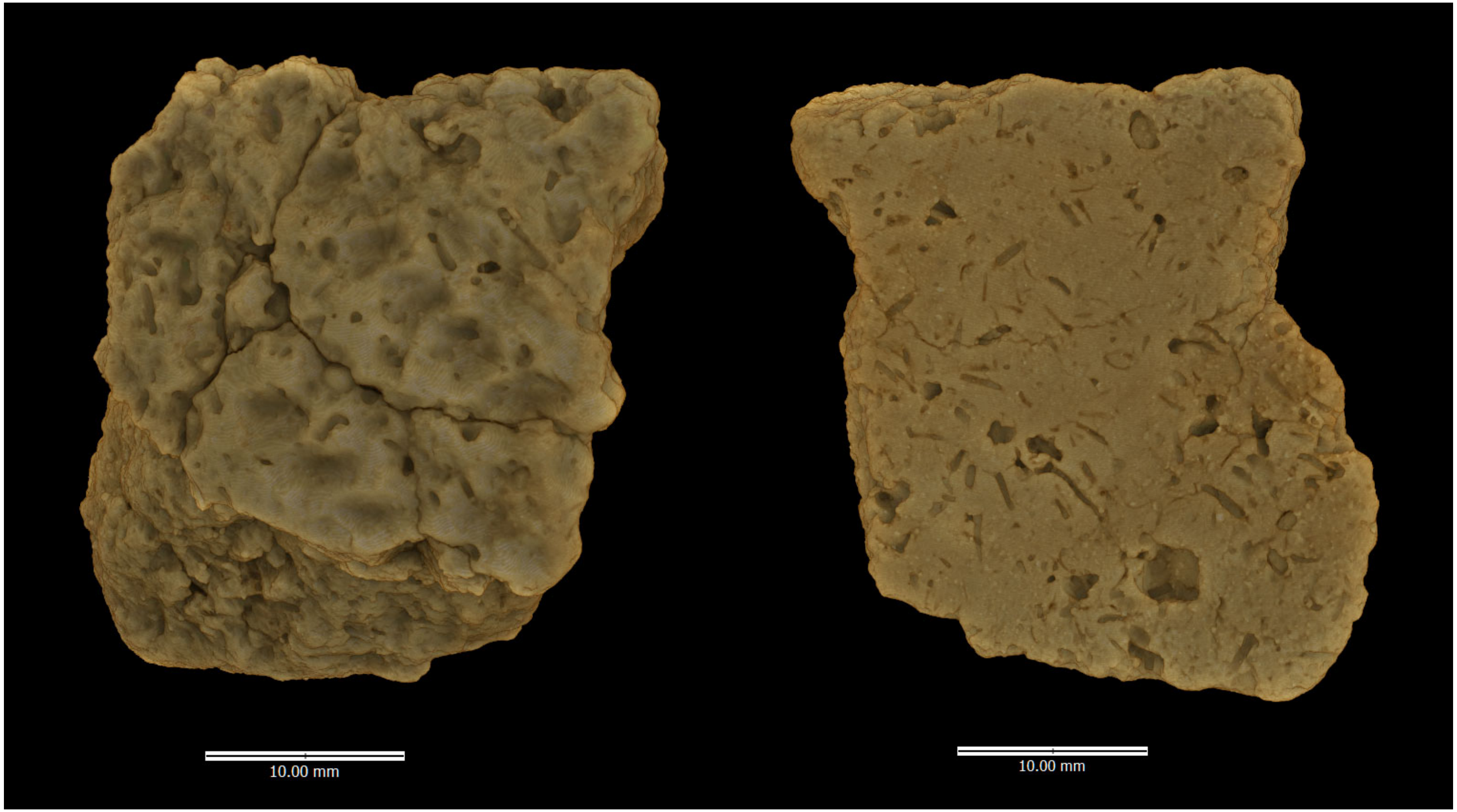

Inclusion 16. *Echinochloa* sp. in husk (spikelet without glumes)

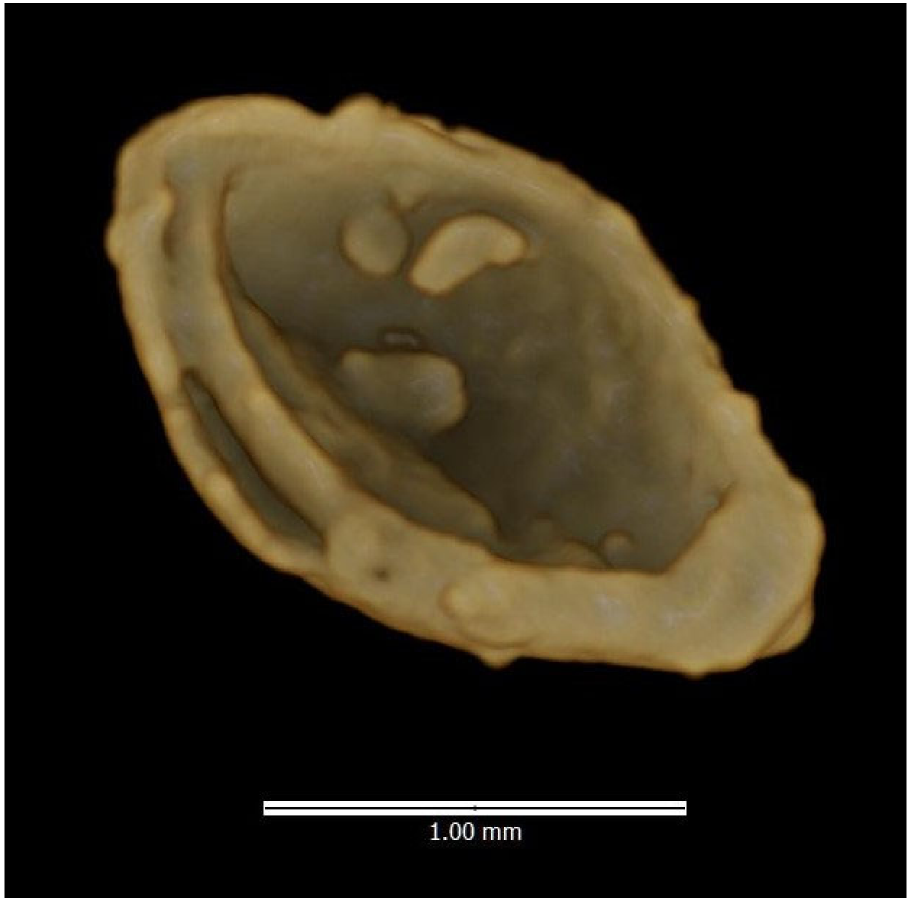

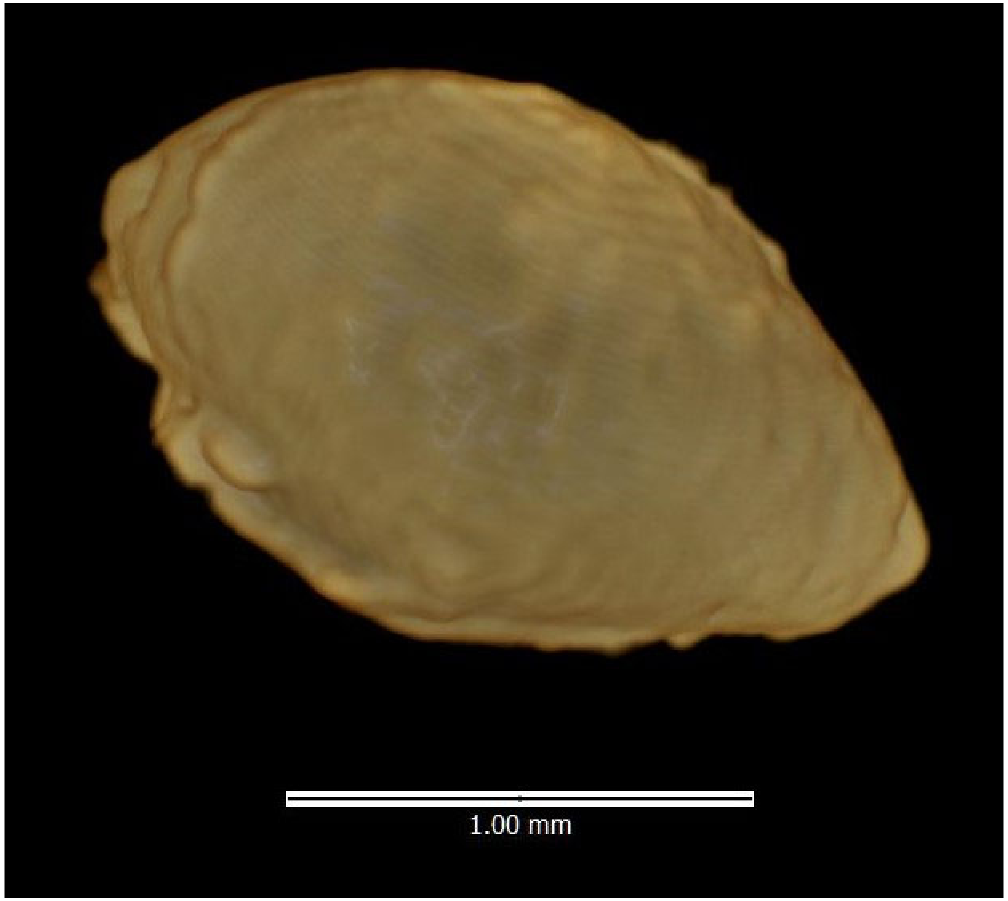

Inclusion 17. Two views, spikelet

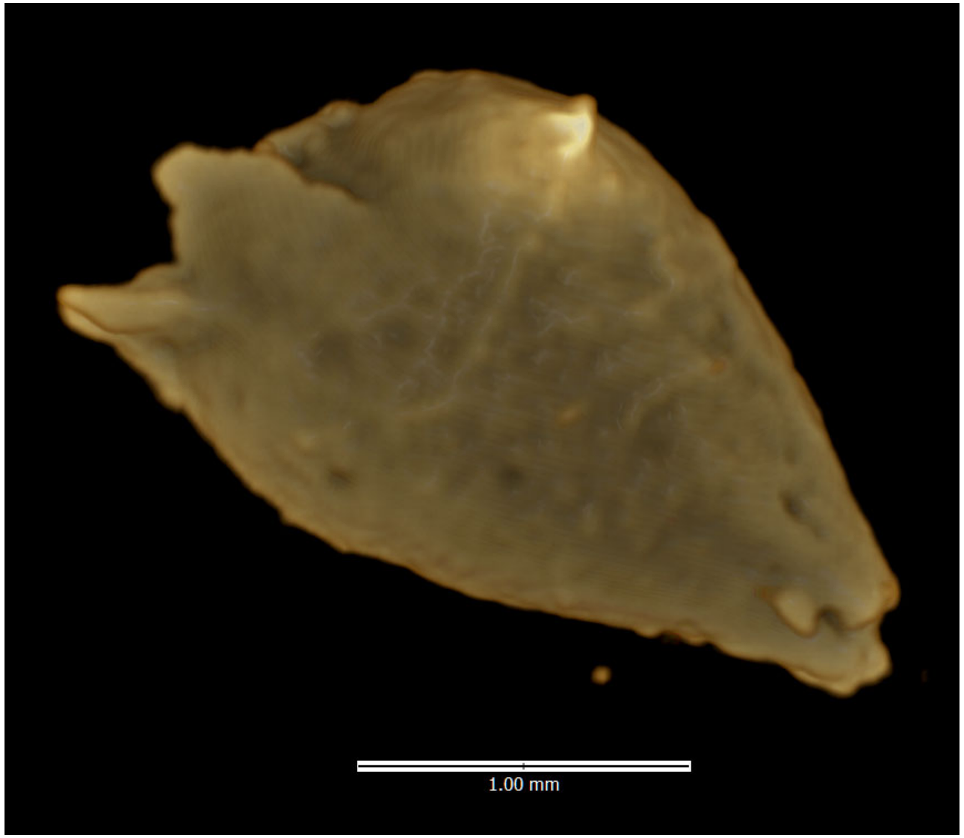

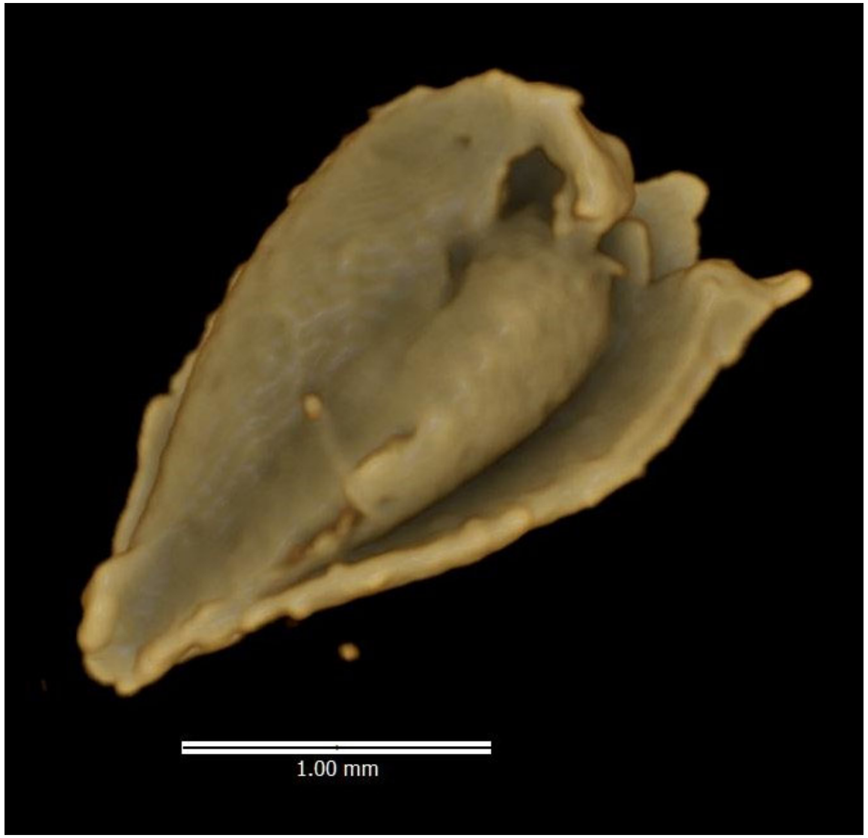

Sherd 276

Eroded surface
Indetermined decor

Surface érodée,
Décor non identifiable

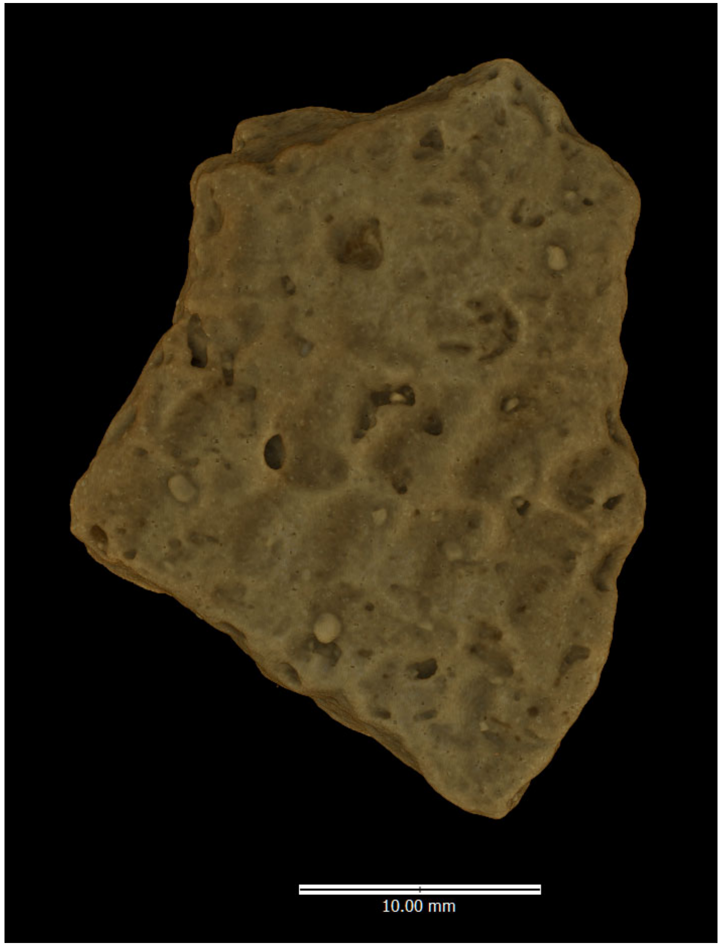

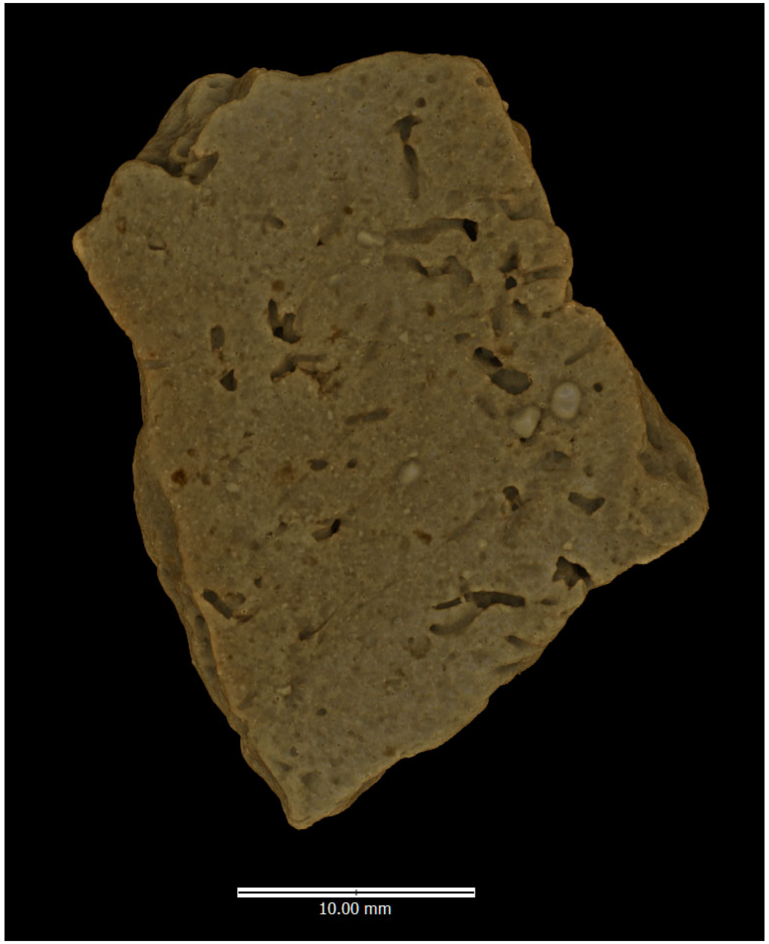

Inclusion 18. Two views of involucre base, with torn peduncle, and bristles.

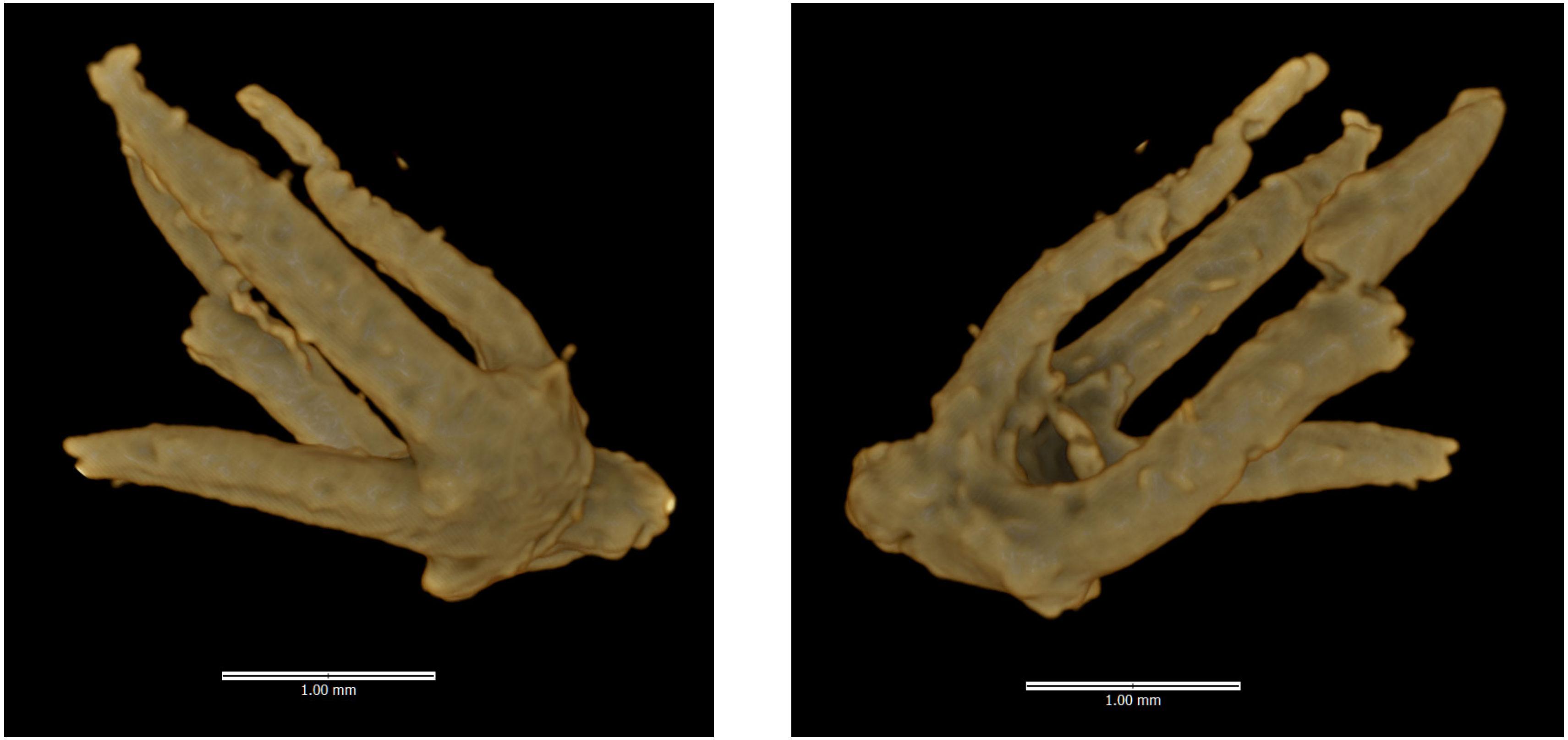

Inclusion 19. Bristle fragments from involucre base

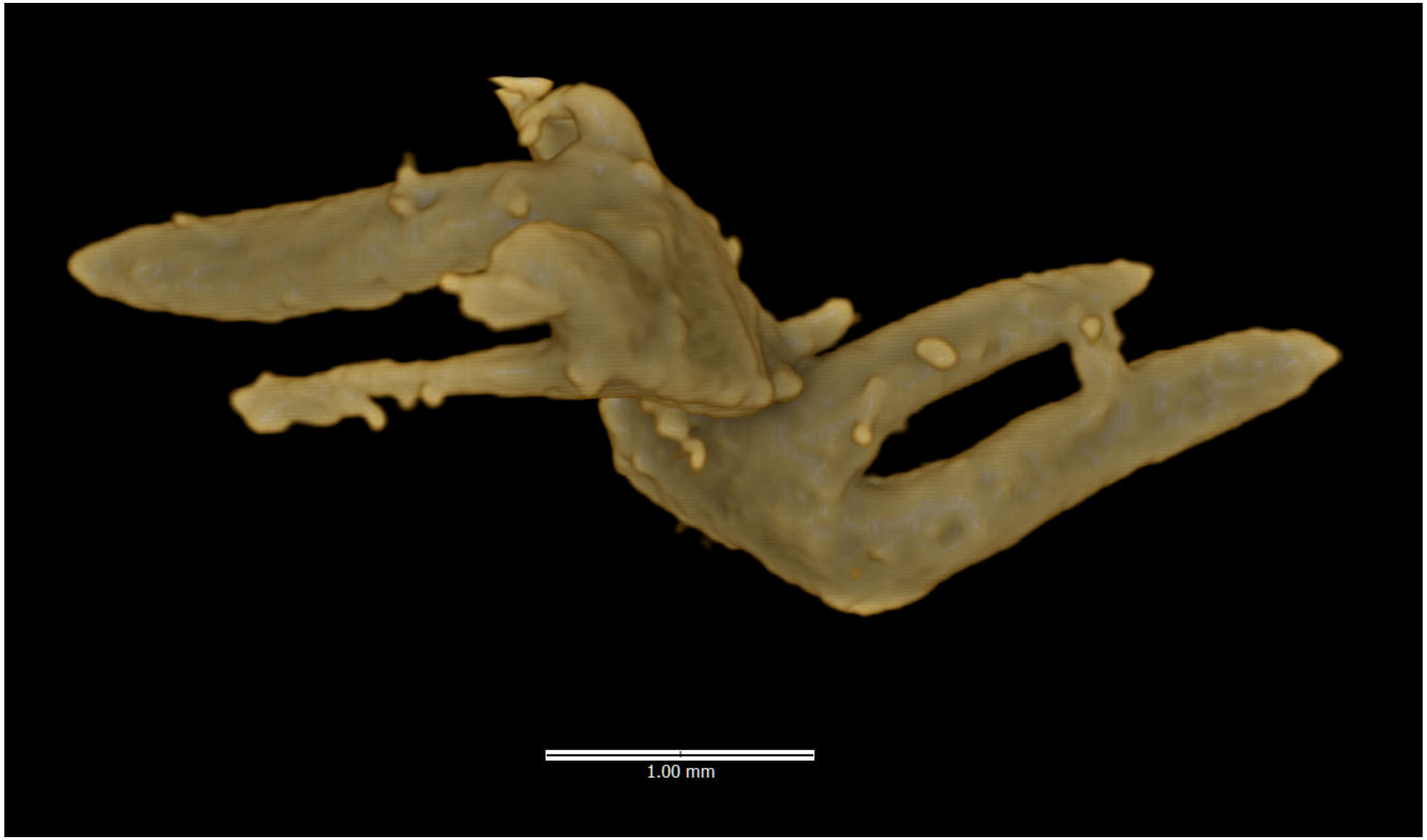

Inclusion 20. Two views: Involucre with sessile base (wild-type abscission) , and paired spikelets.

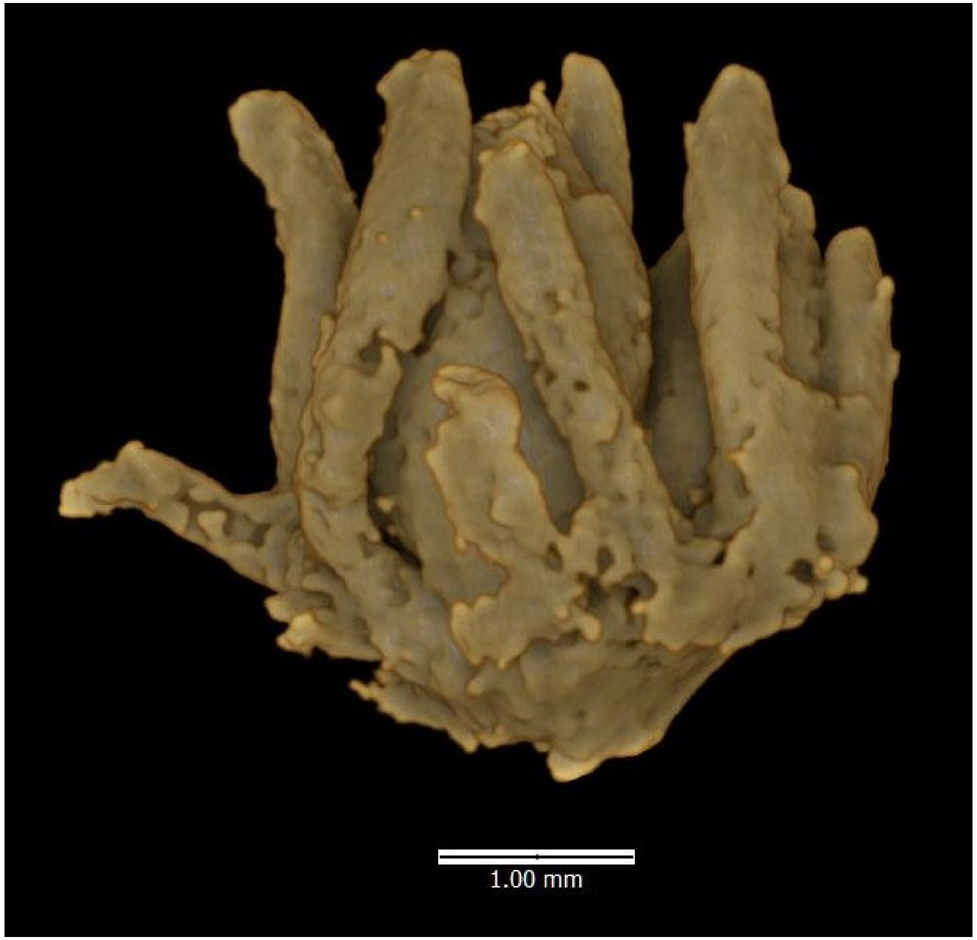

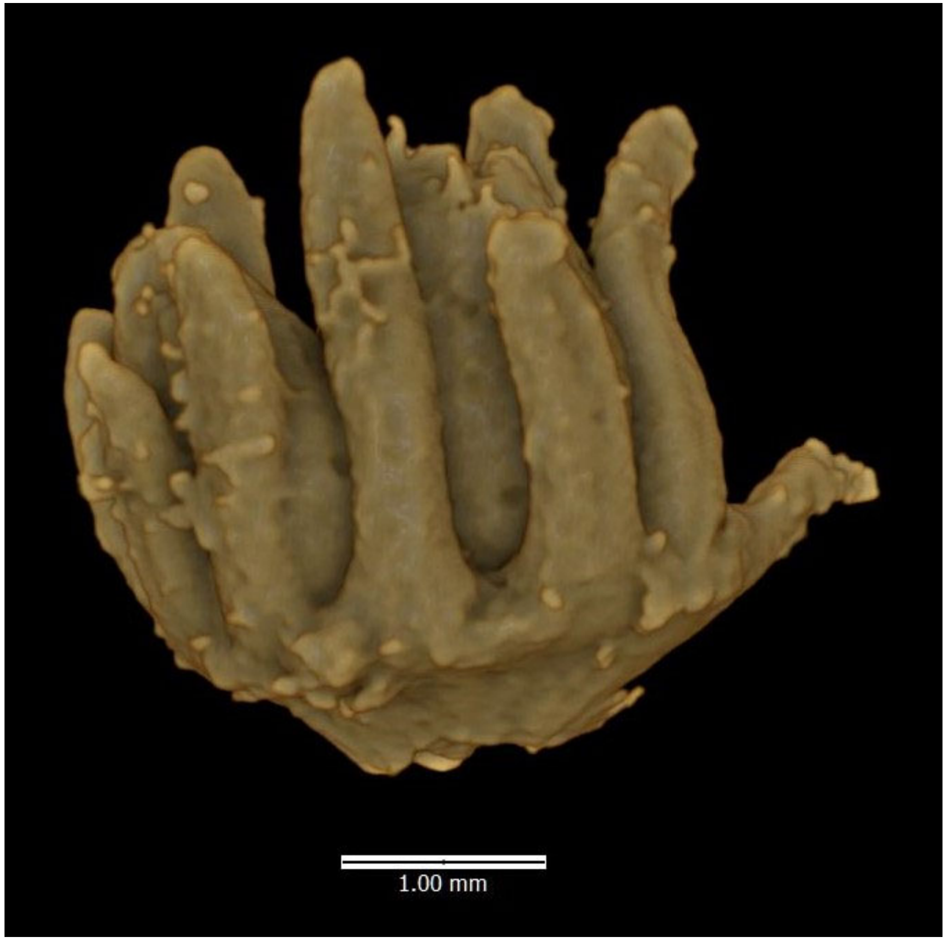

Inclusion 20. Two more views, highlighting sessile base (abscission scar) , and paired spikelets.

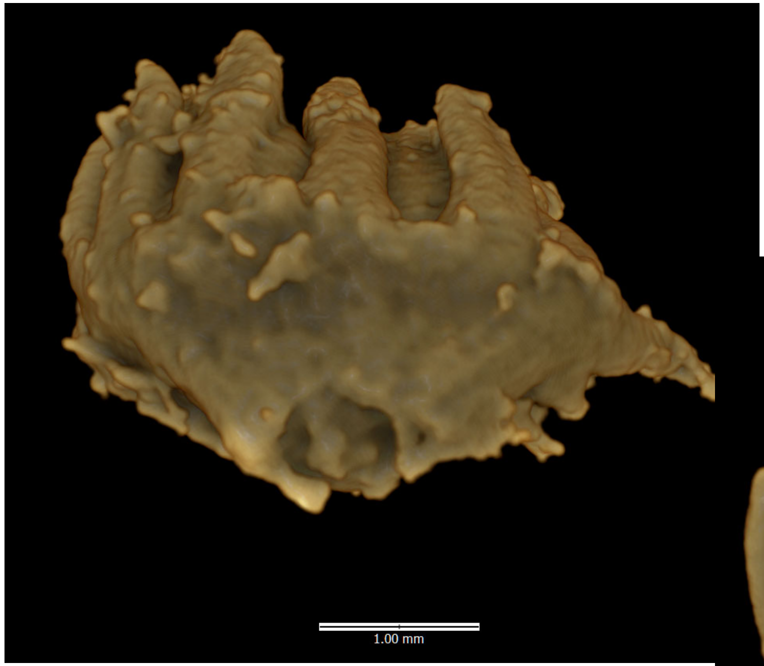

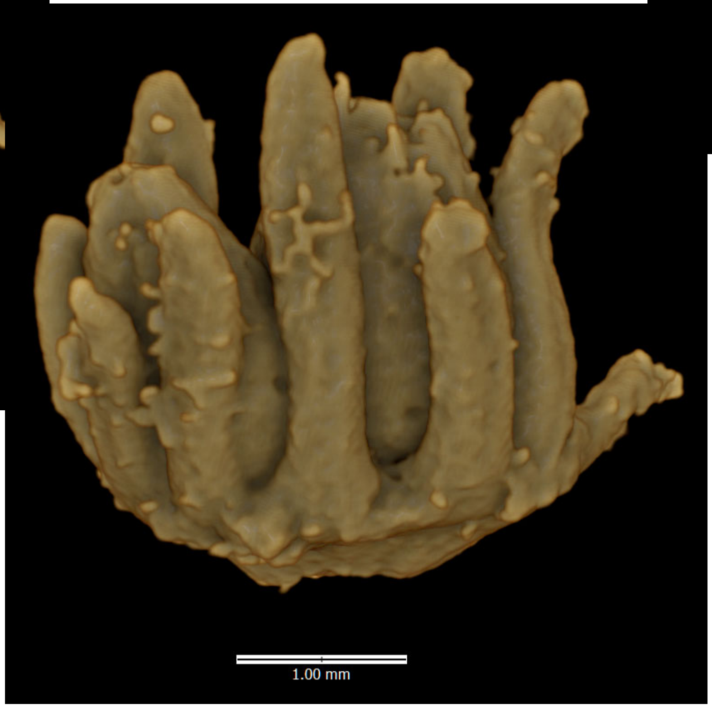

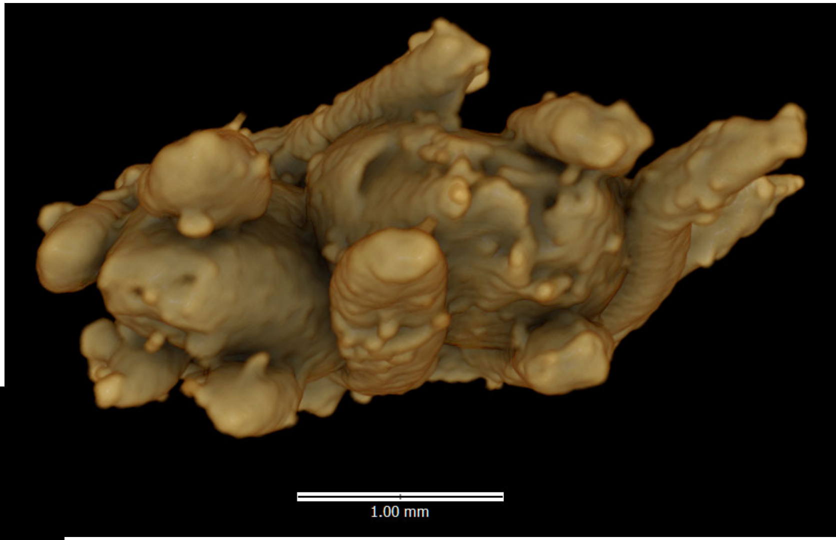

Inclusion 21. Involucre, with bristles, indeterminate attachment type and spikelet number

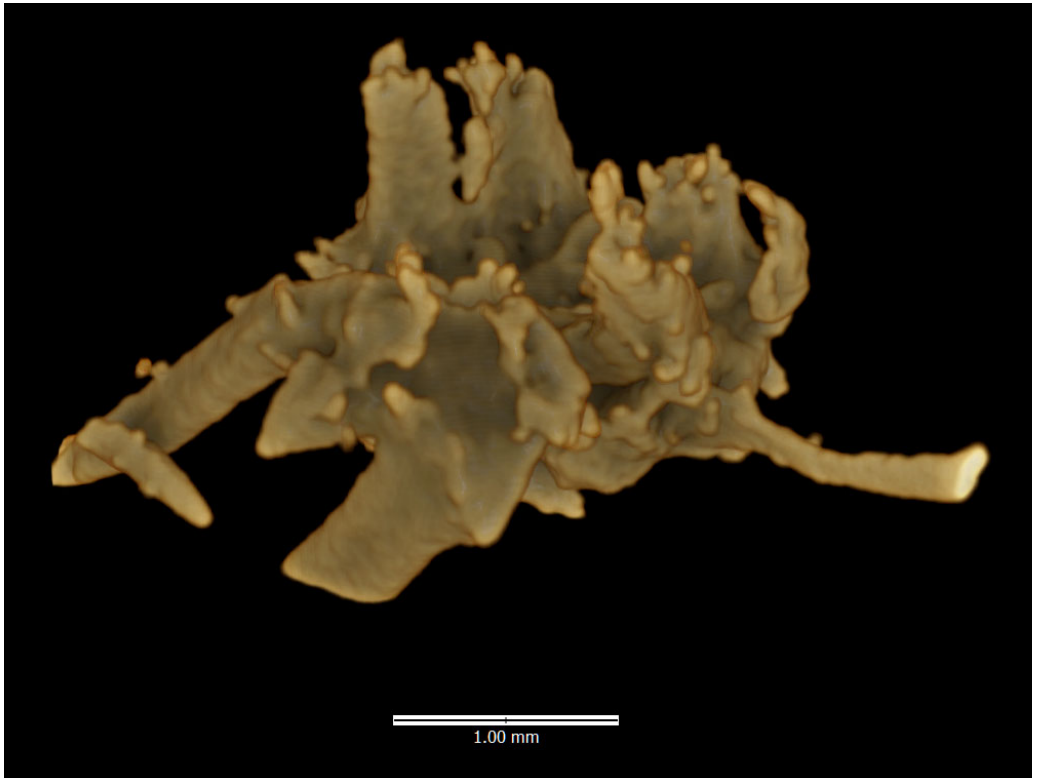

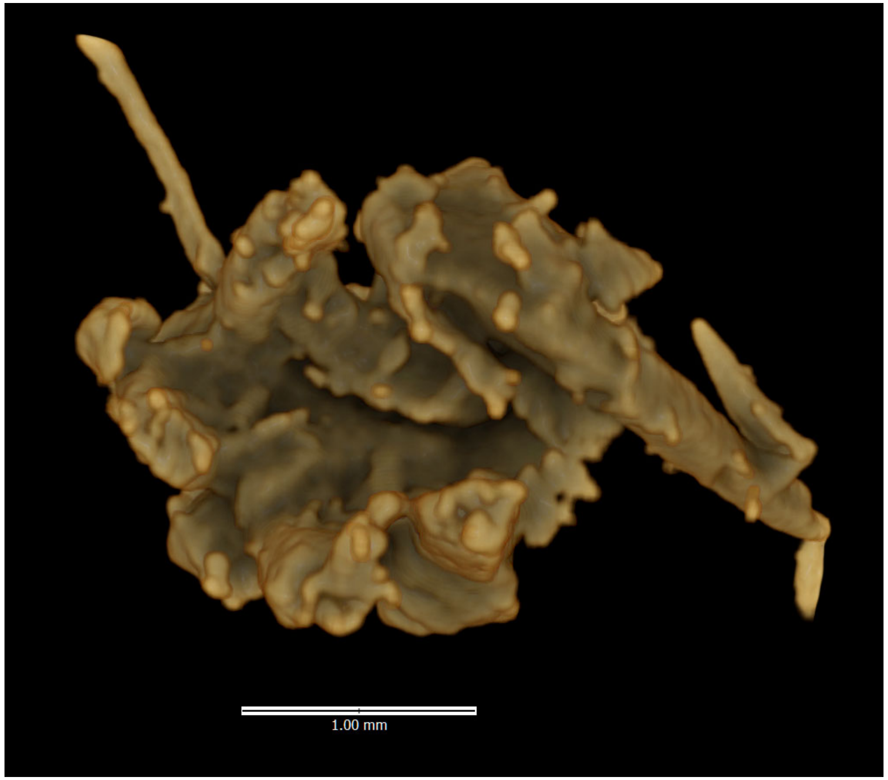

Inclusion 25. Two views: involucre with torn peduncle, indet. Spikelet number; additional bristle (at left)

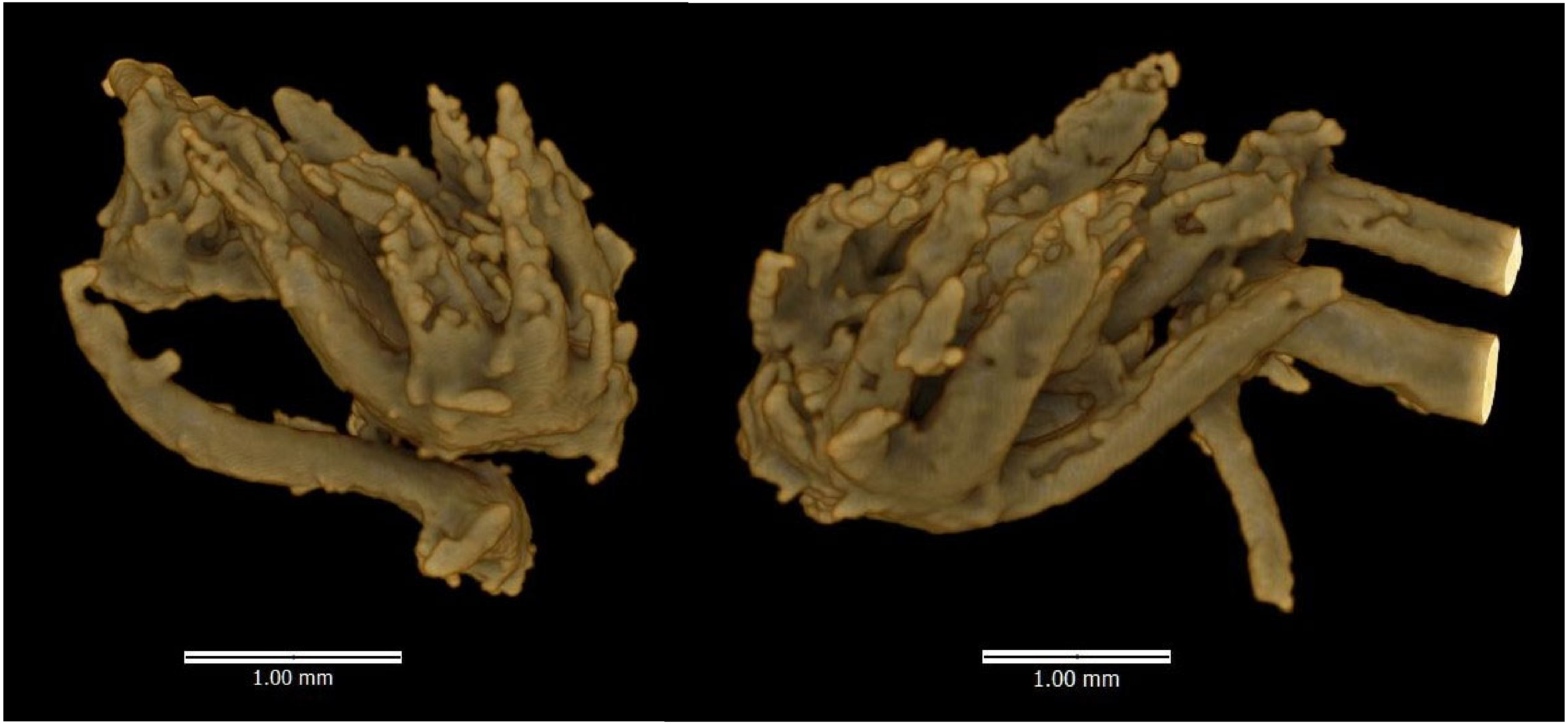

Sherd 439

Decor:
Pivoting impression
A+B Leiterband with a multiple teeth (dots and dashes) comb

Impression pivotante
A+B Leiterband avec un peigne à dents multiples mixtes.
https://lampea.cnrs.fr/cerafim/spip.php?article242

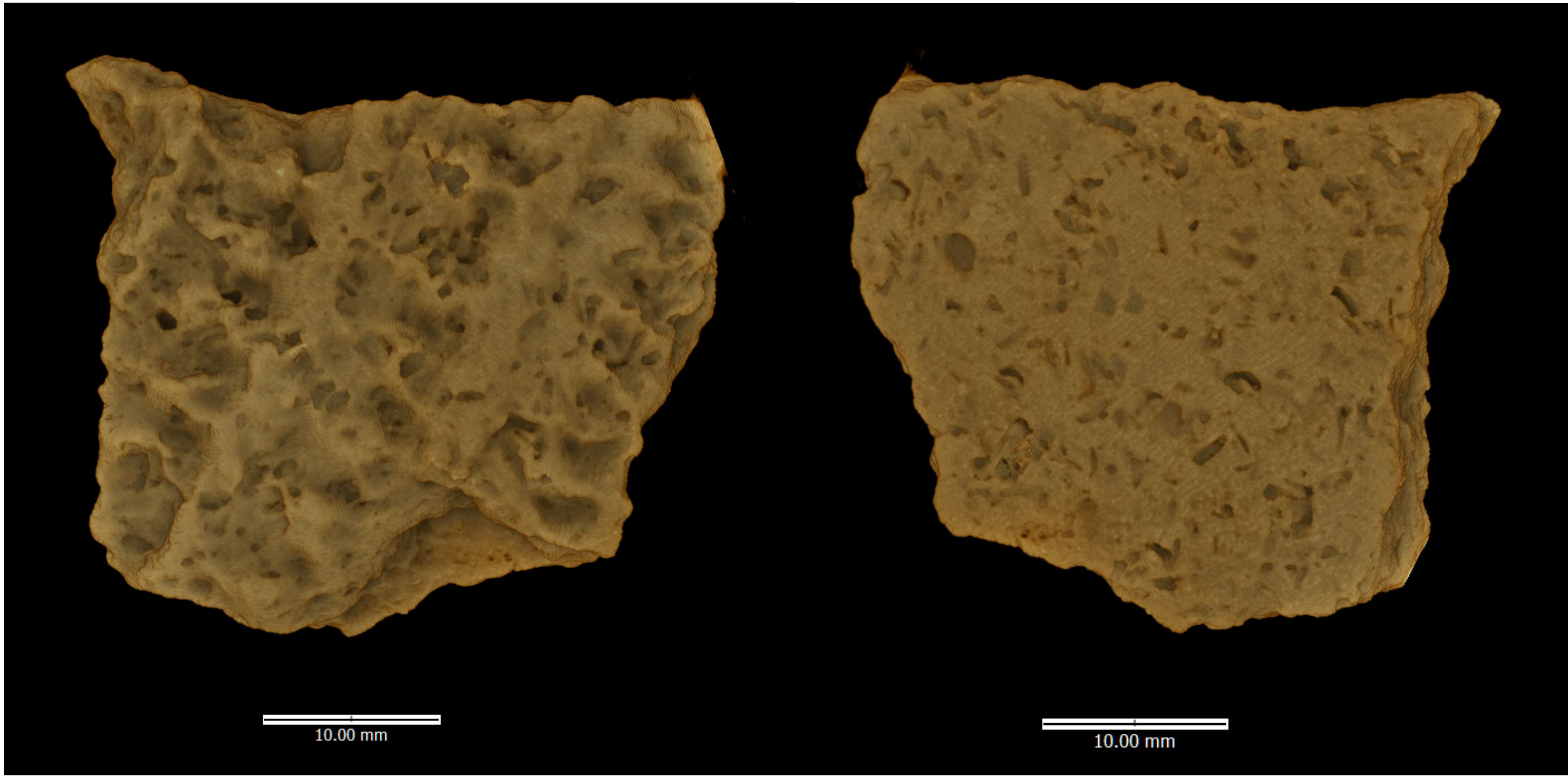

Inclusion 3.

Involucre with smooth base (sessile).

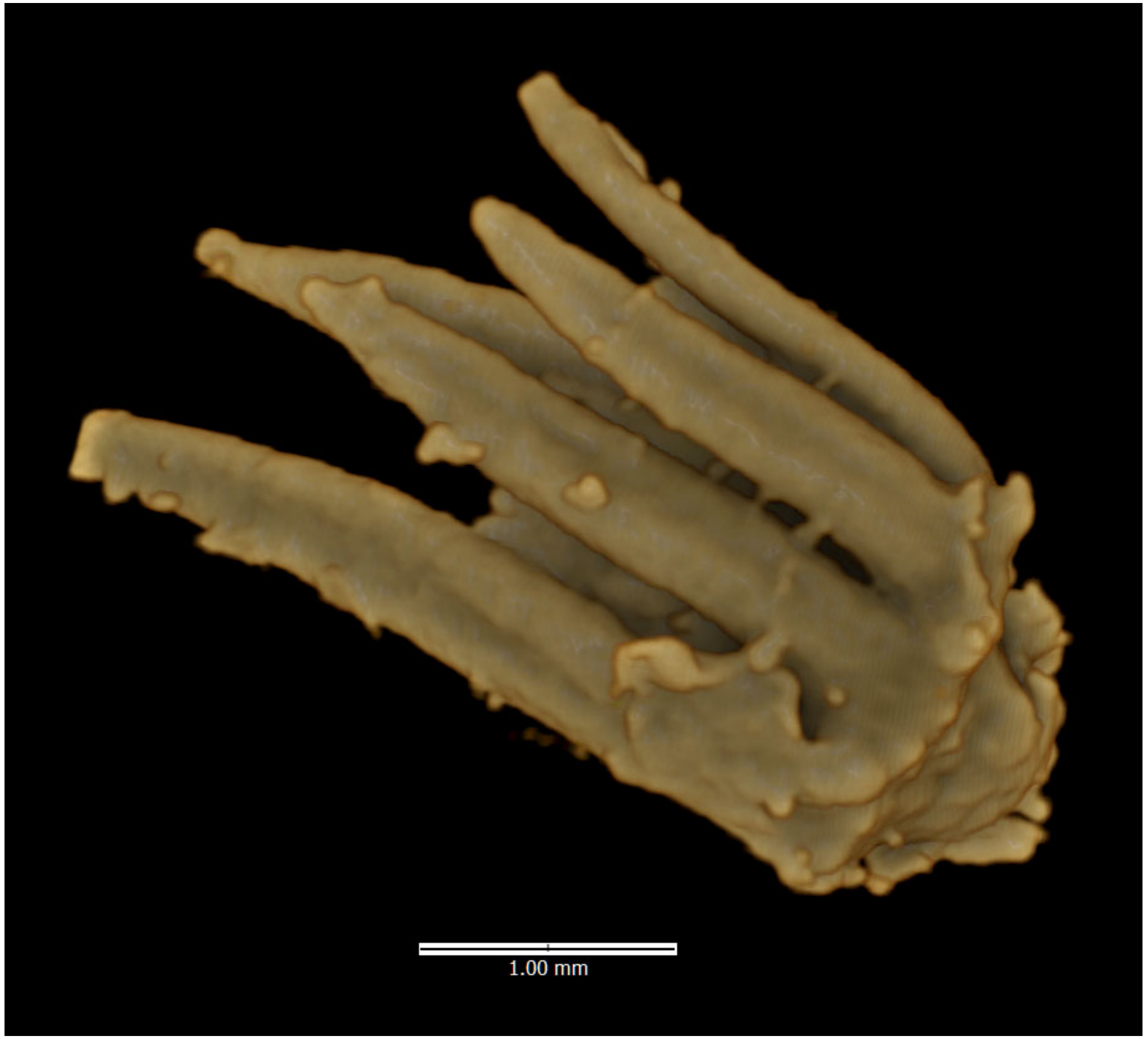

Sherd 587

No diagnostic organic inclusions

Eroded surface
Indetermined decor

Surface érodée,
Décor non identifiable

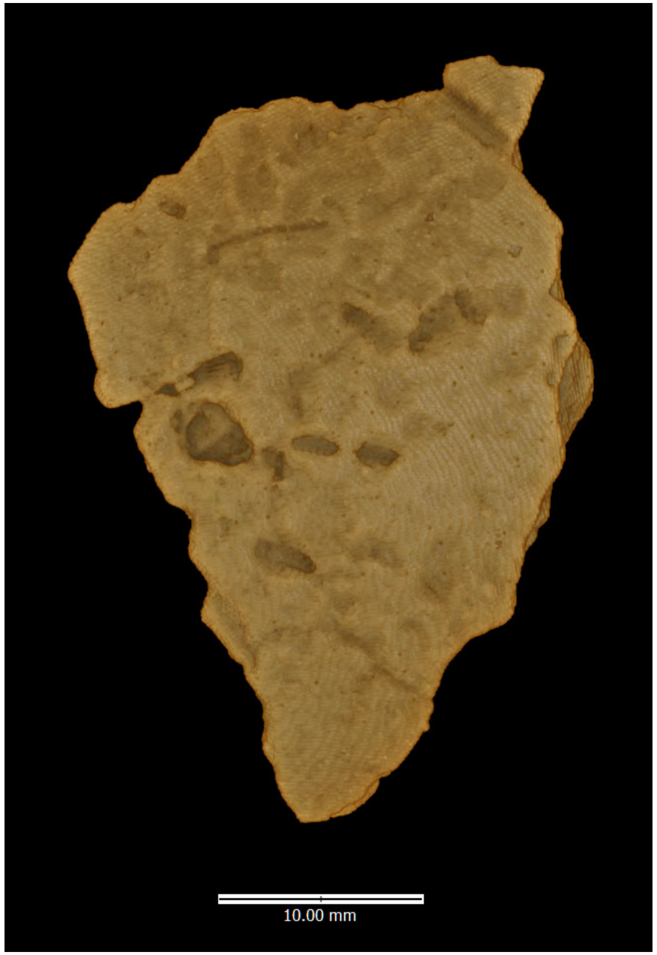

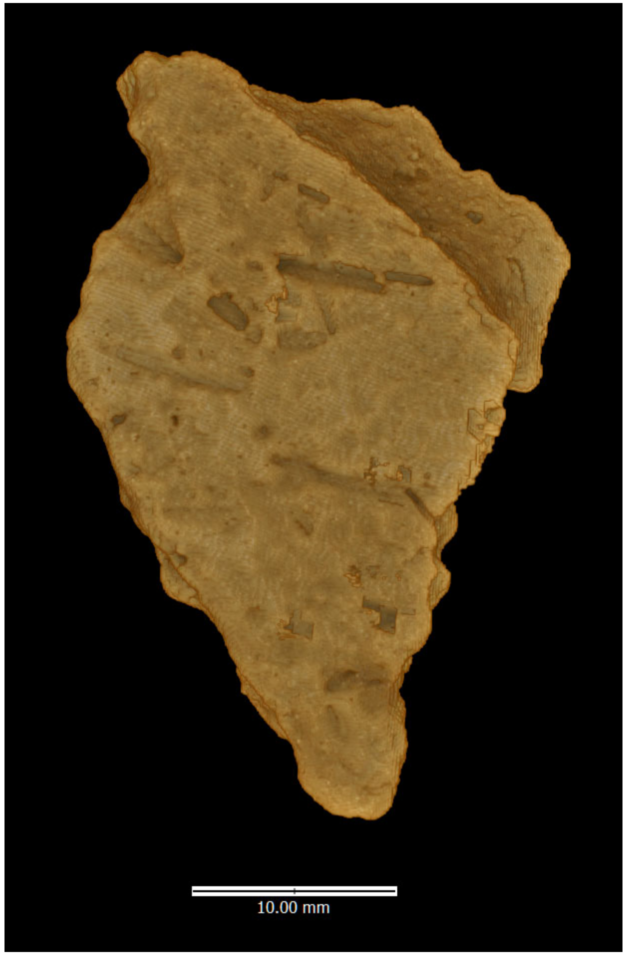

Sherd 460

No diagnostic organic inclusions

Eroded surface
Indetermined decor

Surface érodée,
Décor non identifiable

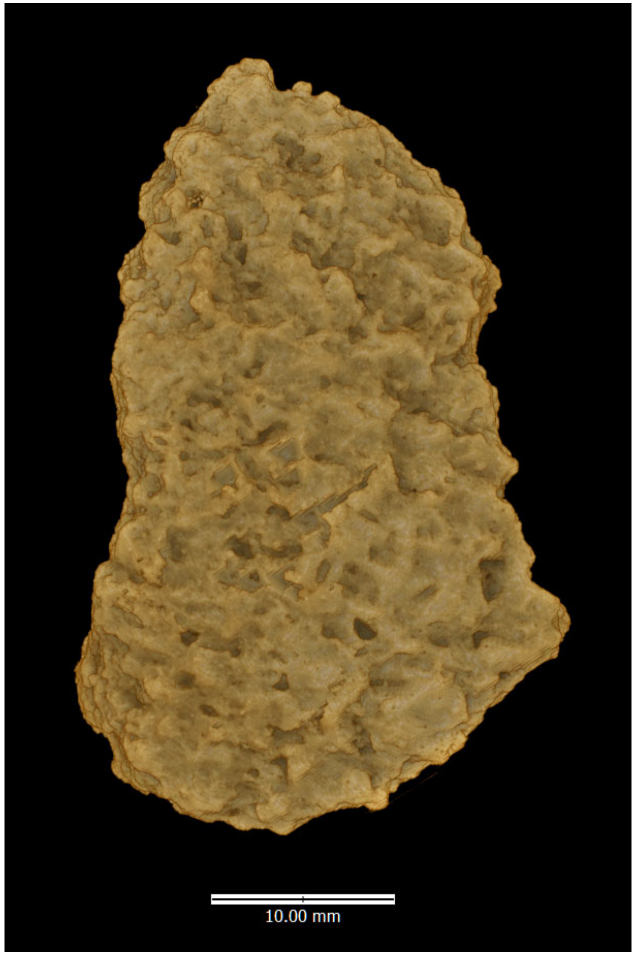

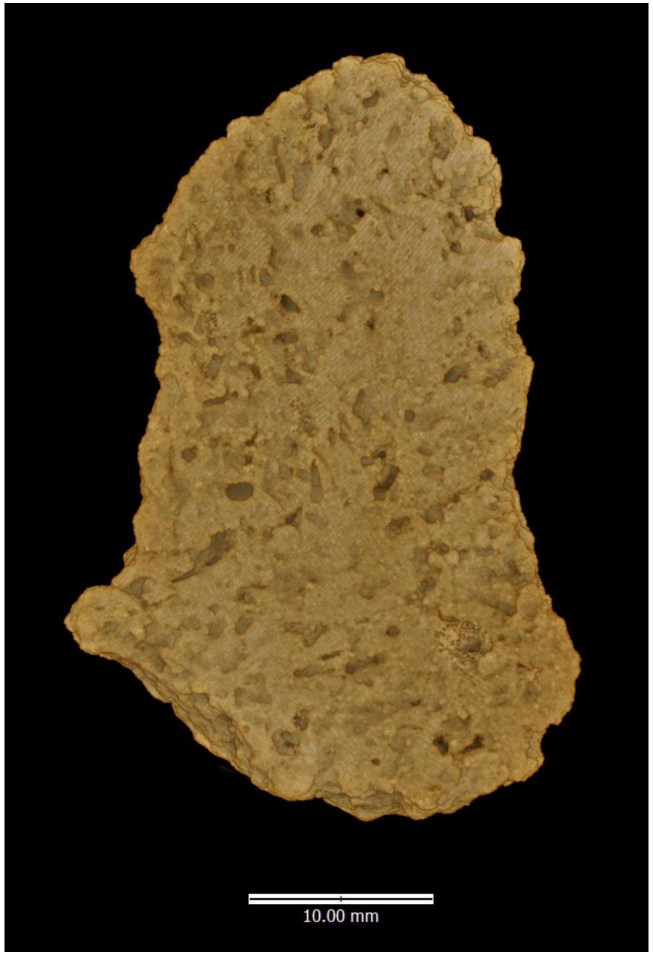

Sherd 1324

No diagnostic organic inclusions

Décor:
Simple or pivoting impression A+B with a rectilinear edge spatula

Impression simple ou pivotante avec spatule à front droit

https://lampea.cnrs.fr/cerafim/spip.php?article238

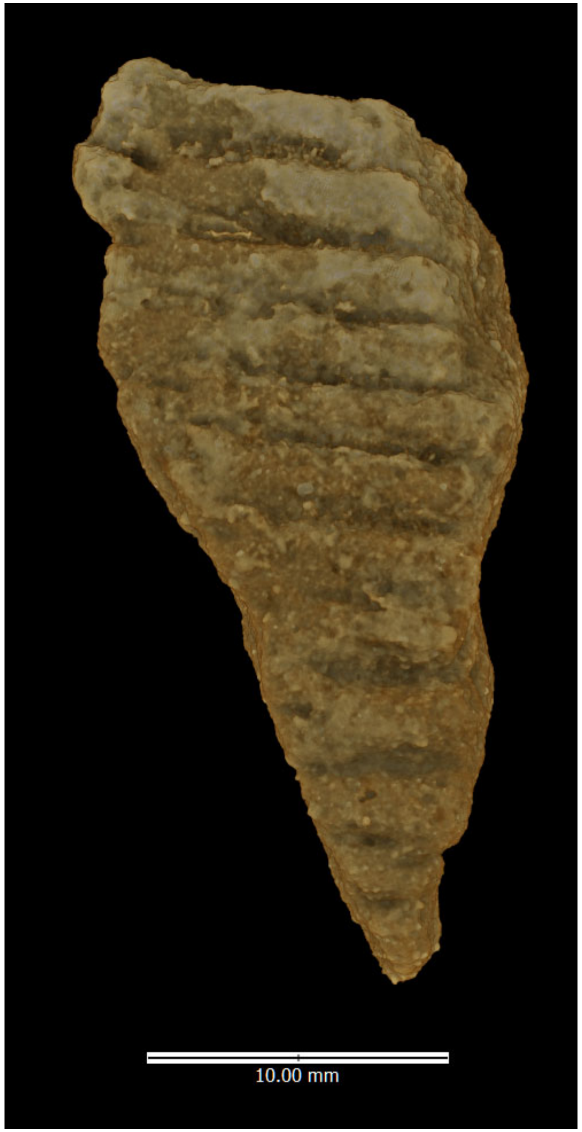

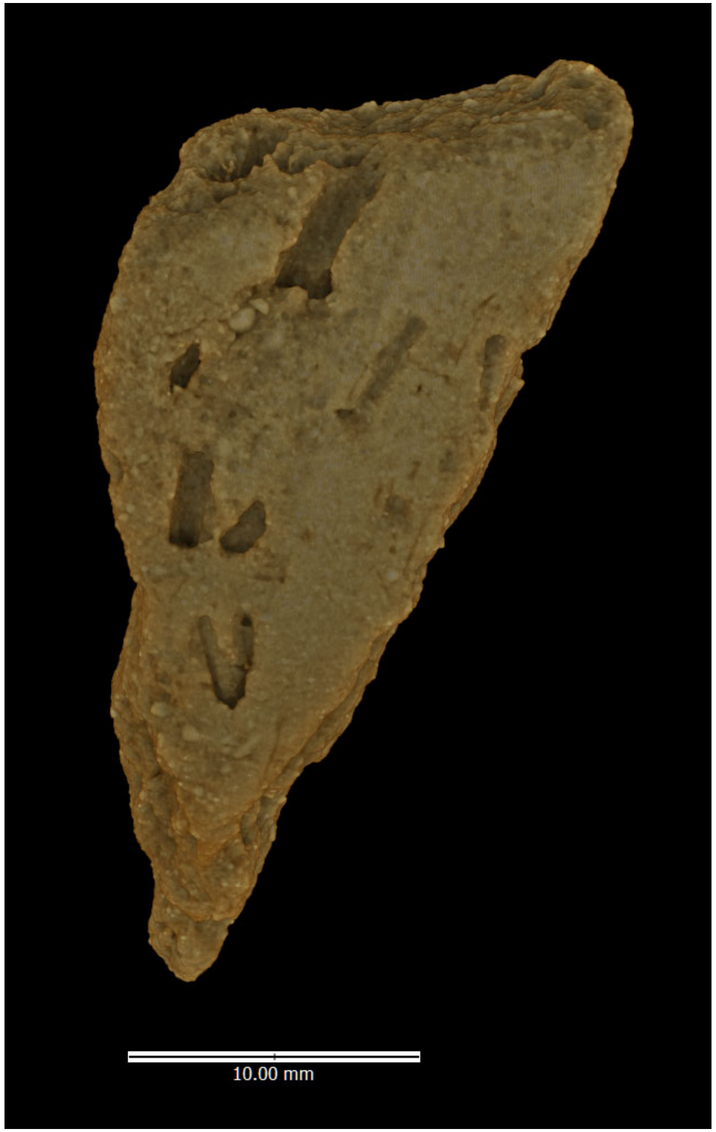

AZ22

Sherd 27

Décor:
Pivoting impression A+B with a multiple teeth (dashes) comb

Impression pivotante A+B avec peigne à dents multiples plates

https://lampea.cnrs.fr/cerafim/spip.php?article240

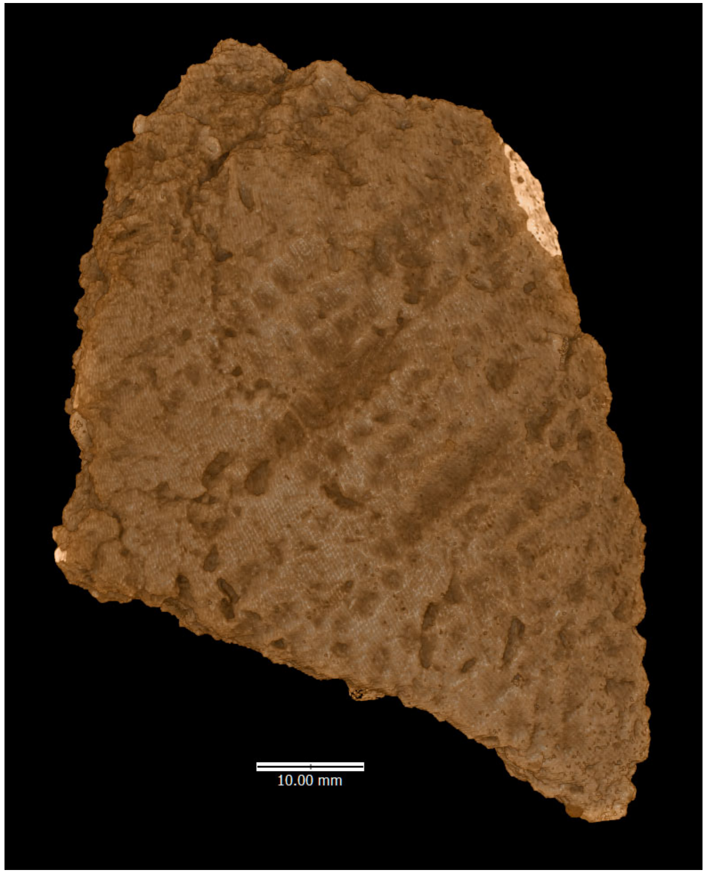

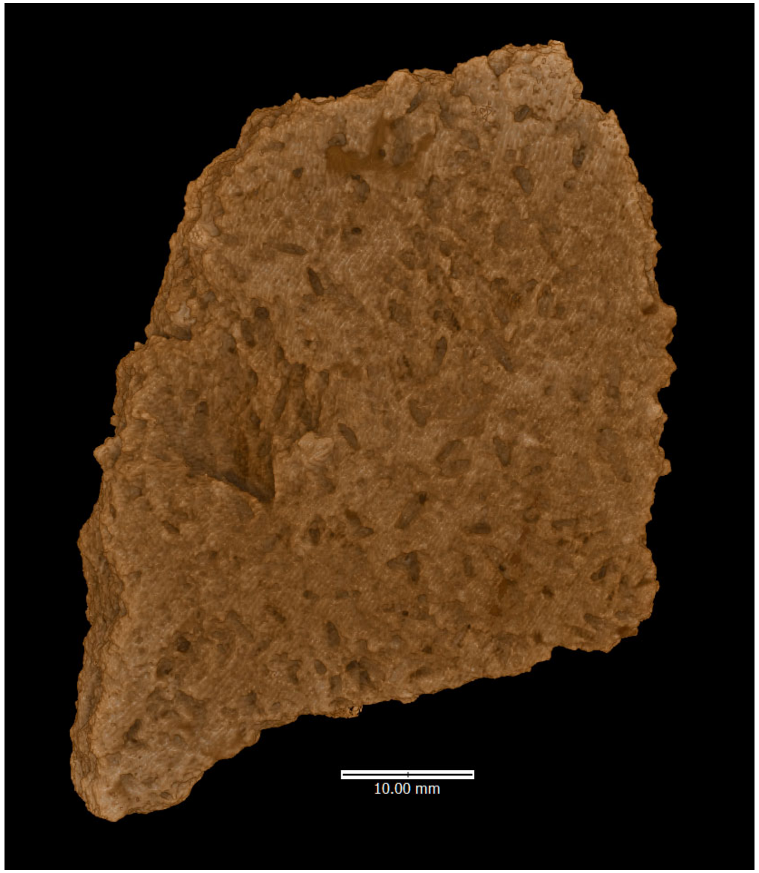

Inclusion 1. Two views: Sessile involucre, solitary spikelet

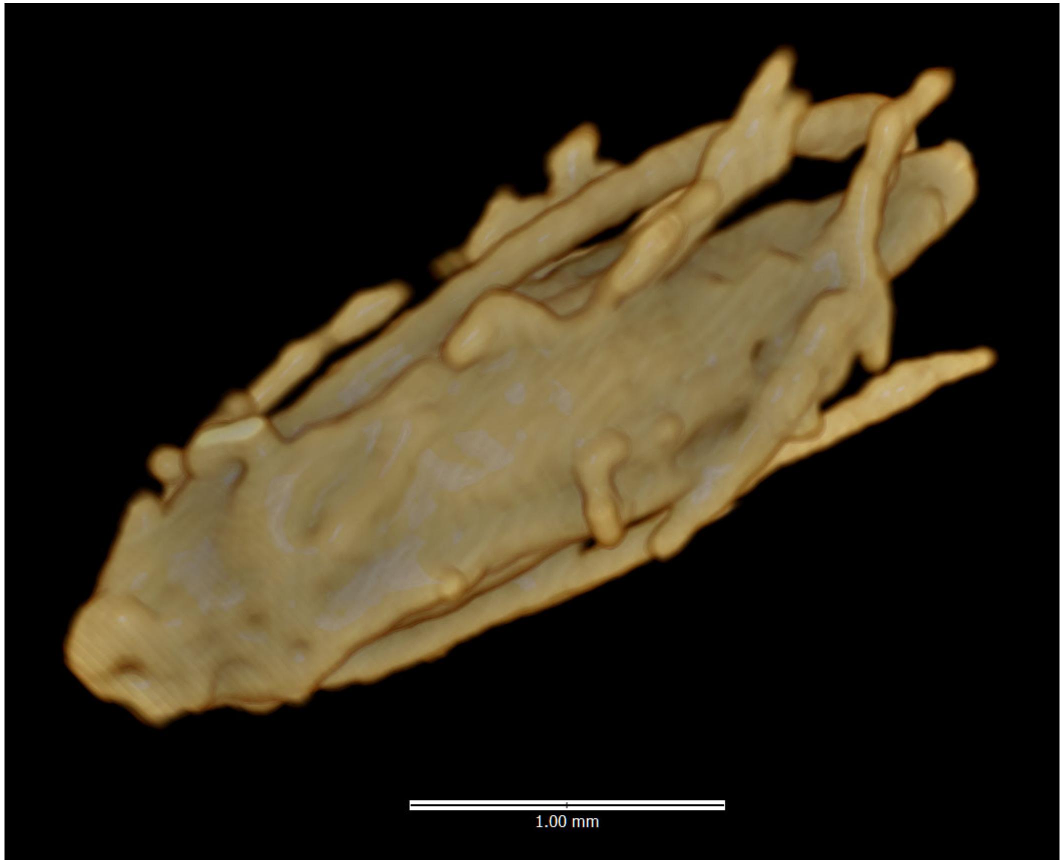

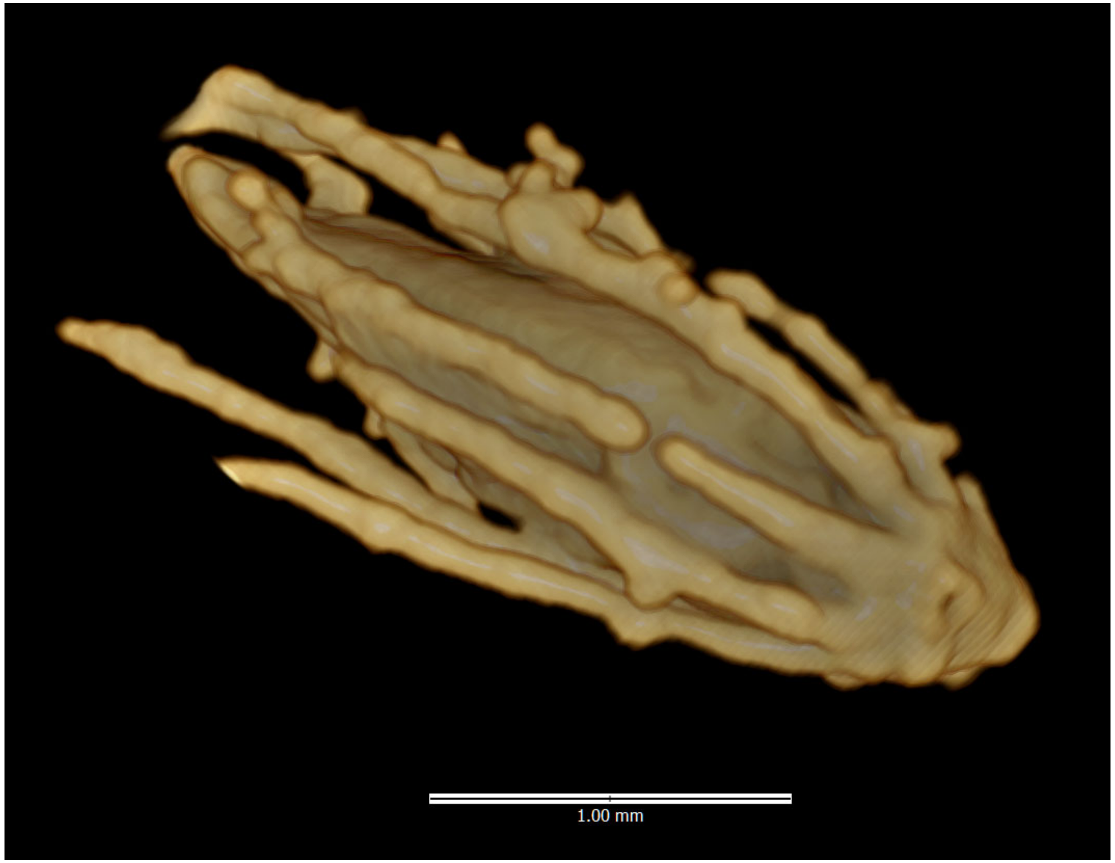

Inclusion 4a. Two views: Sessile involucre, solitary spikelet

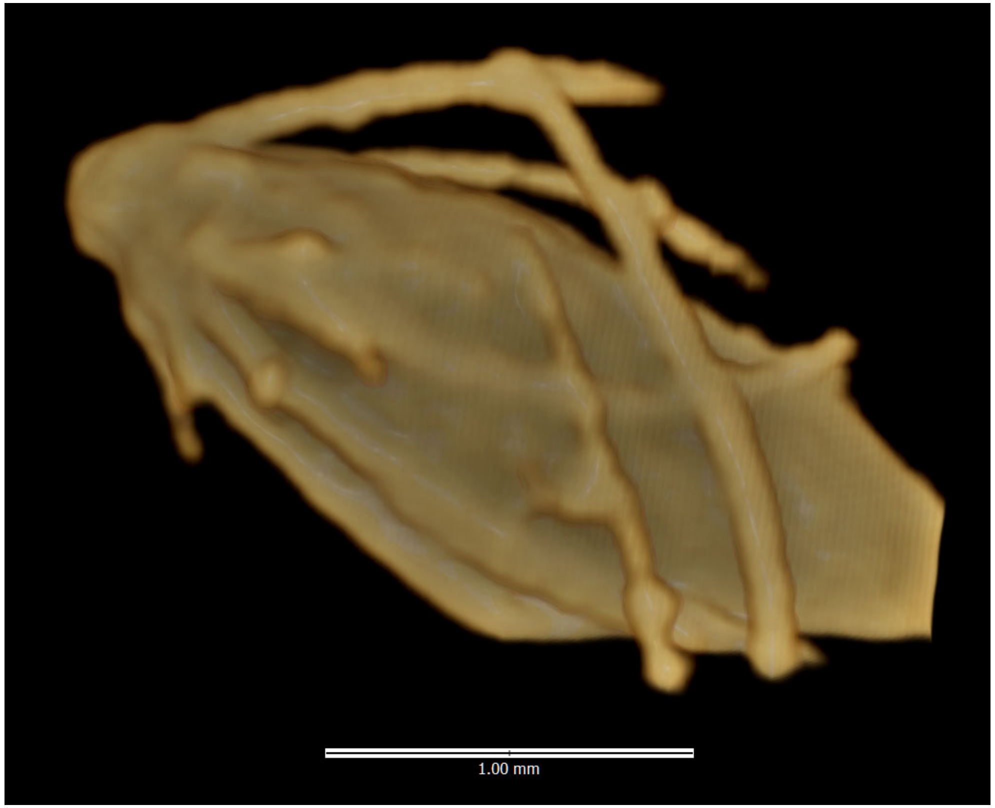

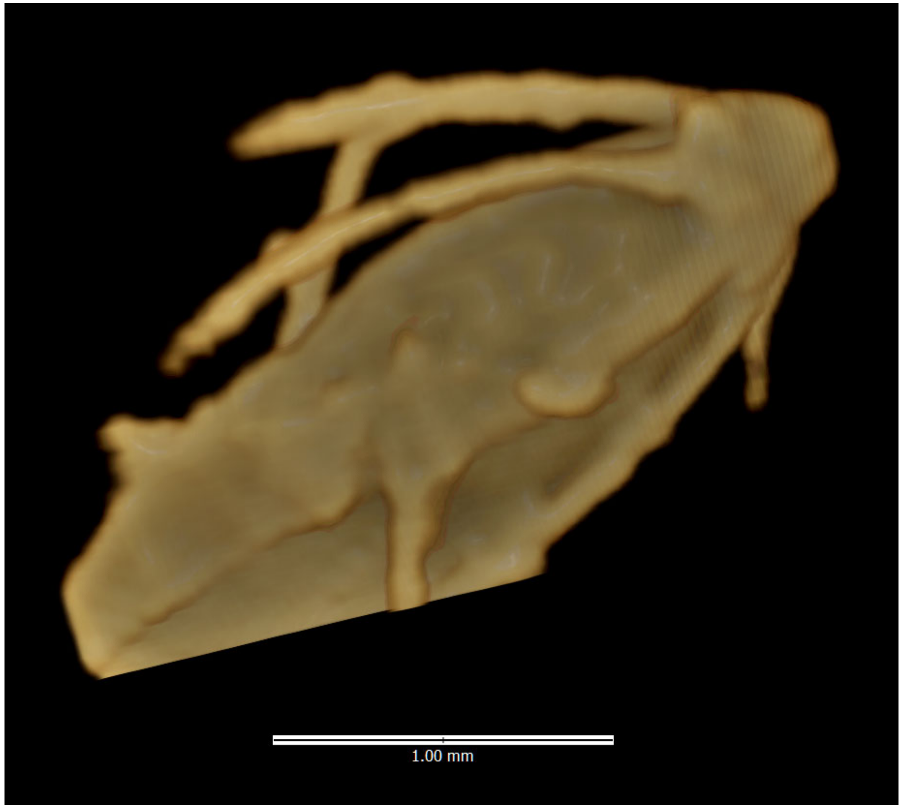

Inclusion 5. Two views: Sessile involucre, solitary spikelet

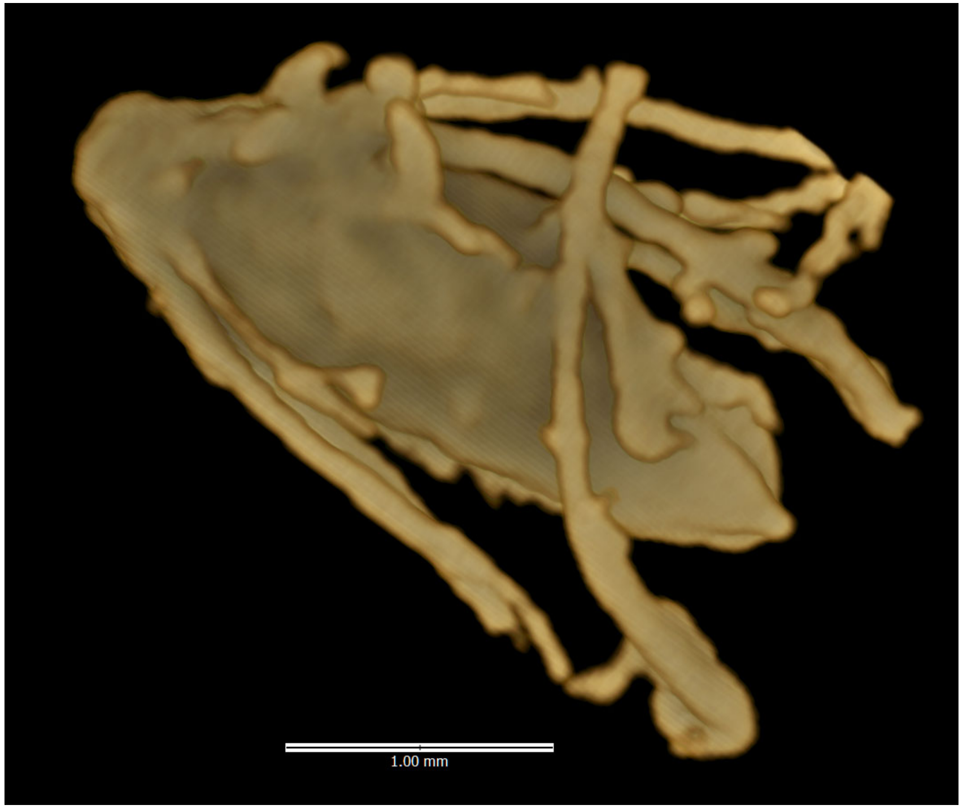

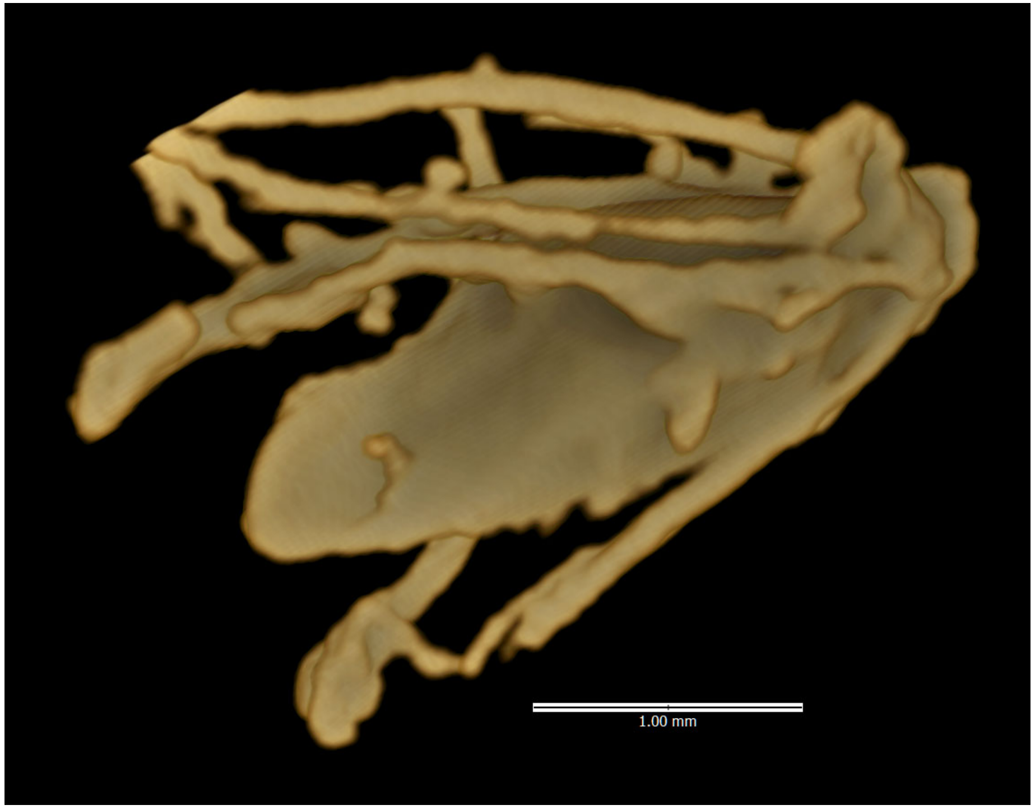

Inclusion 7. Two views: Sessile involucre, solitary spikelet

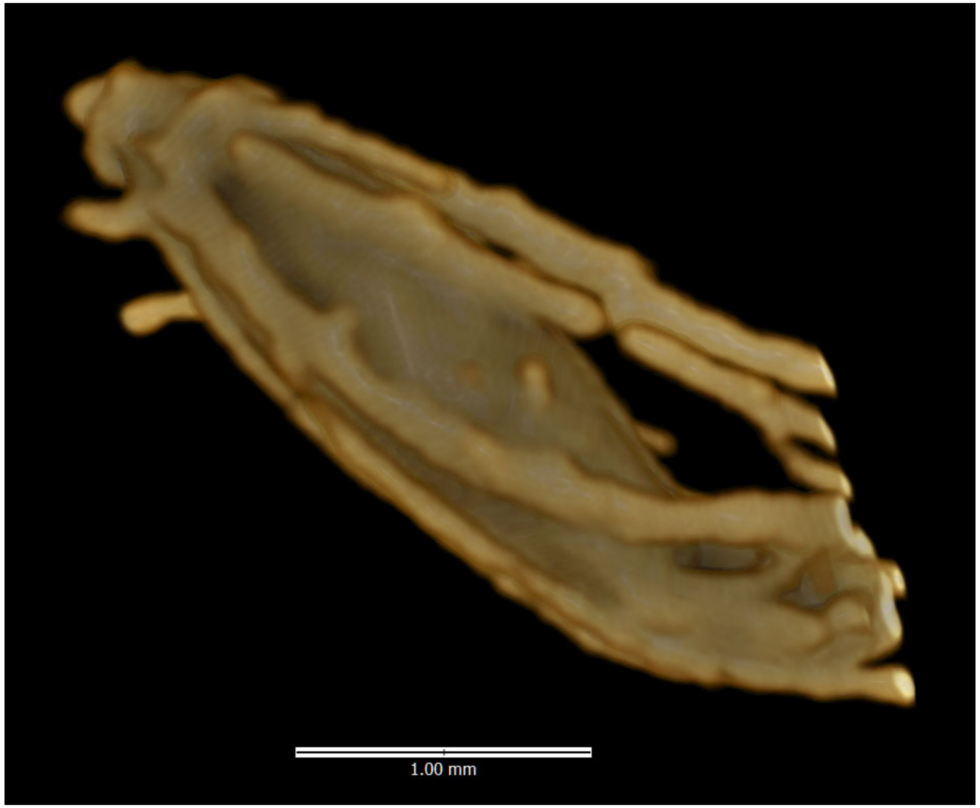

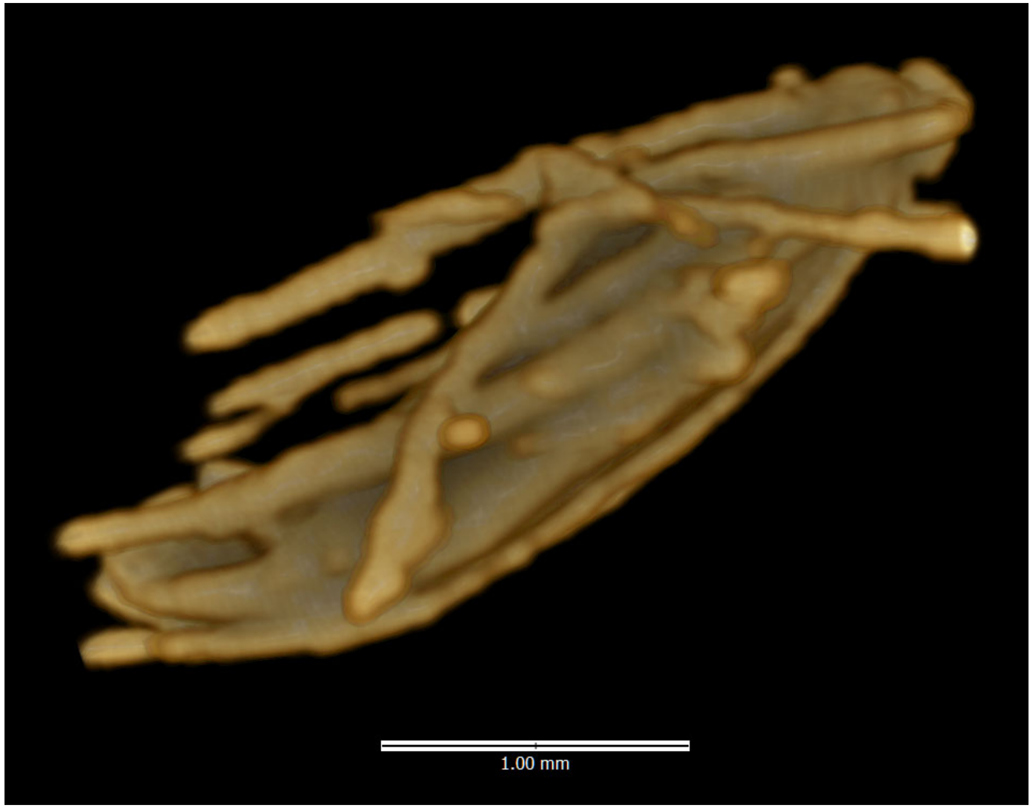

Inclusion 8. Two views: Sessile involucre, solitary spikelet

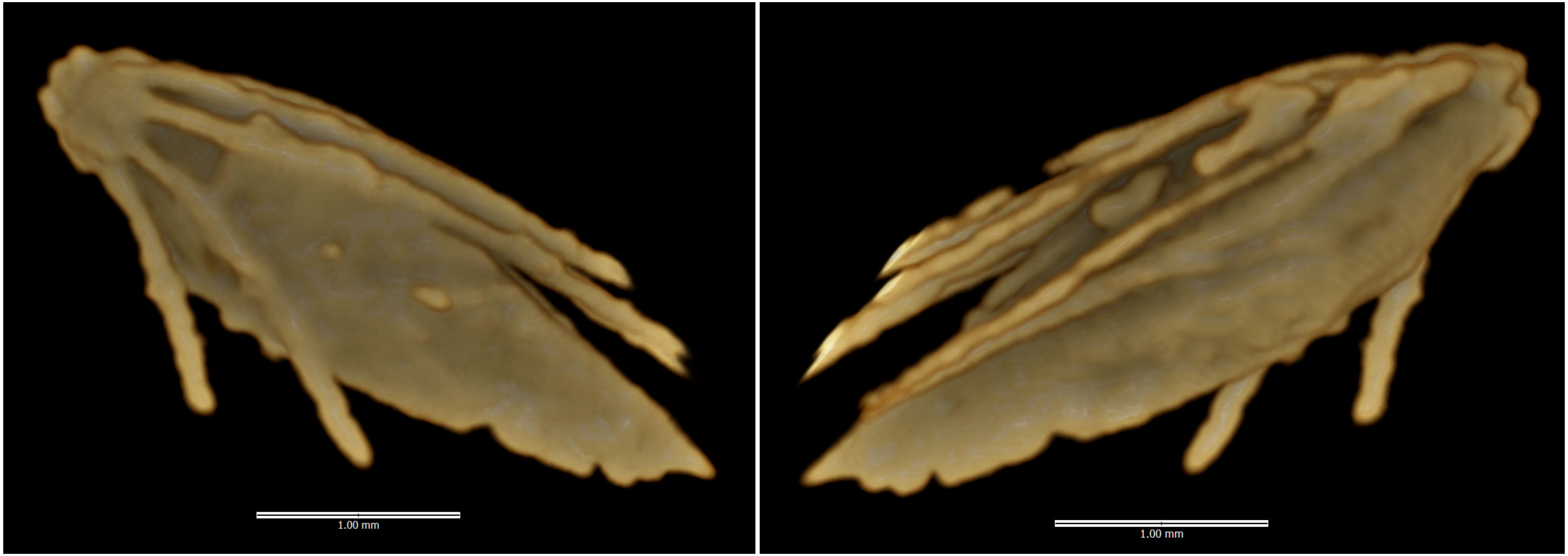

Inclusion 9. Two views: Sessile involucre, solitary spikelet

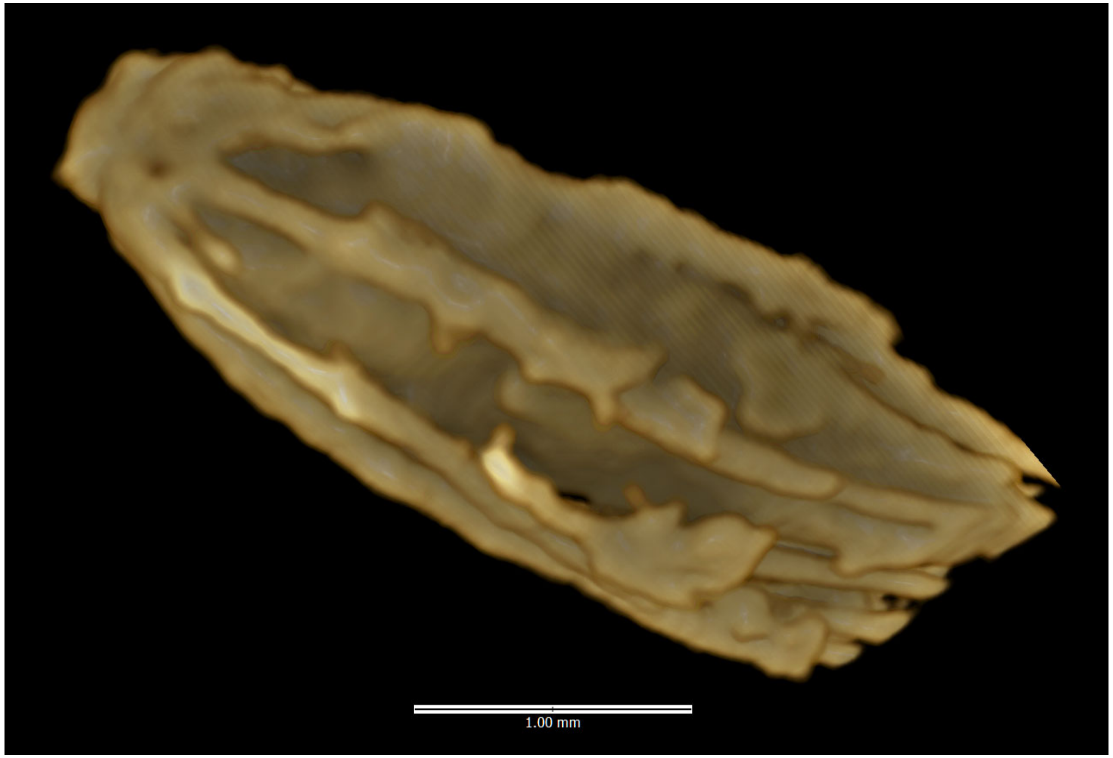

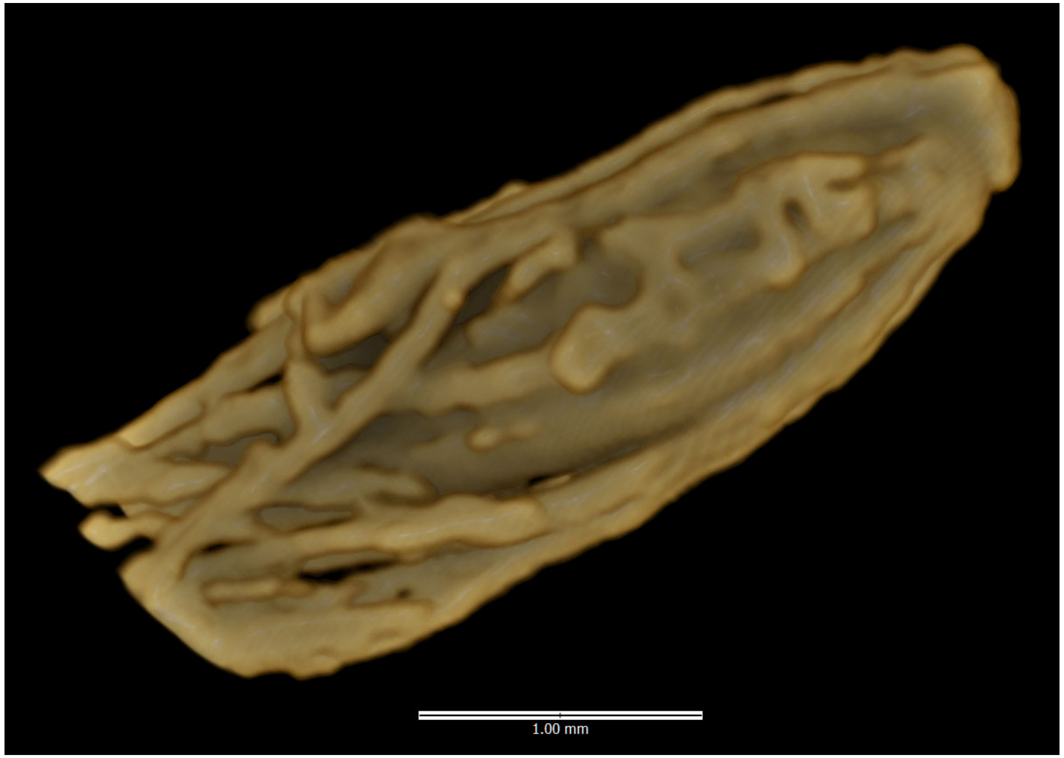

Inclusion 11. Two views: Sessile involucre, solitary spikelet

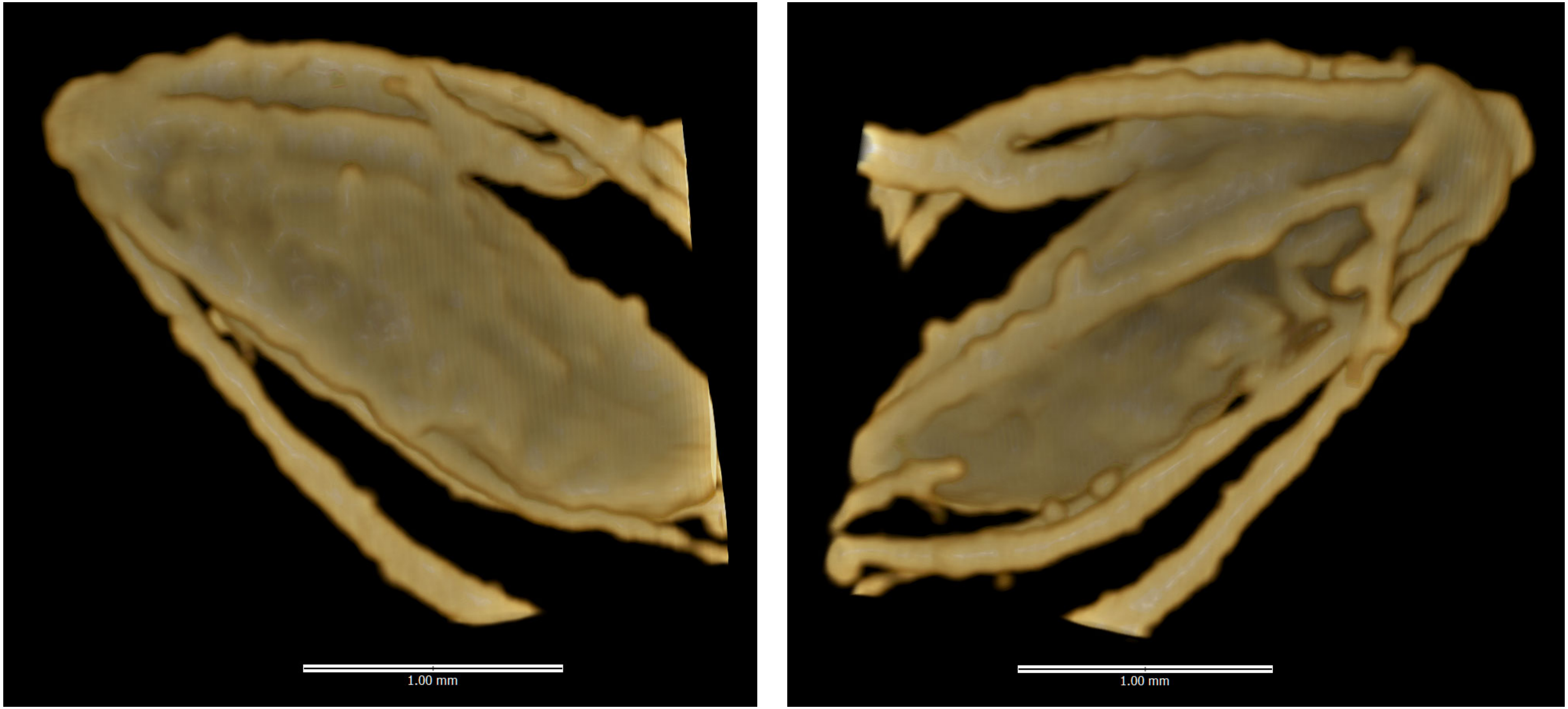

Inclusion 12. Two views: Sessile involucre, solitary spikelet

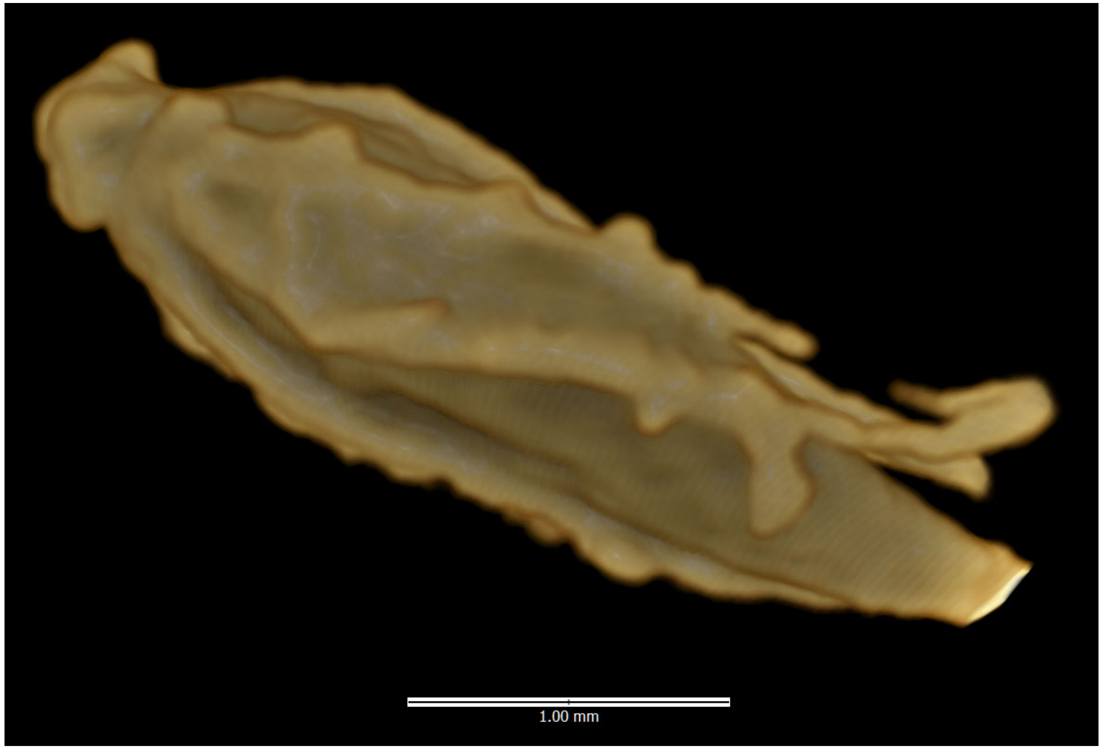

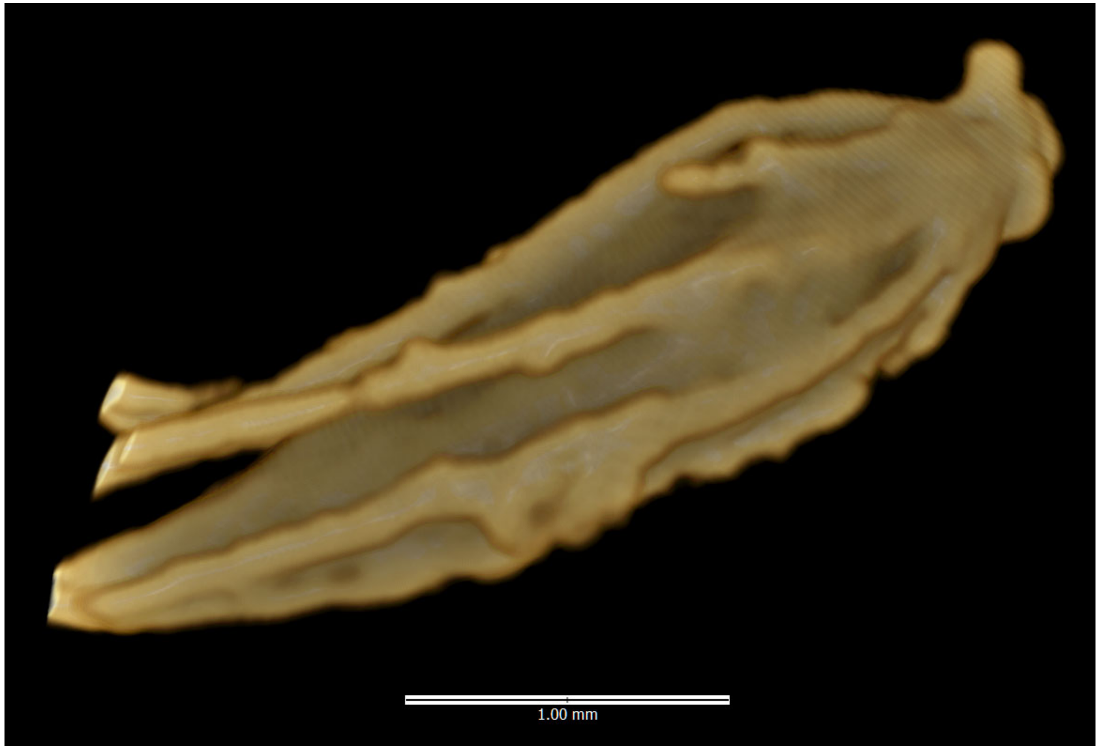

Inclusion 13. Two views: Sessile involucre, solitary spikelet

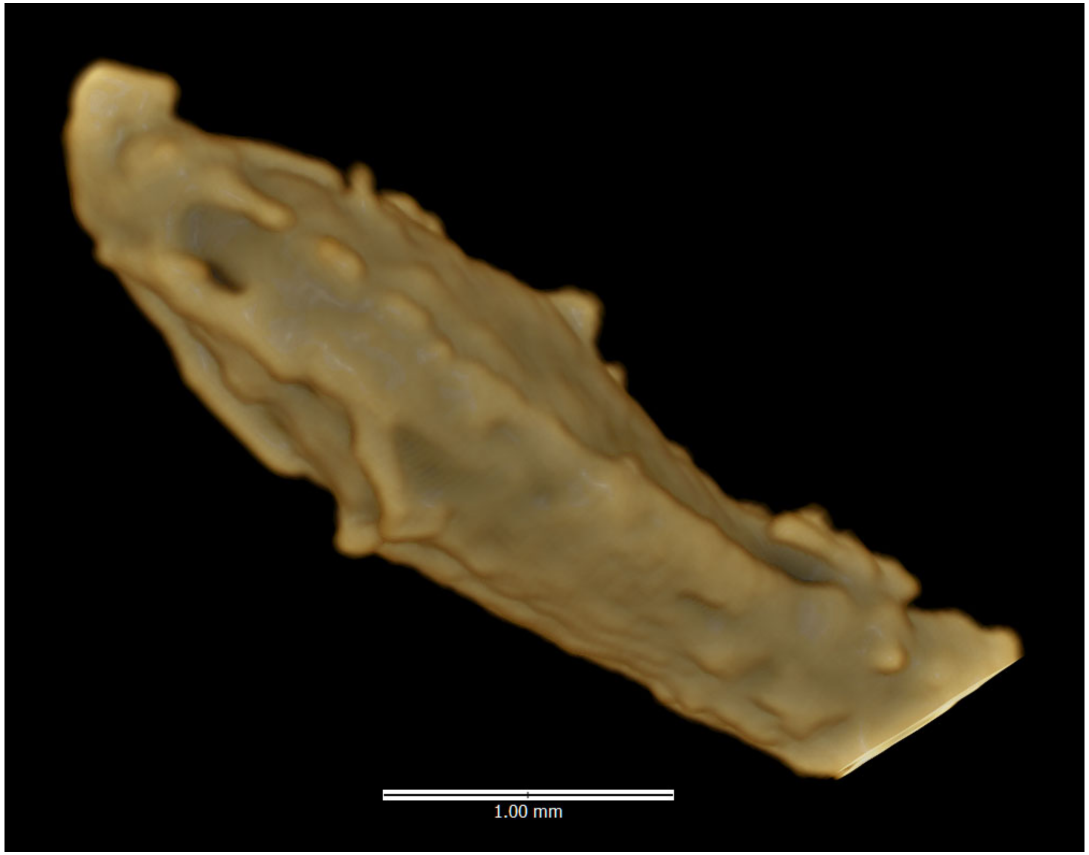

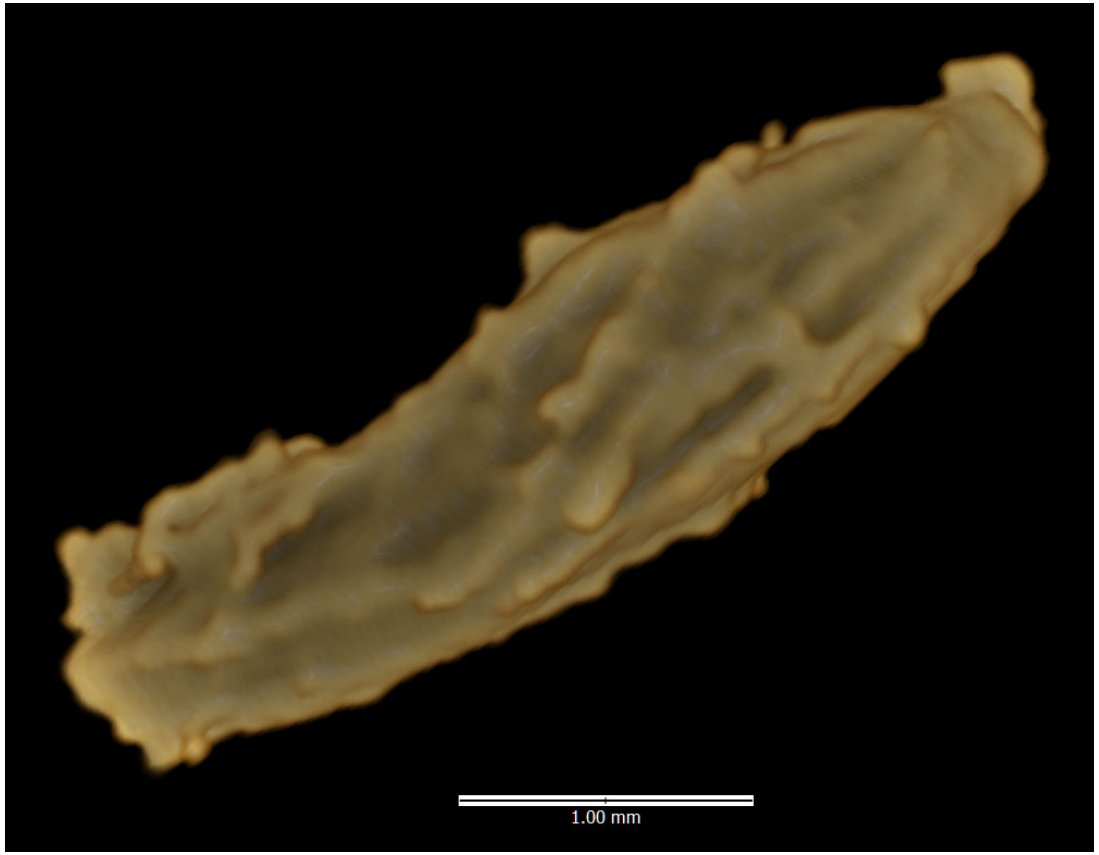

Inclusion 14. Two views: Sessile involucre, solitary spikelet

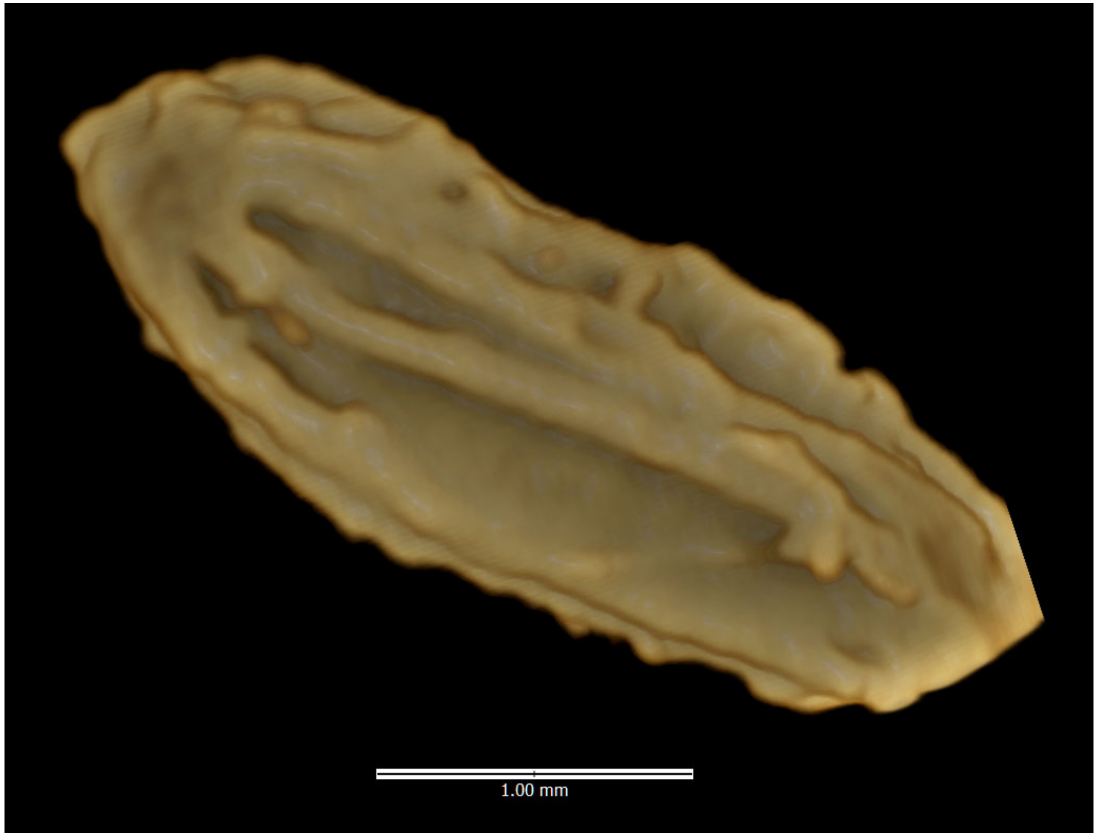

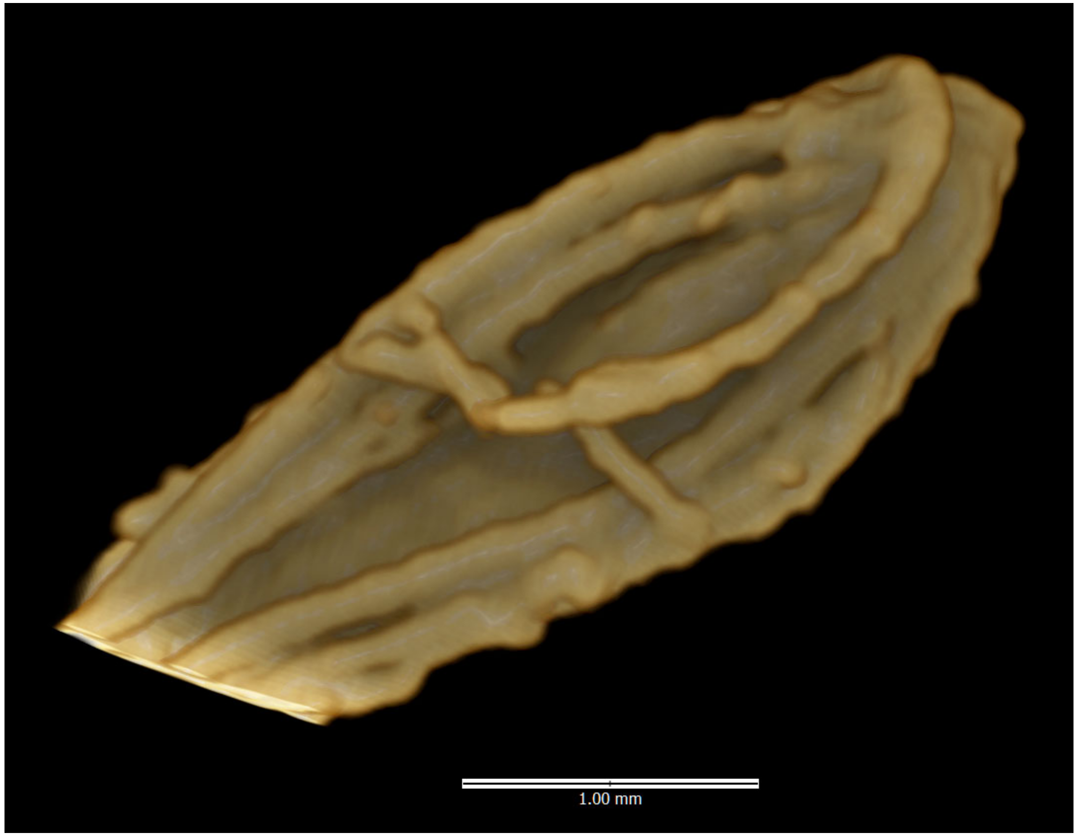

Inclusion 15. Two views: Sessile involucre, solitary spikelet

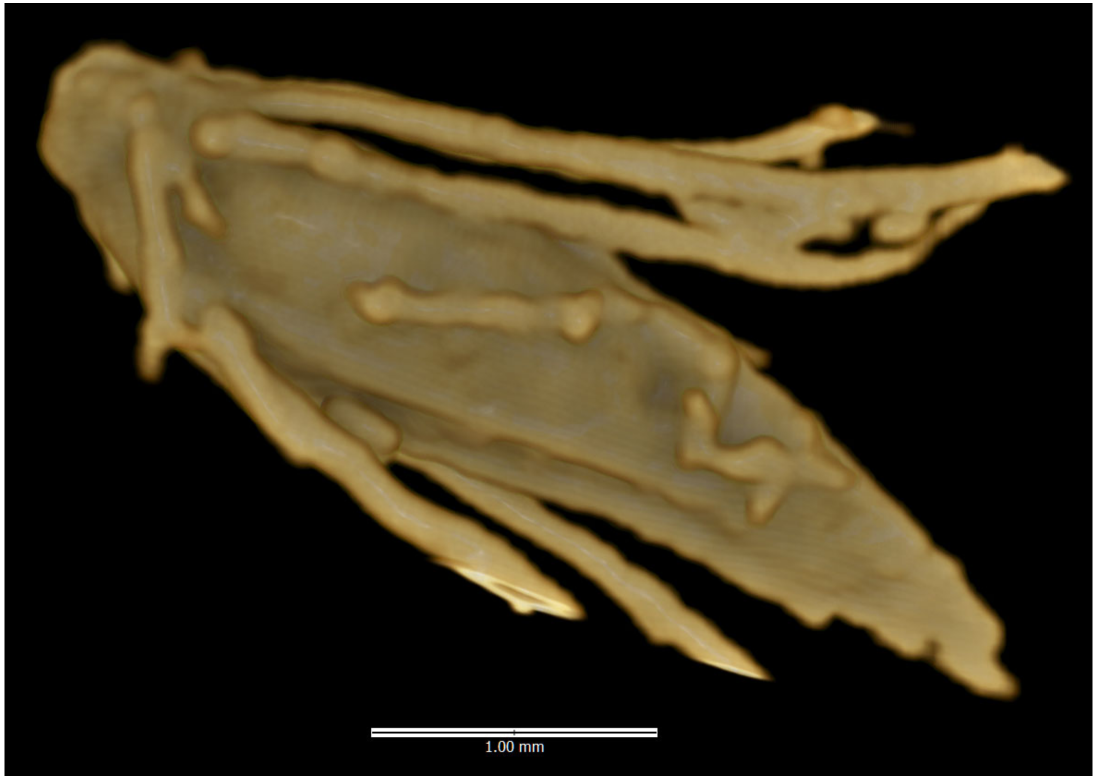

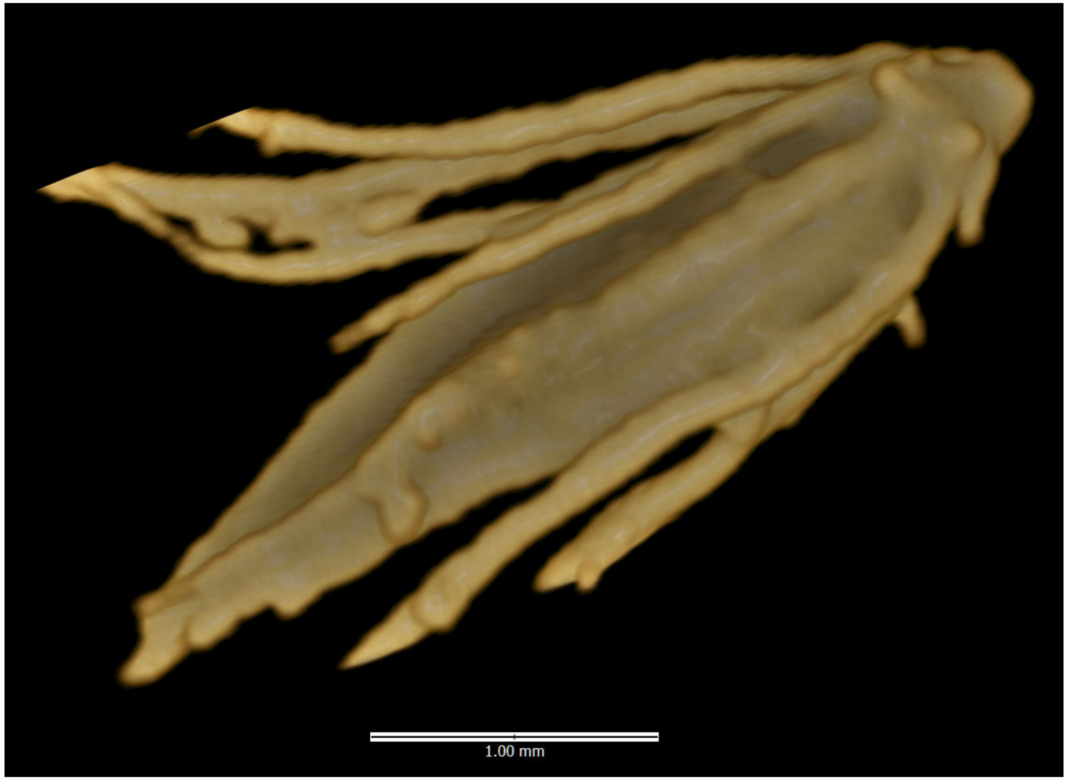

Inclusion 16. Two views: Sessile involucre, solitary spikelet

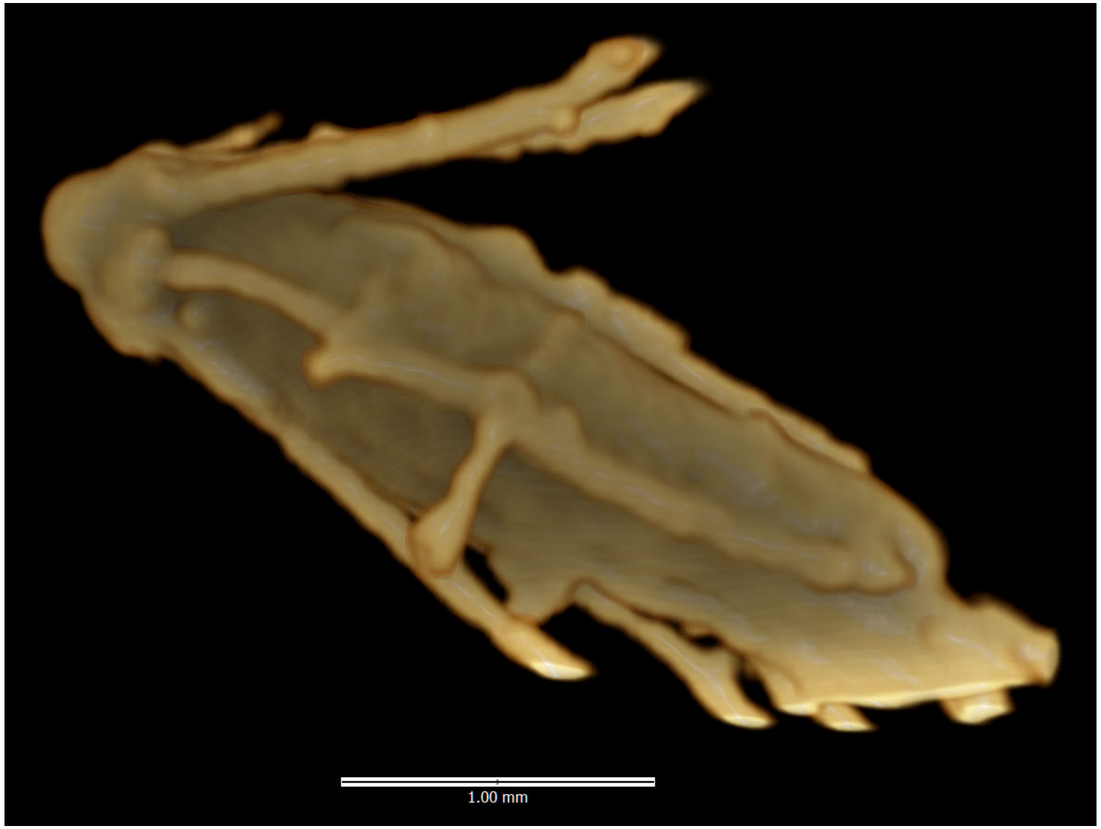

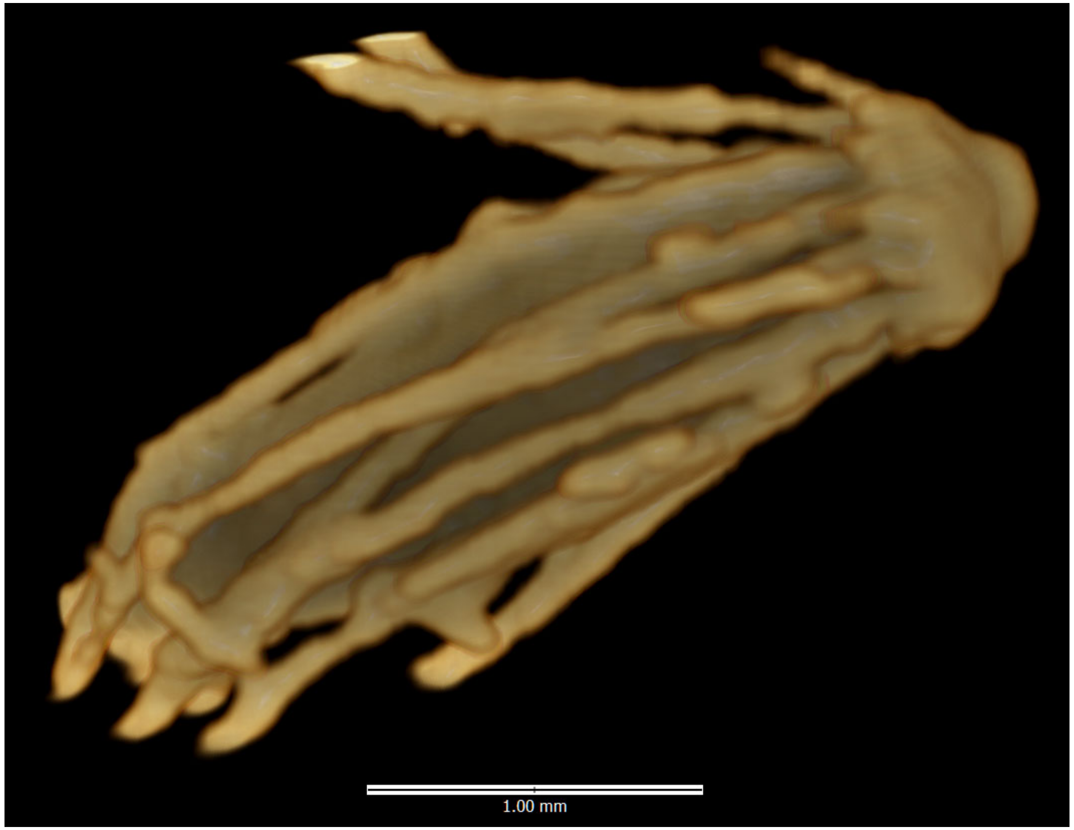

Inclusion 17. Two views: Sessile involucre, solitary spikelet

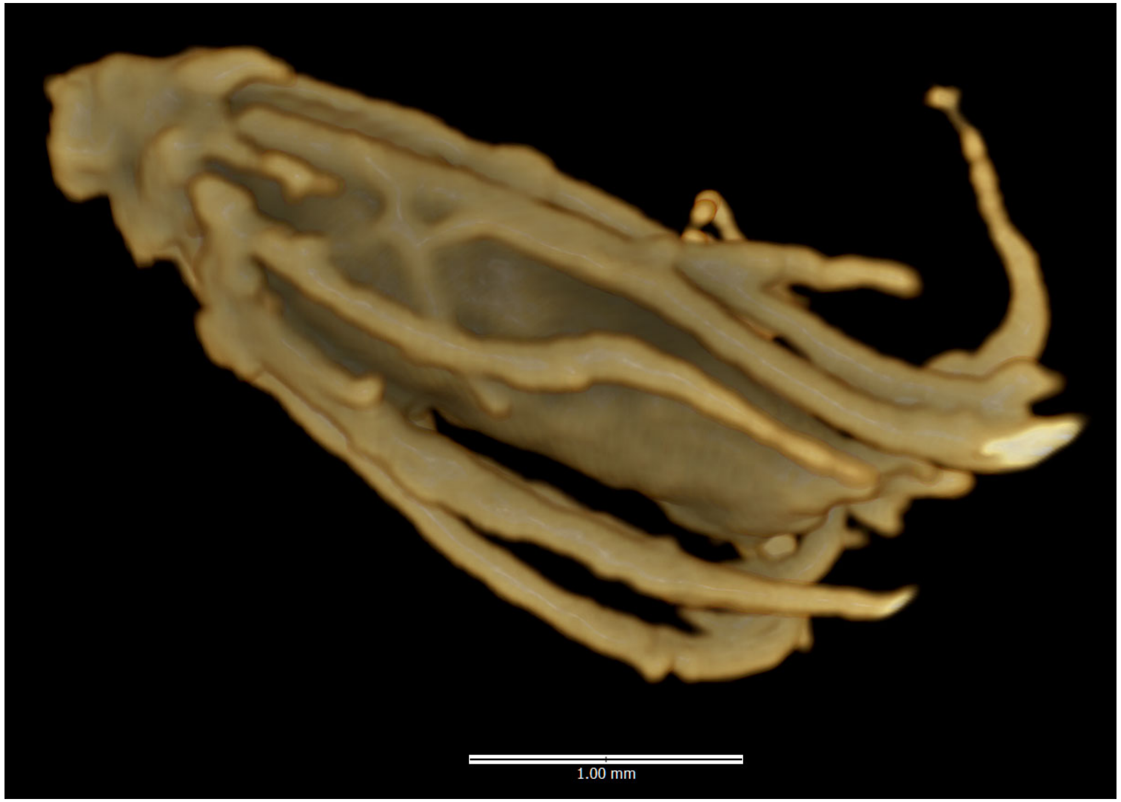

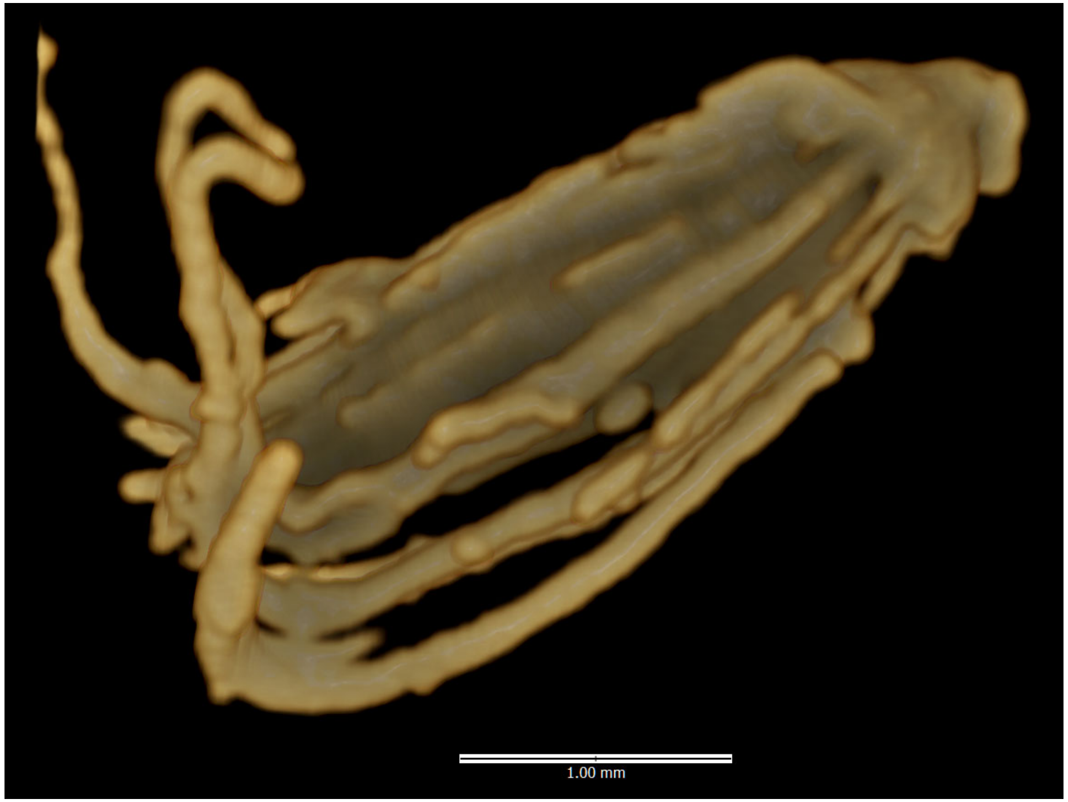

Inclusion 19. involucre, indeterminate attachment, solitary spikelet

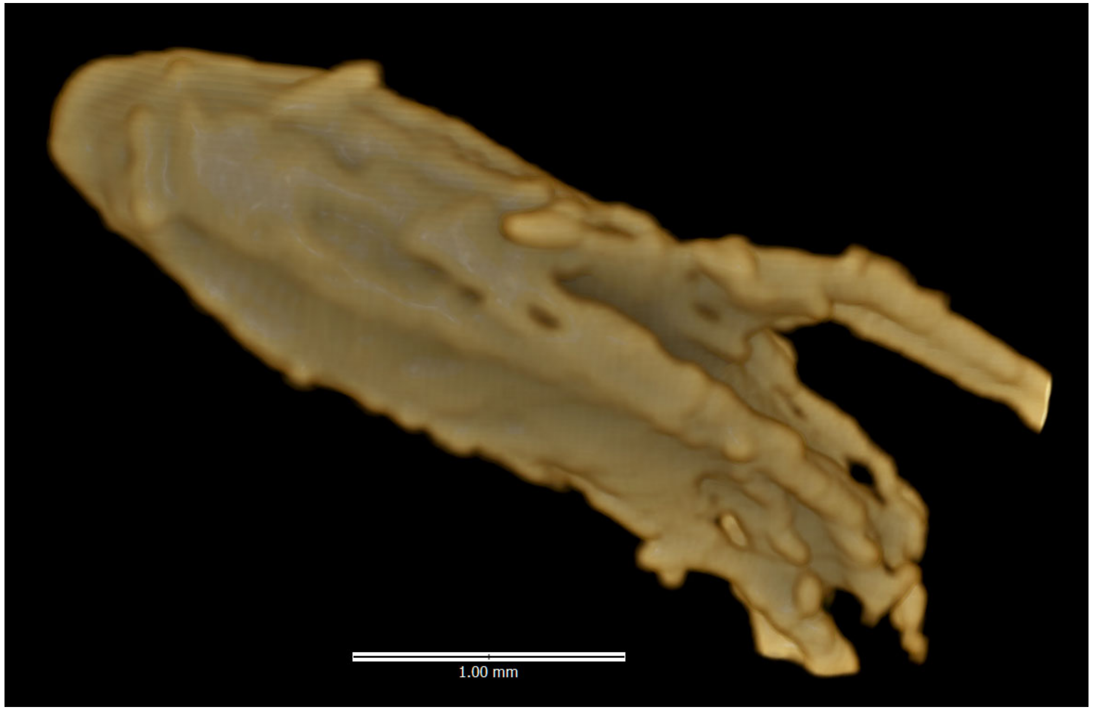

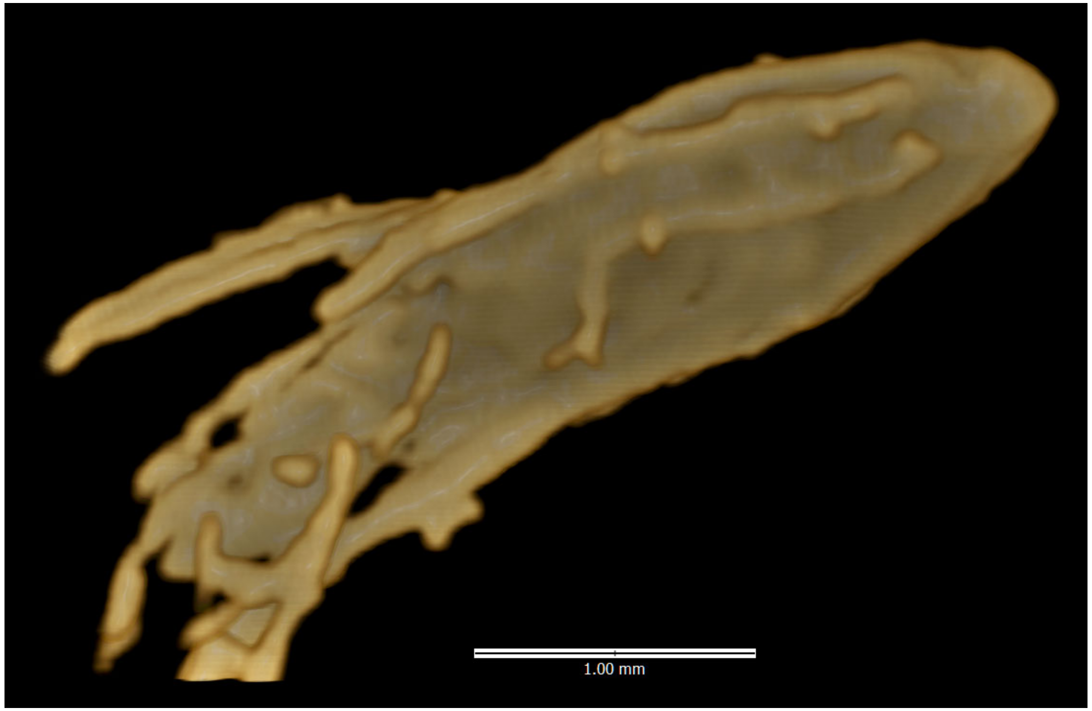

Inclusion 20. involucre, indeterminate attachment, solitary spikelet

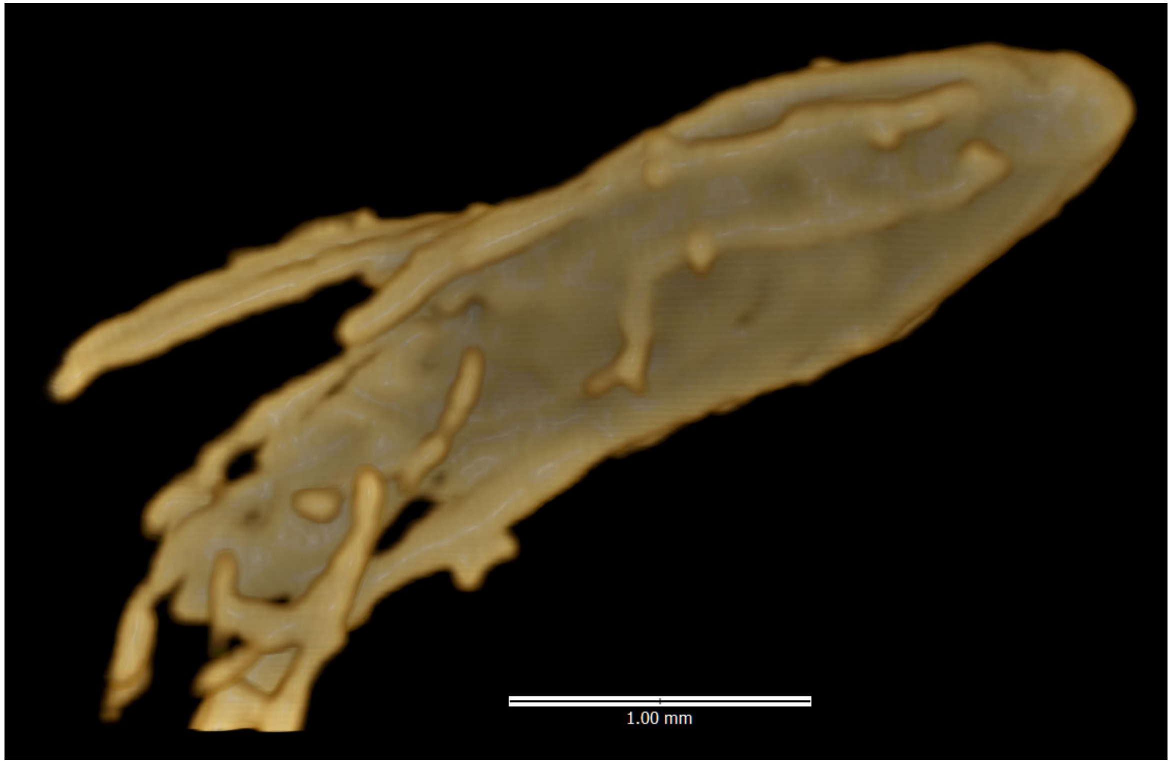

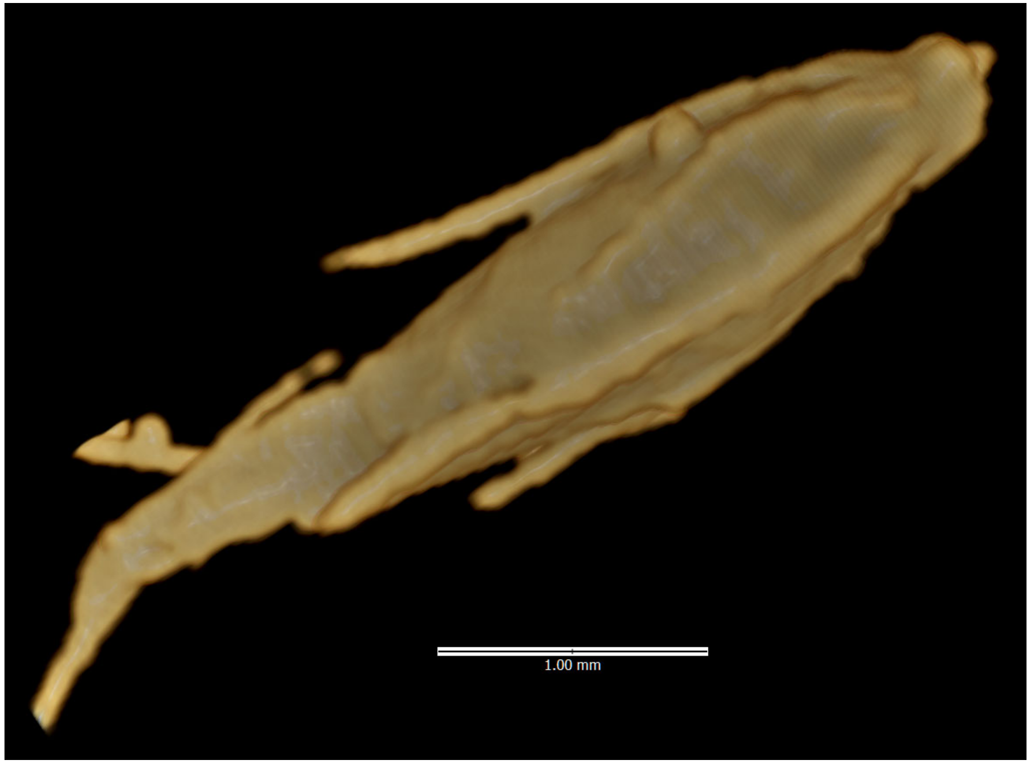

Inclusion 21. Two views: Sessile involucre(?), solitary spikelet. This specimen shows some elongation that could indicate formation of a peduncle, but it within the range of likely variation in sessile specimens with some additional torn culm tissue (i.e. if harvested slightly green).

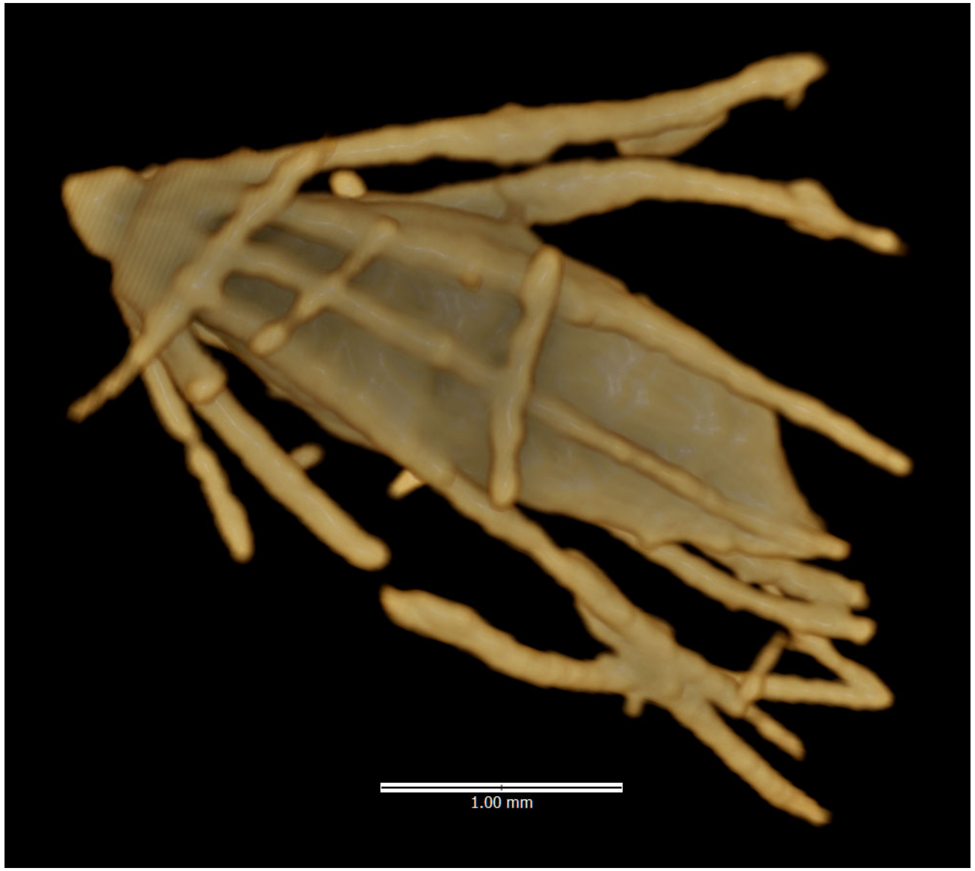

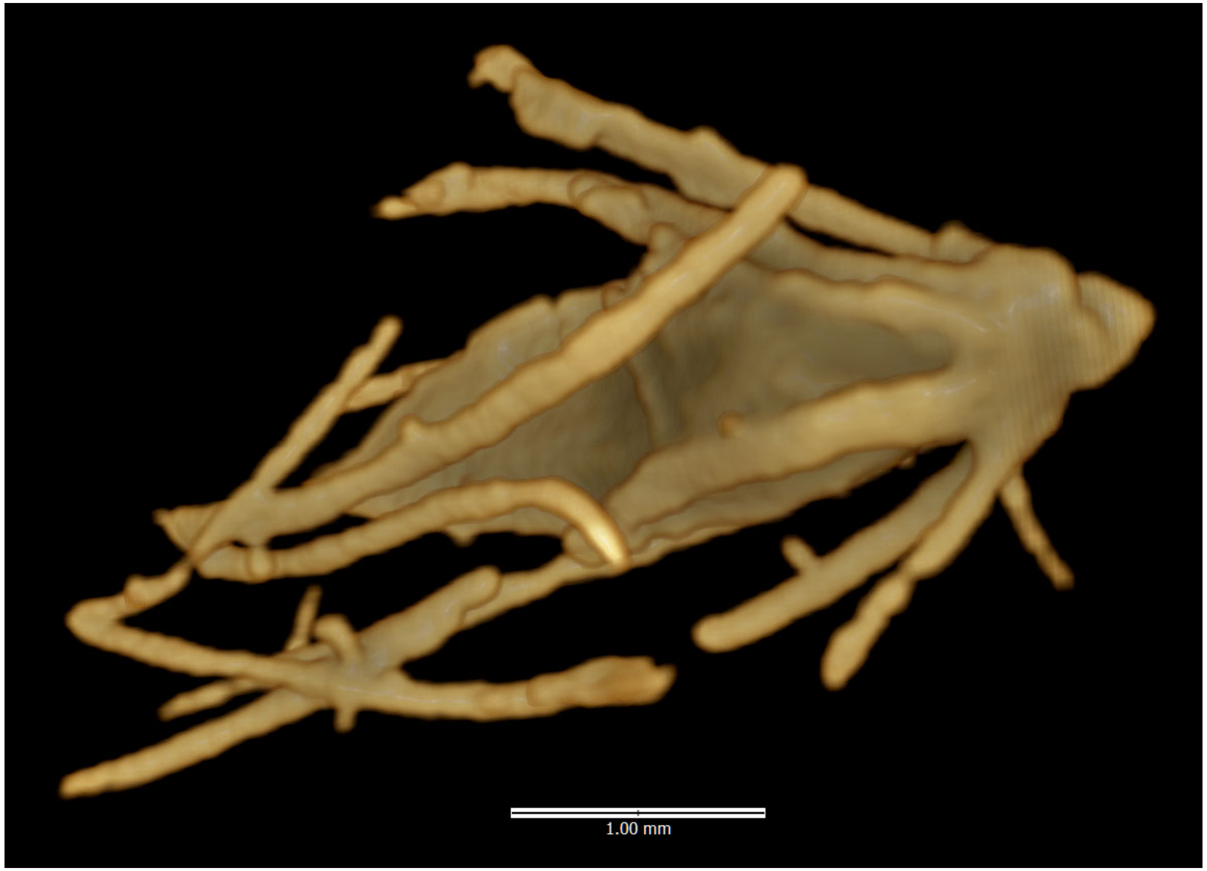

Inclusion 22. Two views: Sessile involucre, solitary spikelet

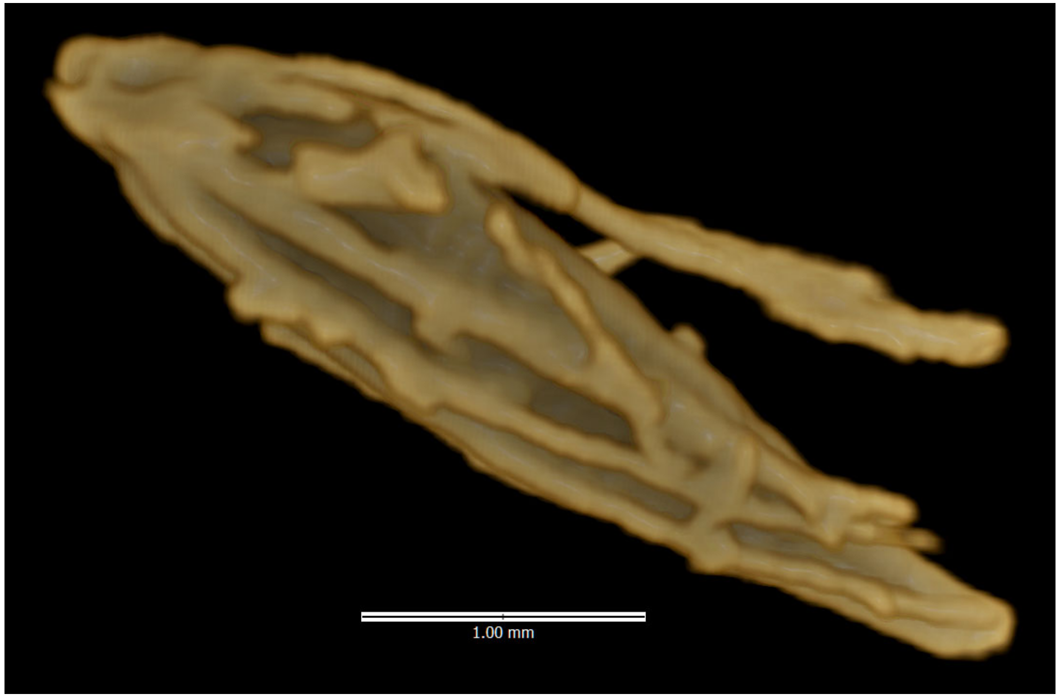

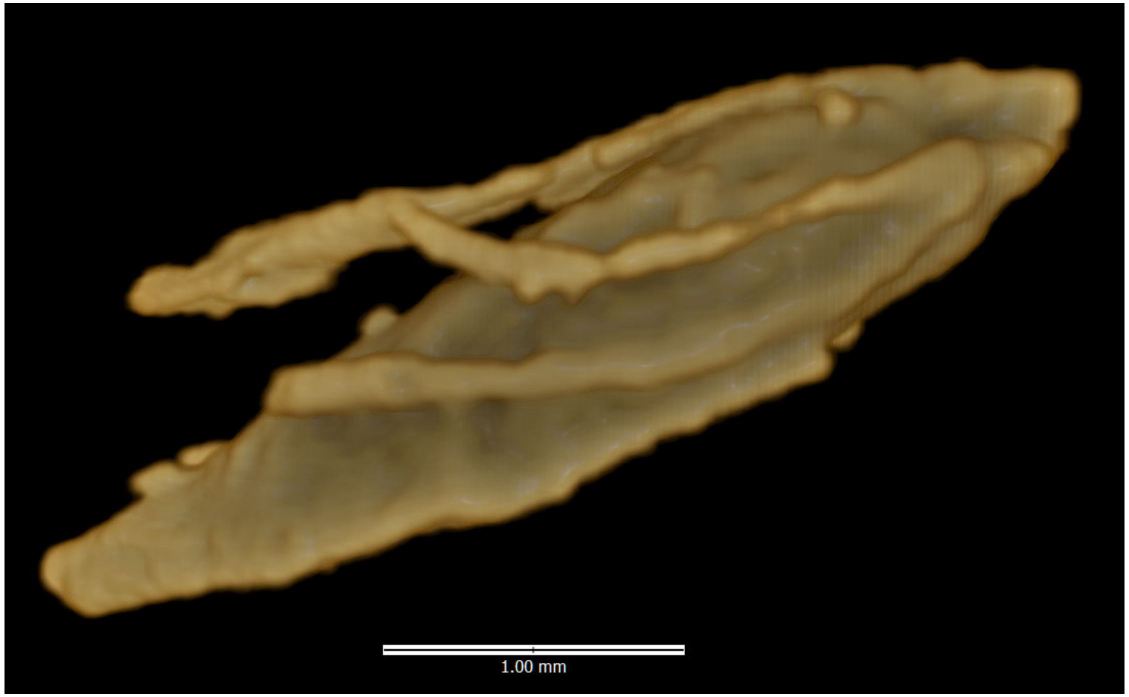

Inclusion 23. Two views: Sessile involucre, solitary spikelet

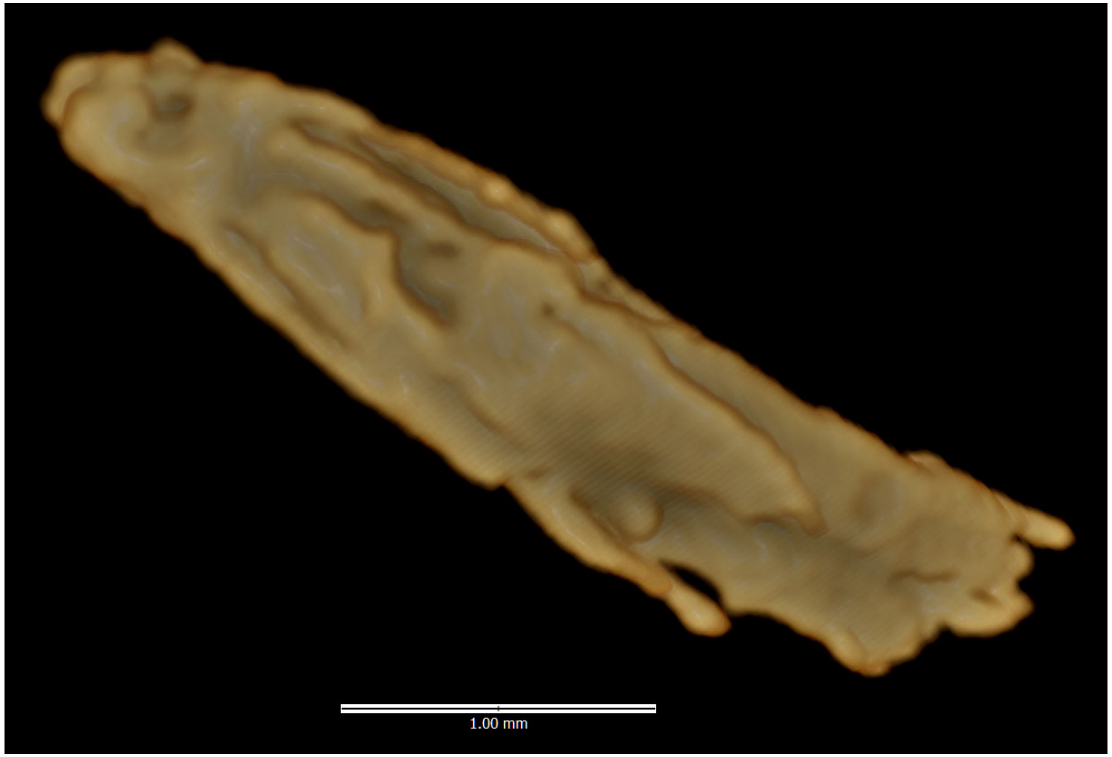

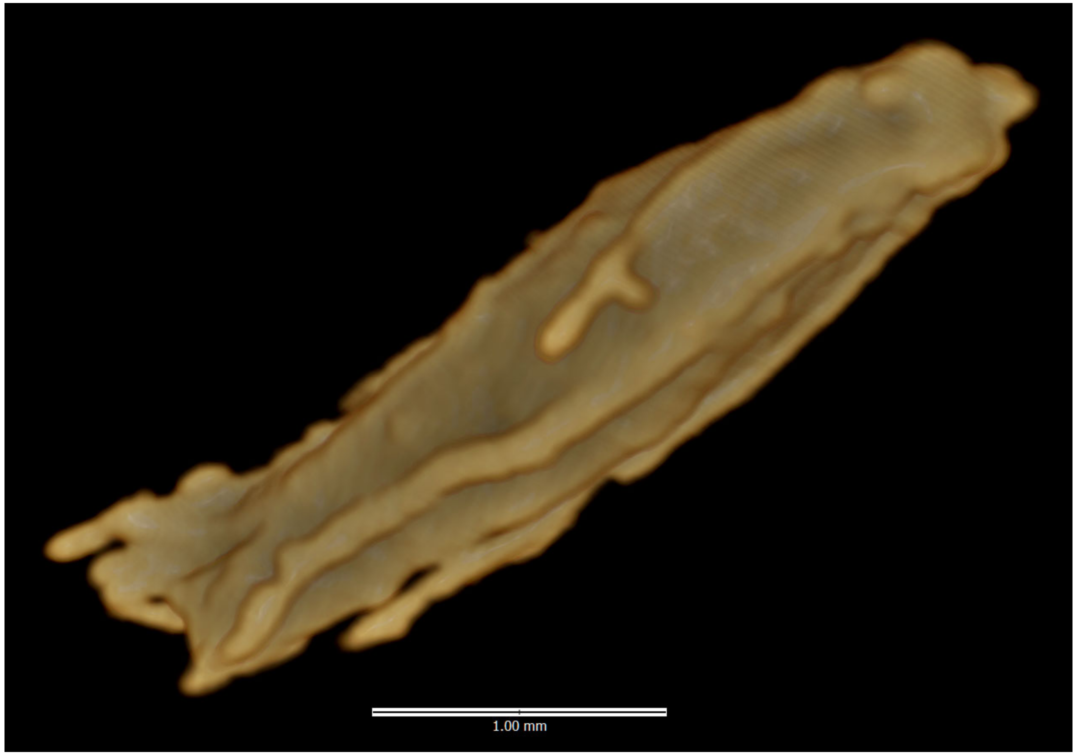

Inclusion 24. Two views: Sessile involucre, solitary spikelet

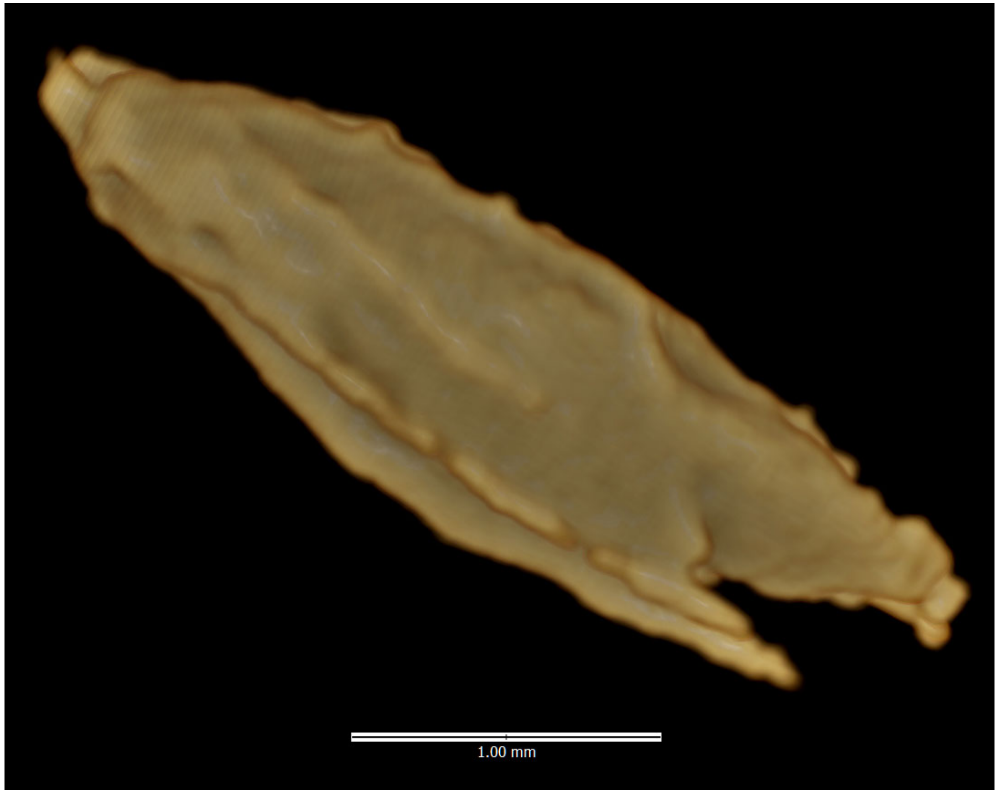

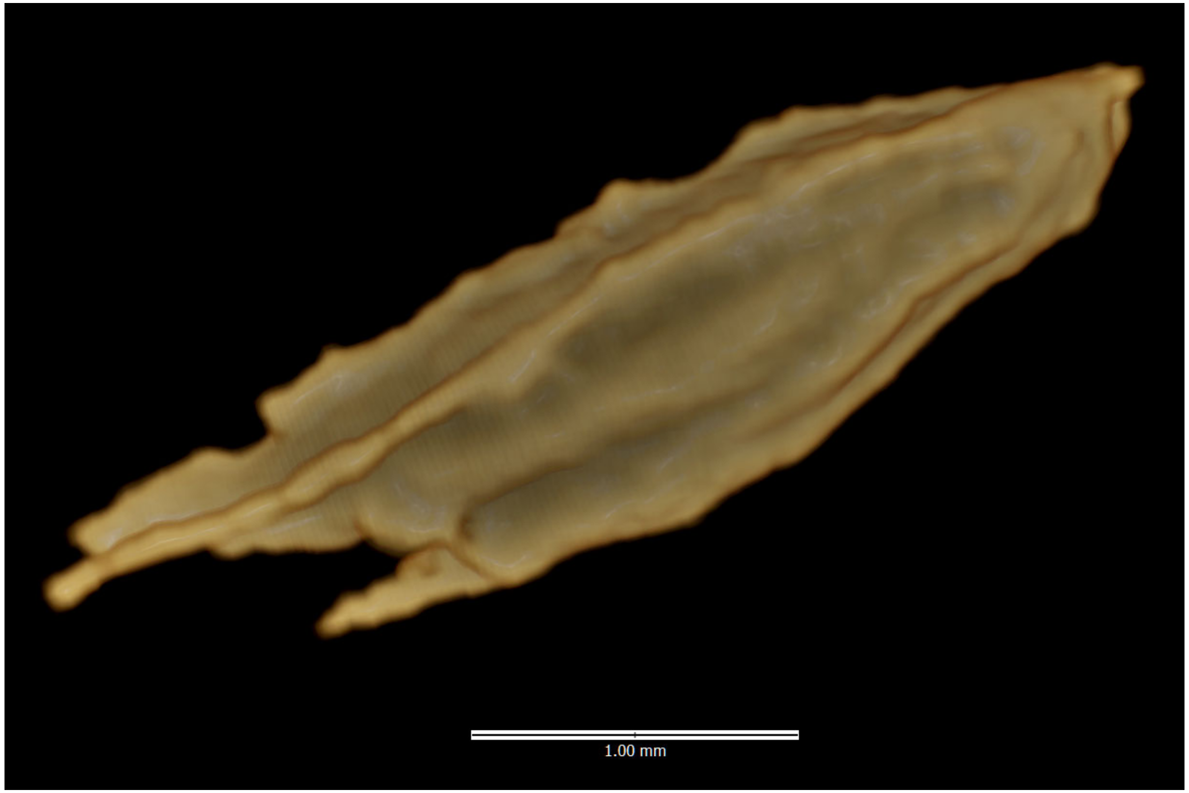

Inclusion 25

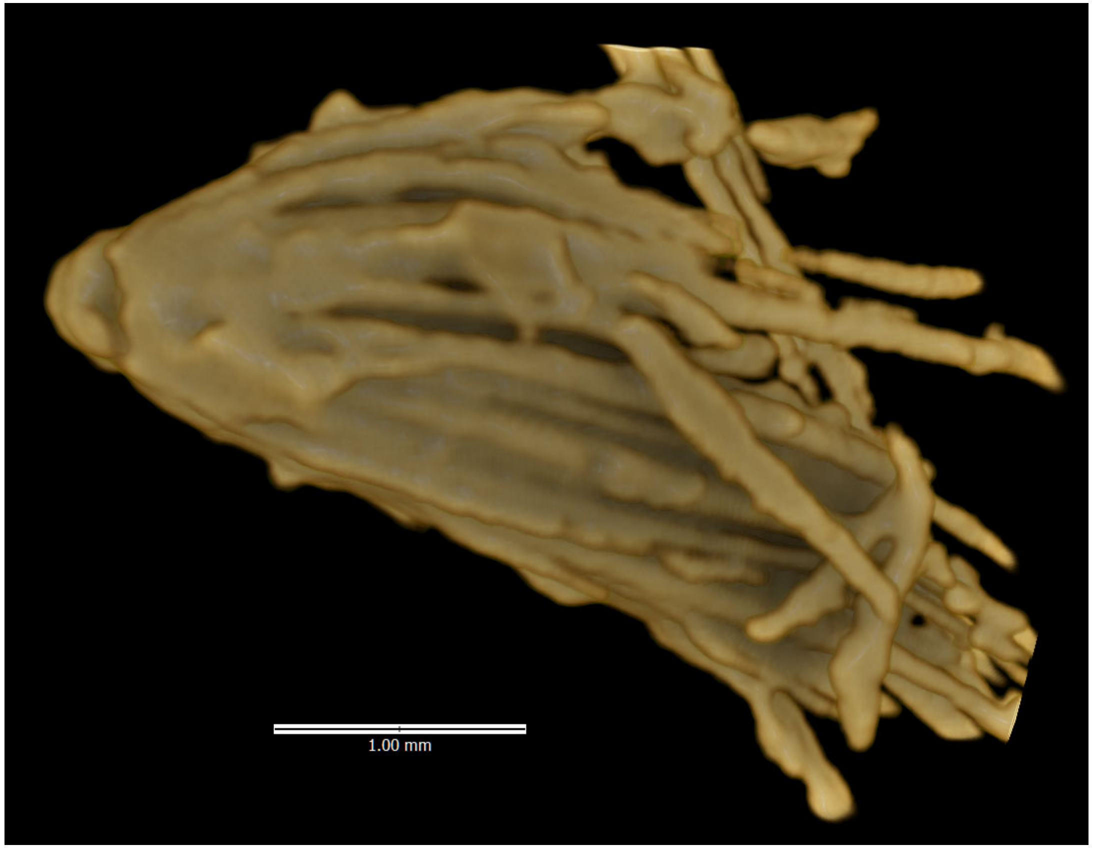

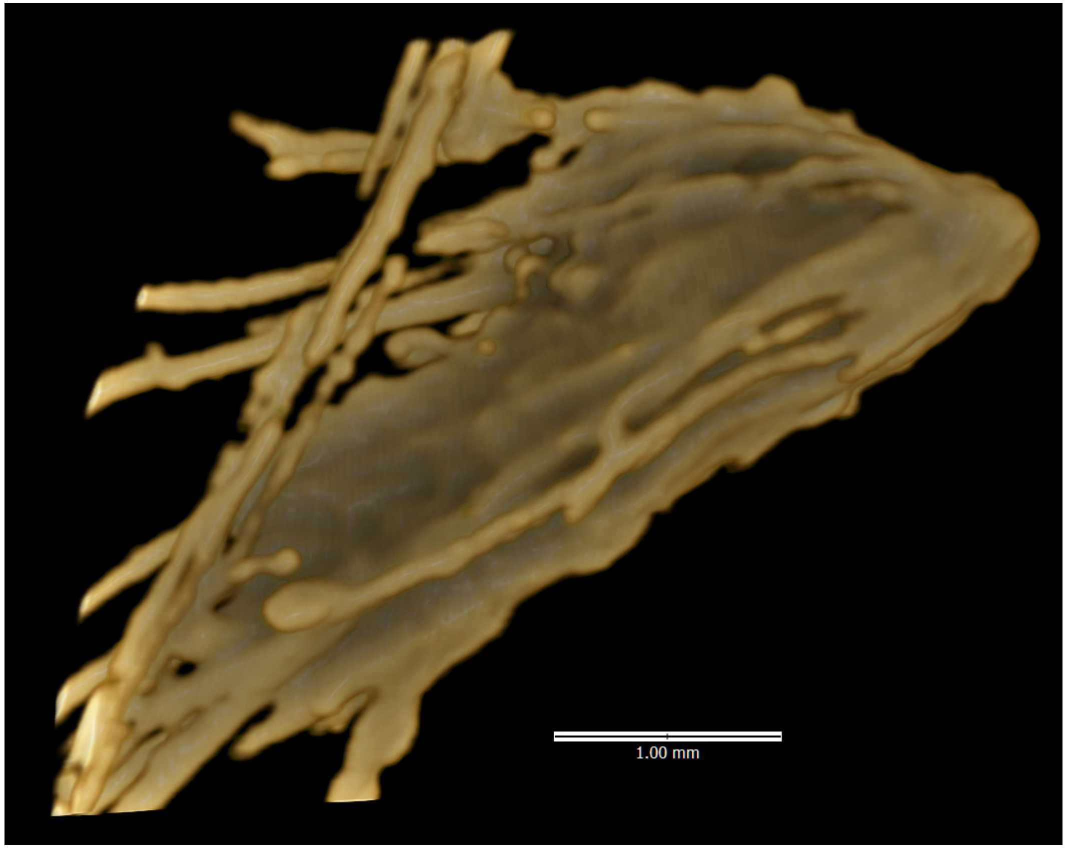

Inclusion 26. Two views: Sessile involucre, solitary spikelet

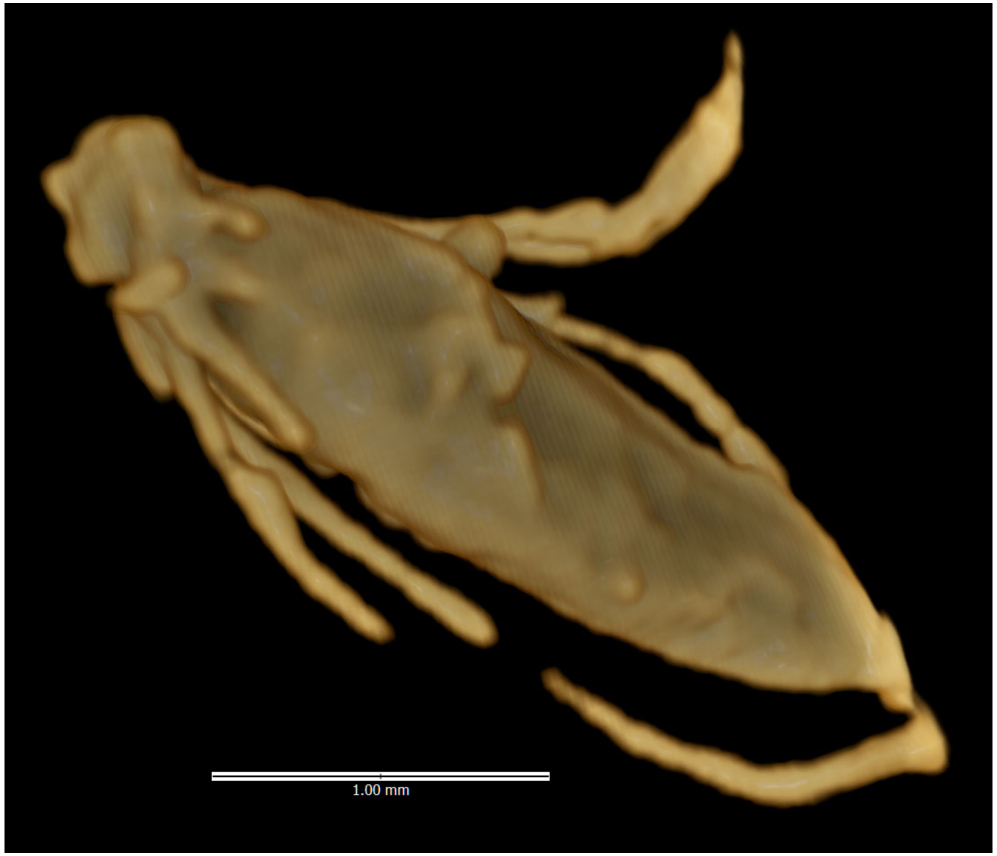

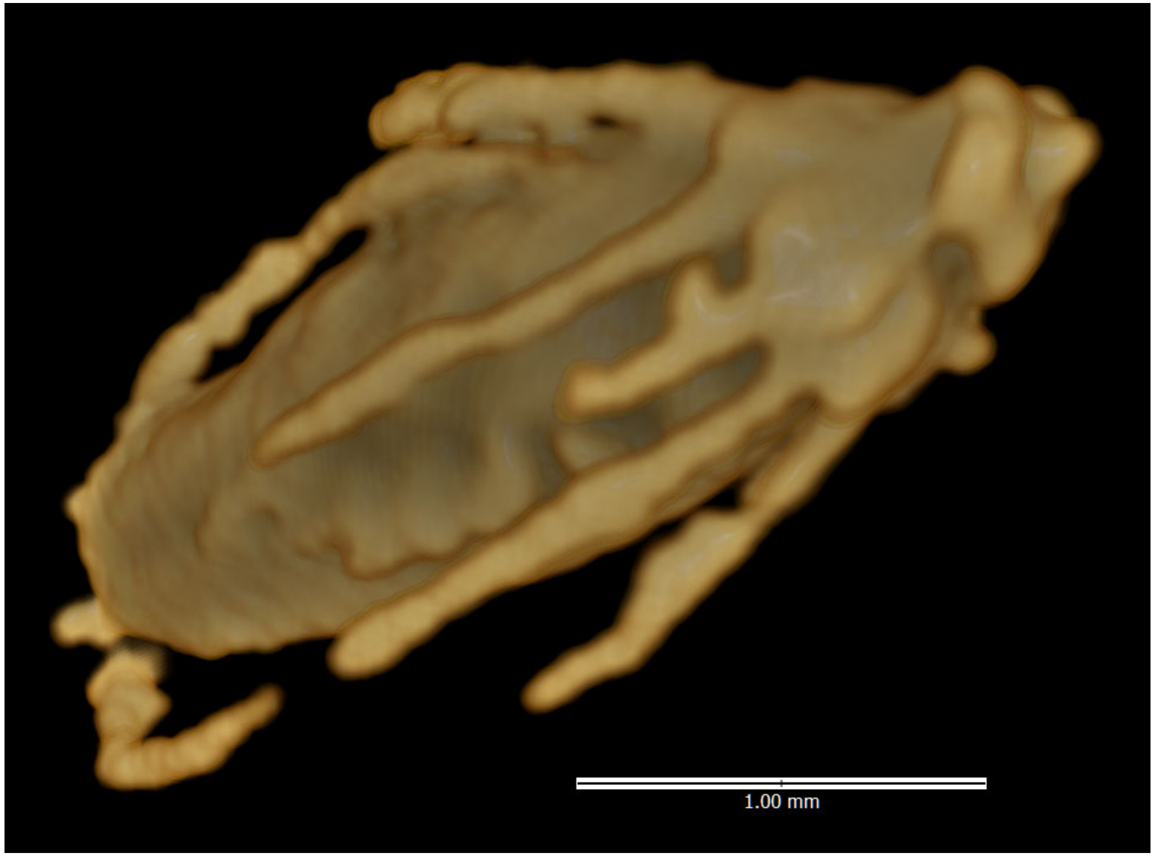

Inclusion 27. Two views: Sessile involucre, solitary spikelet

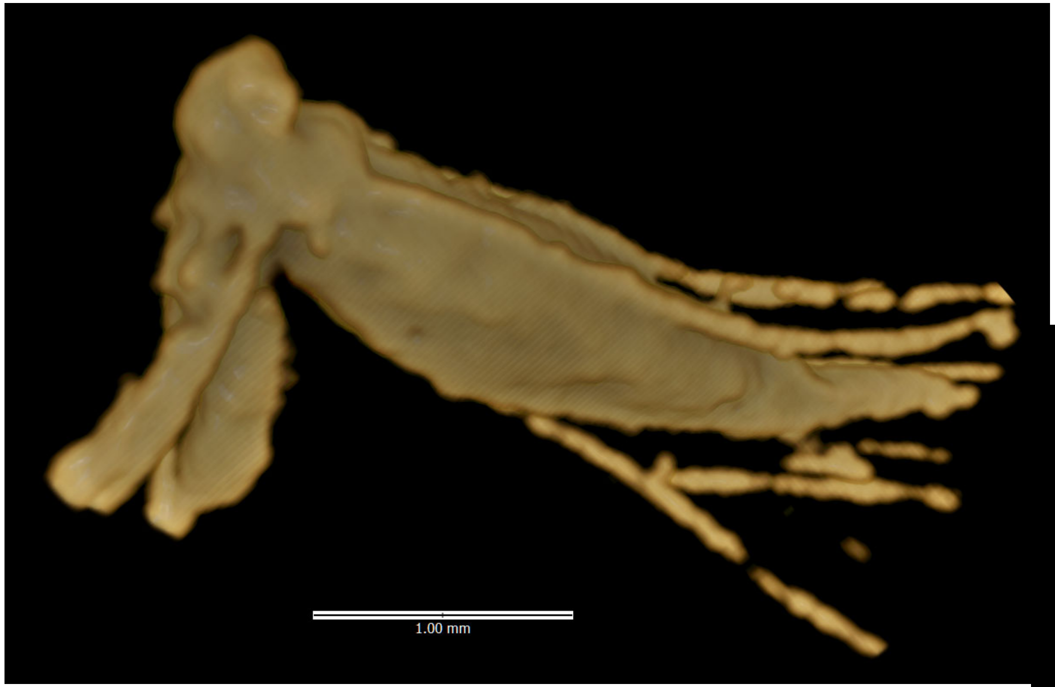

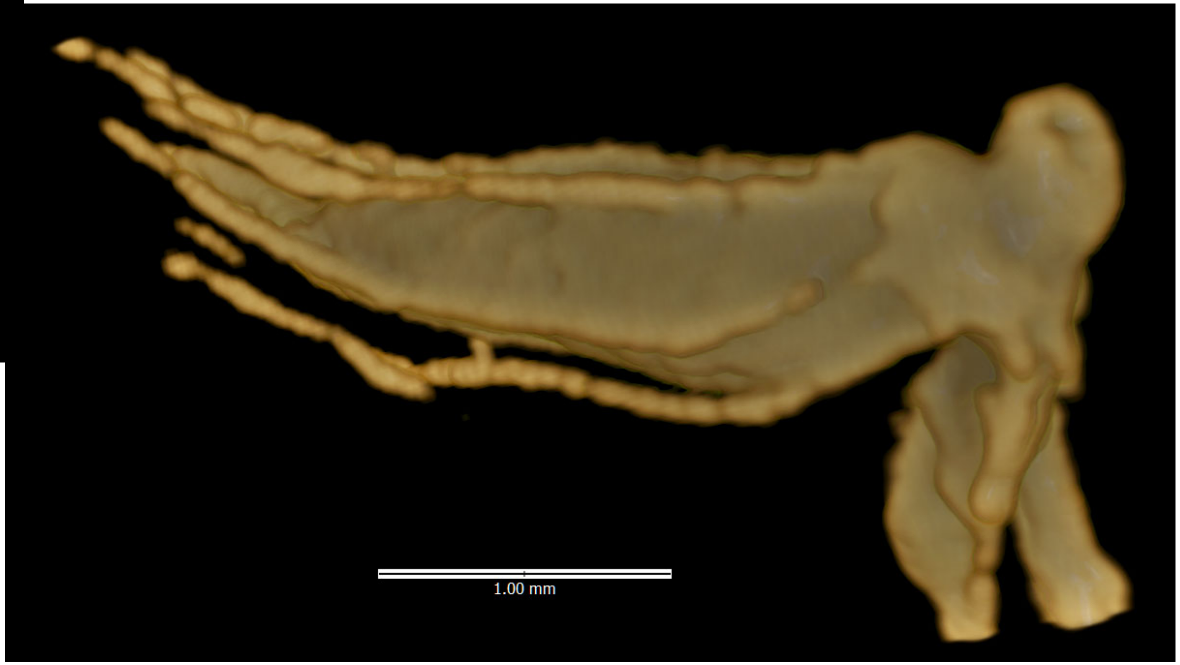

Inclusion 28. Two views: Sessile involucre, solitary spikelet

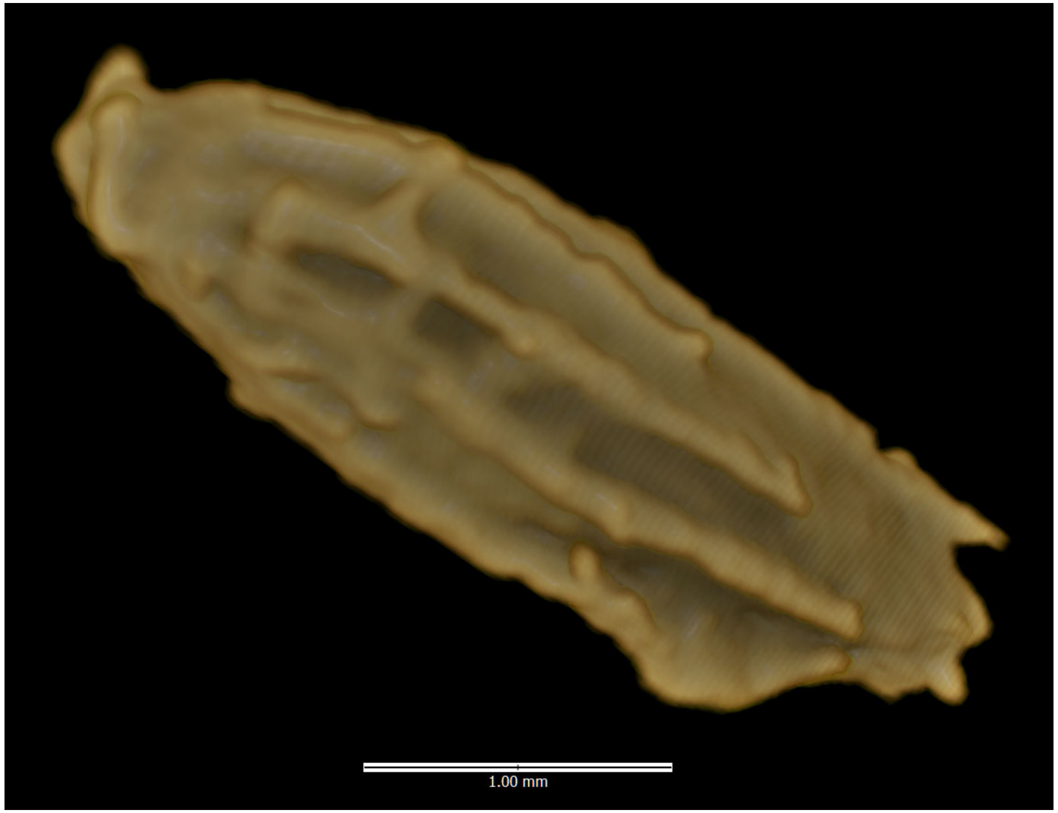

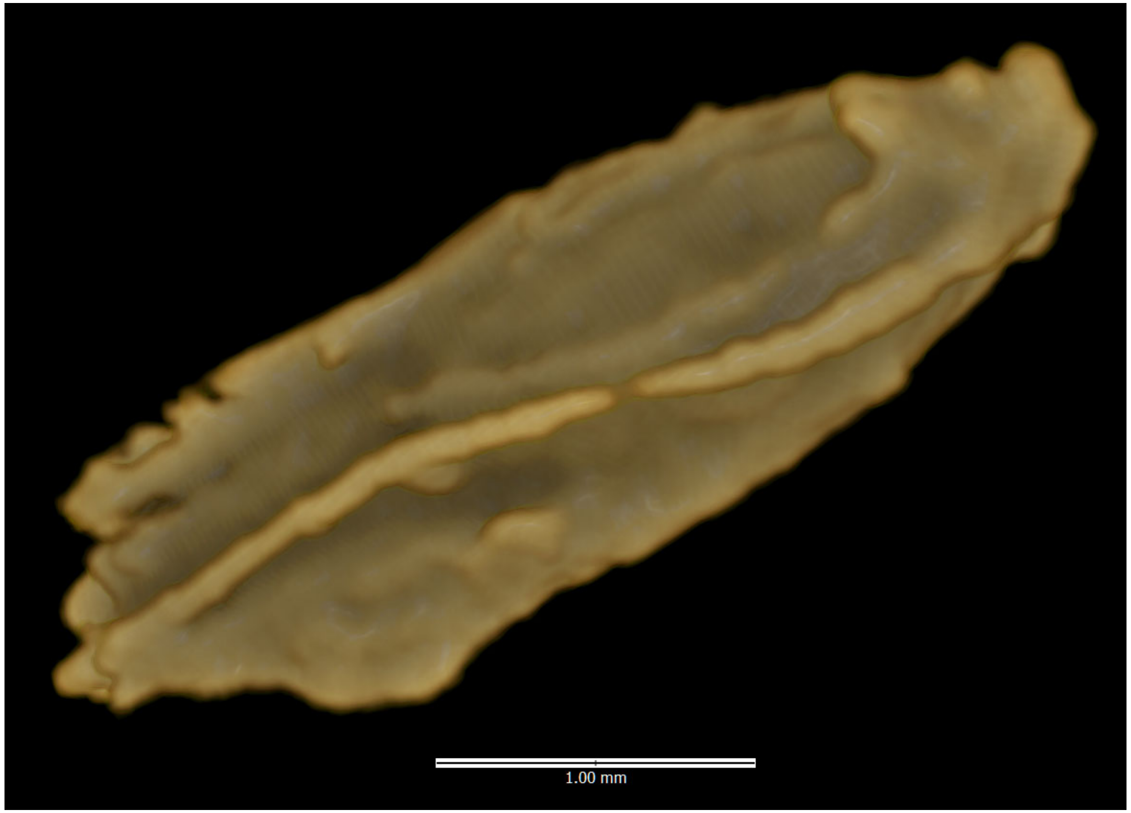

Inclusion 29. Two views: Sessile involucre, solitary spikelet

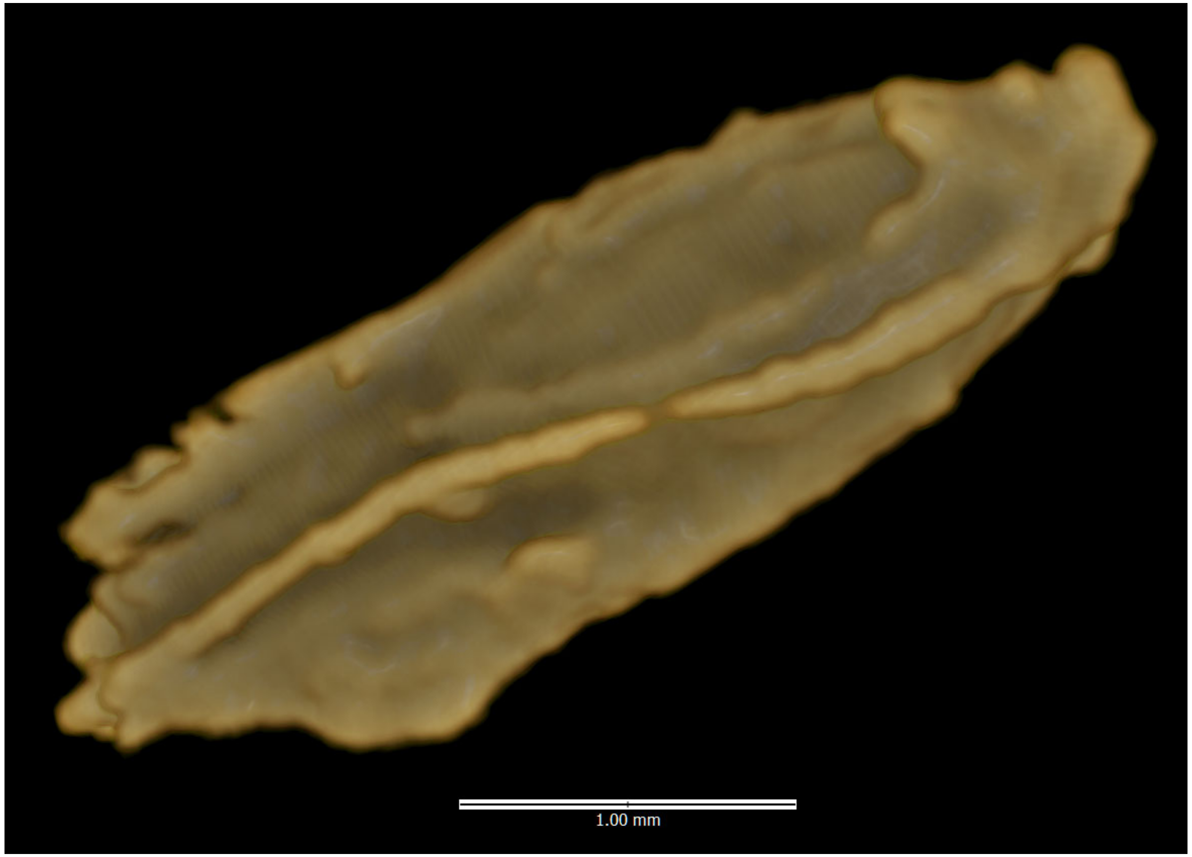

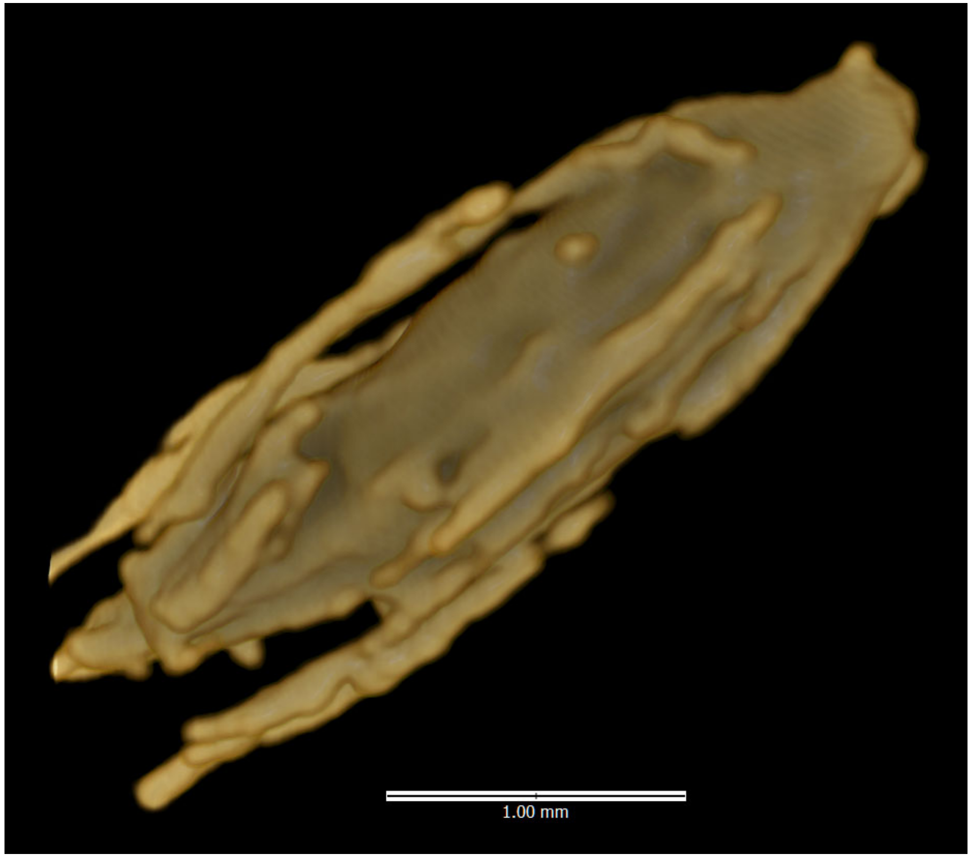

Inclusion 30. Two views: Sessile involucre, solitary spikelet

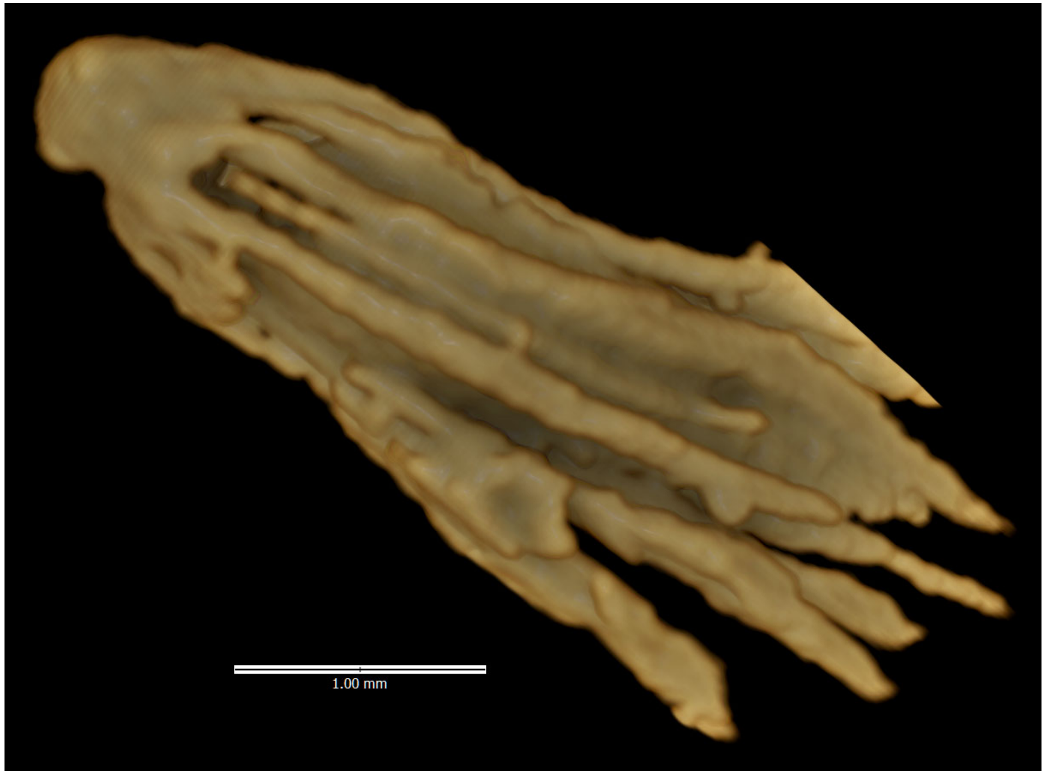

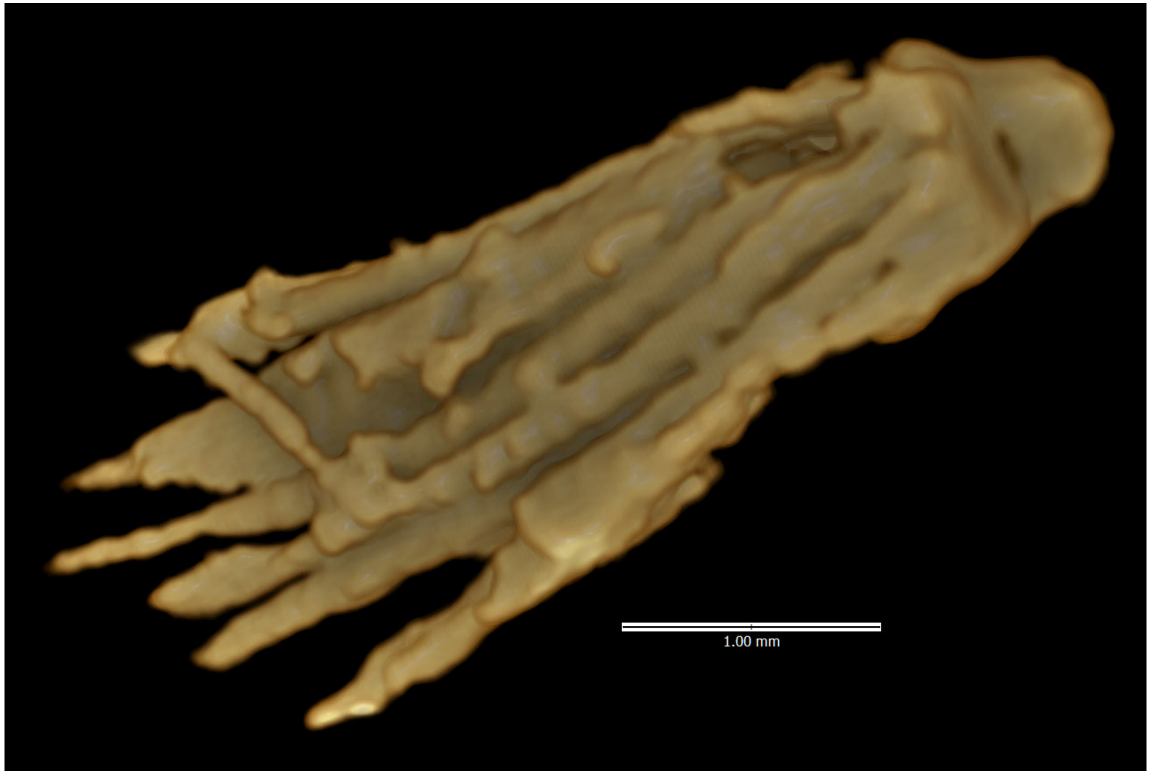

Inclusion 32. Two views: involucre, indeterminate attachment, solitary spikelet

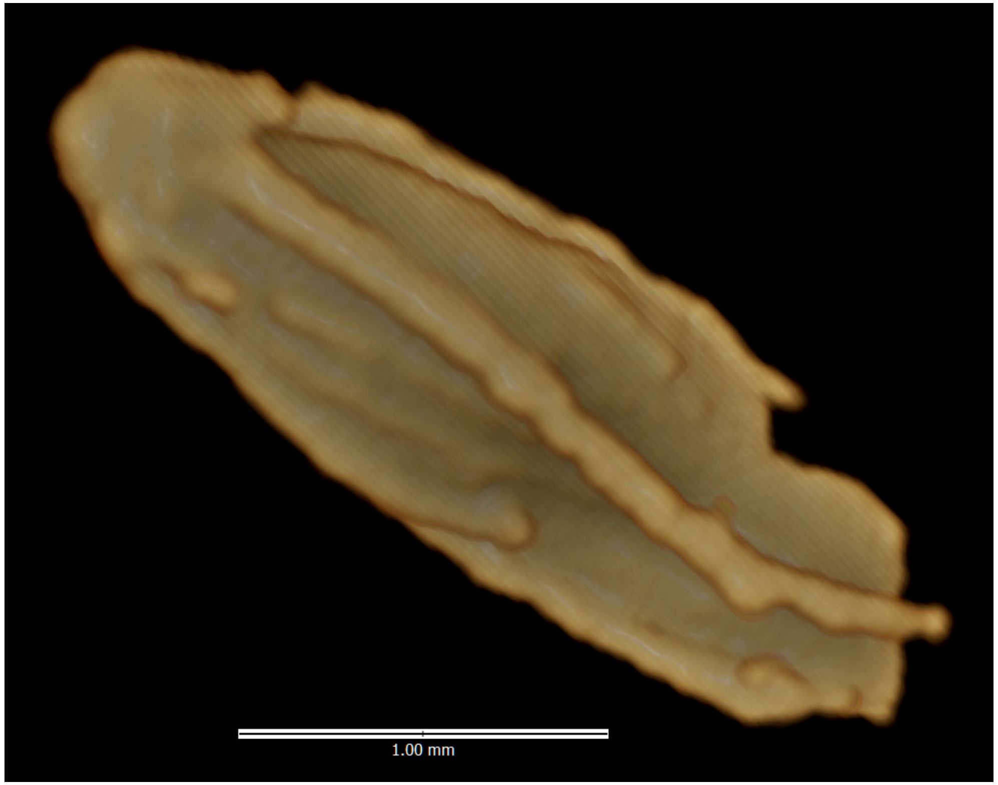

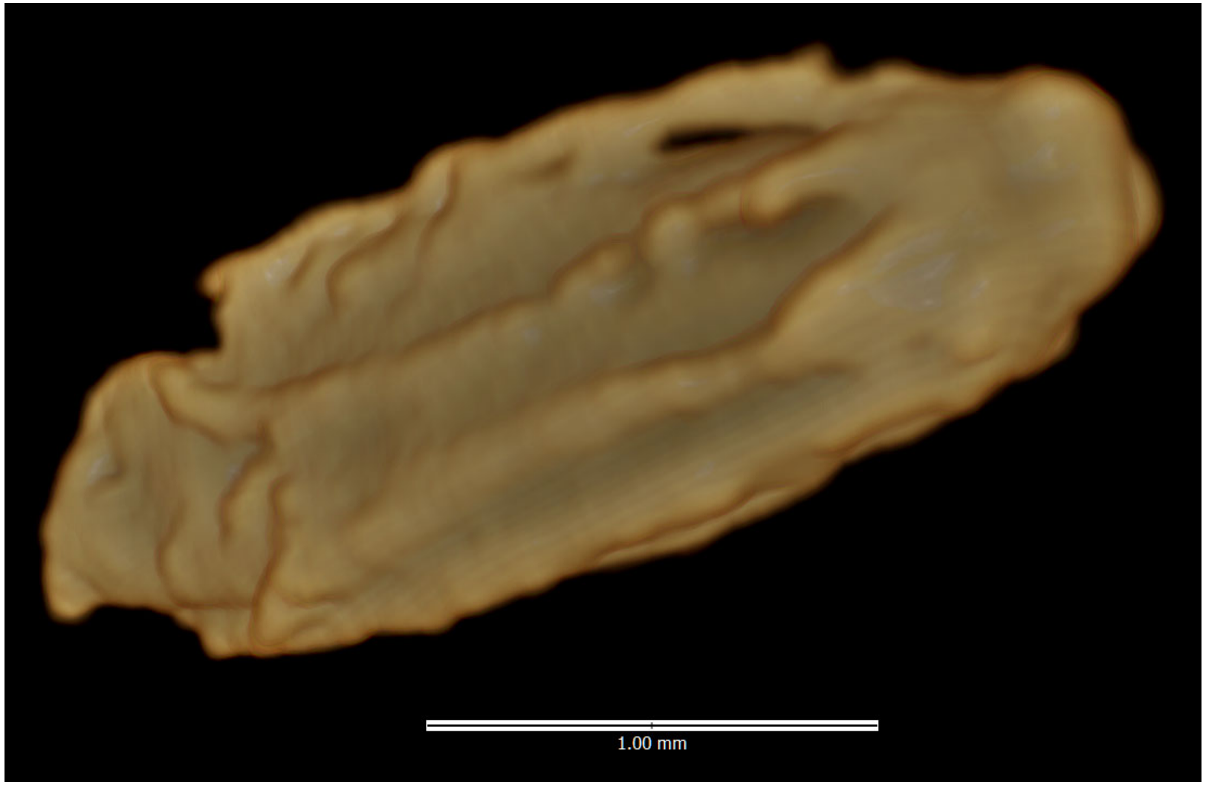

Inclusion 33. Two views: Sessile involucre, solitary spikelet

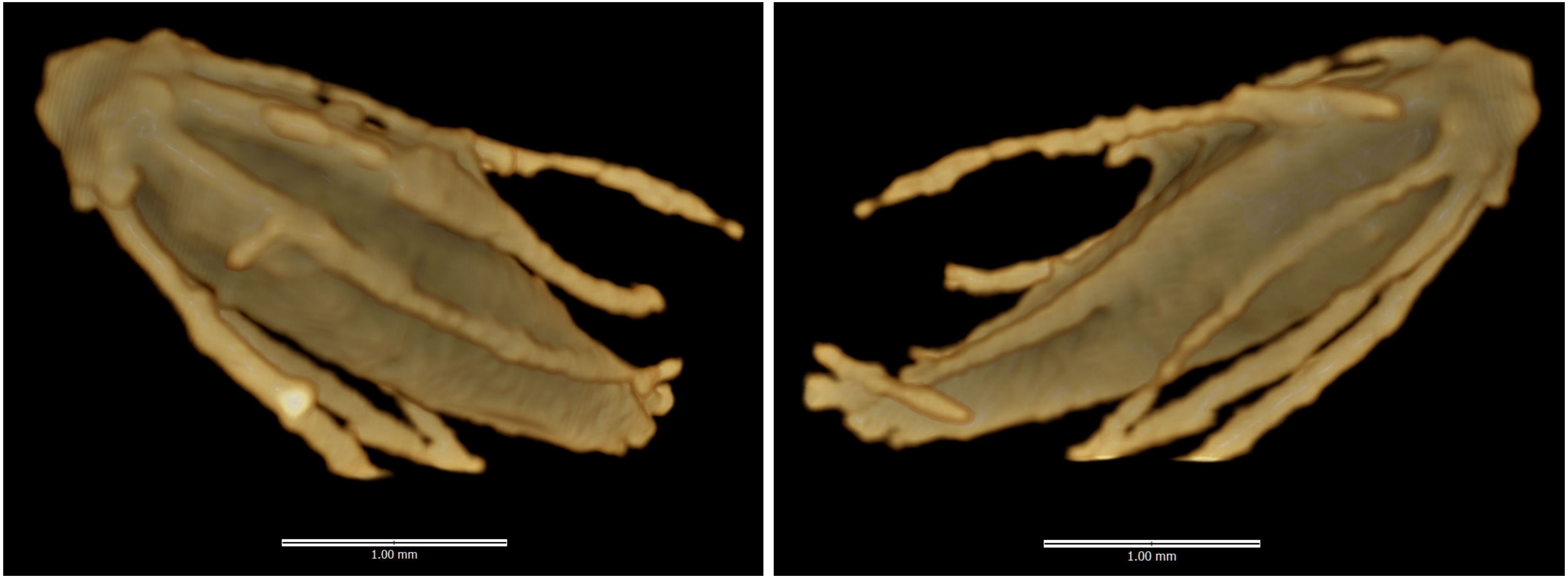

Inclusion 34. Two views: Sessile involucre, solitary spikelet

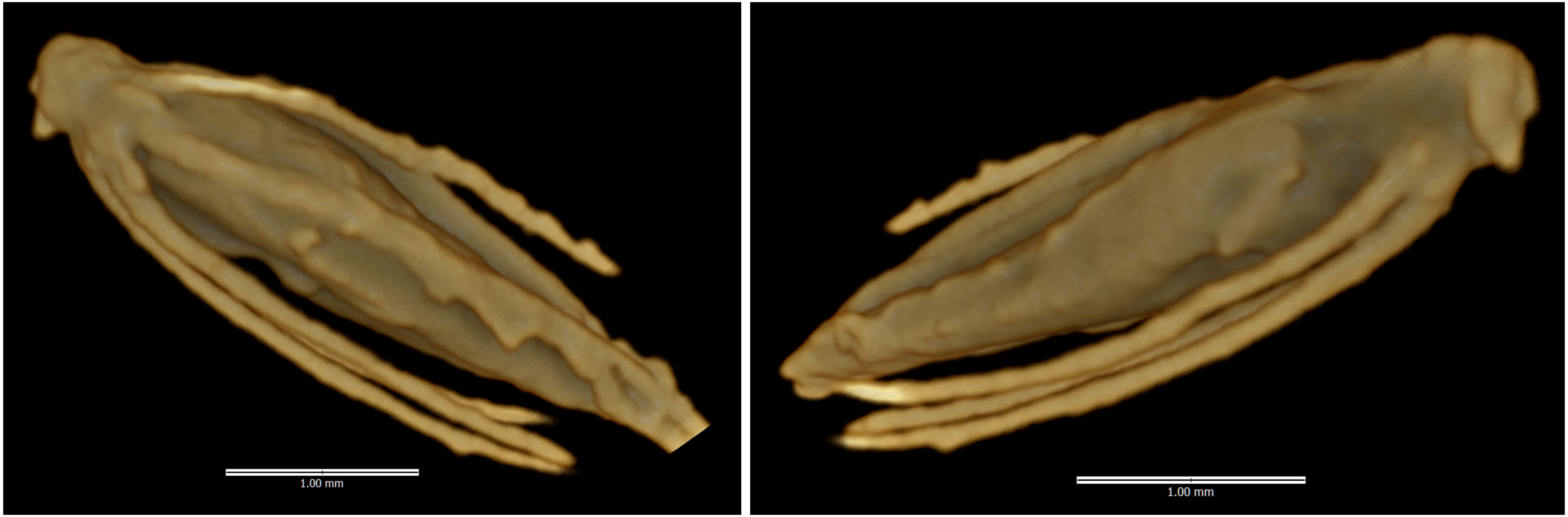

Inclusion 35. Two views: Sessile involucre, solitary spikelet

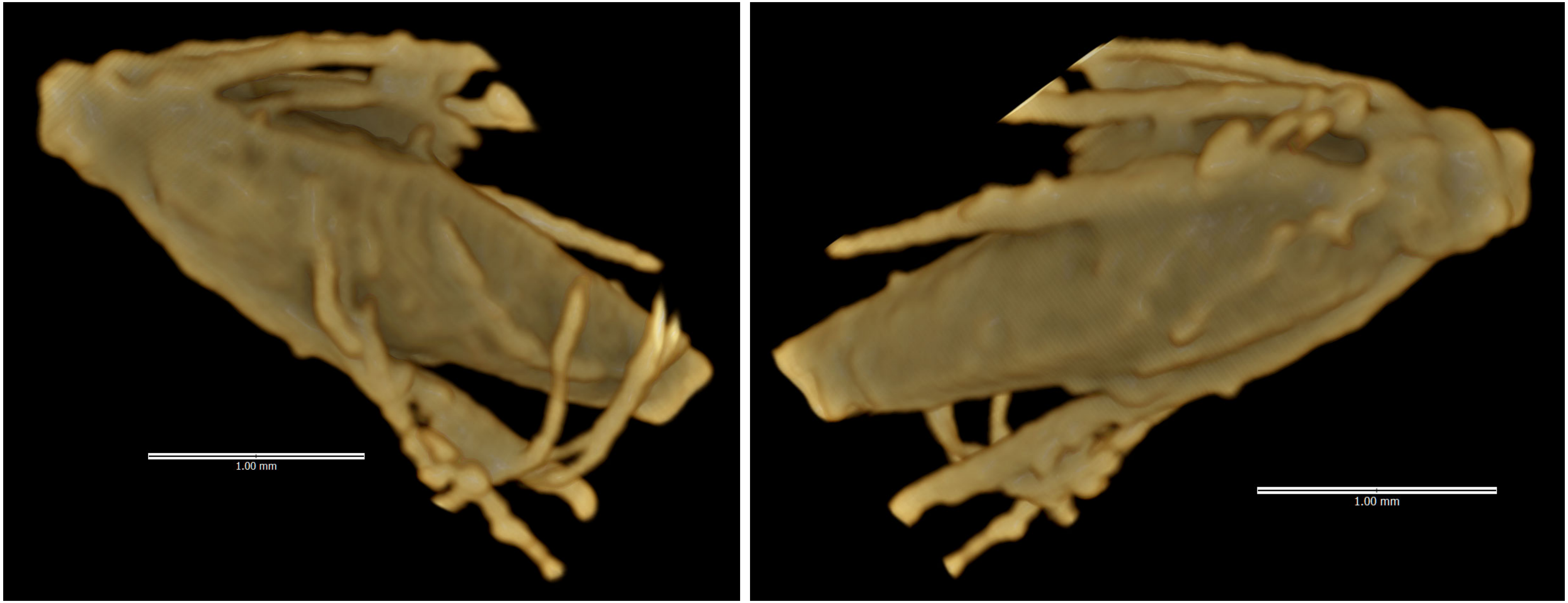

Inclusion 36. Two views: Sessile involucre, solitary spikelet

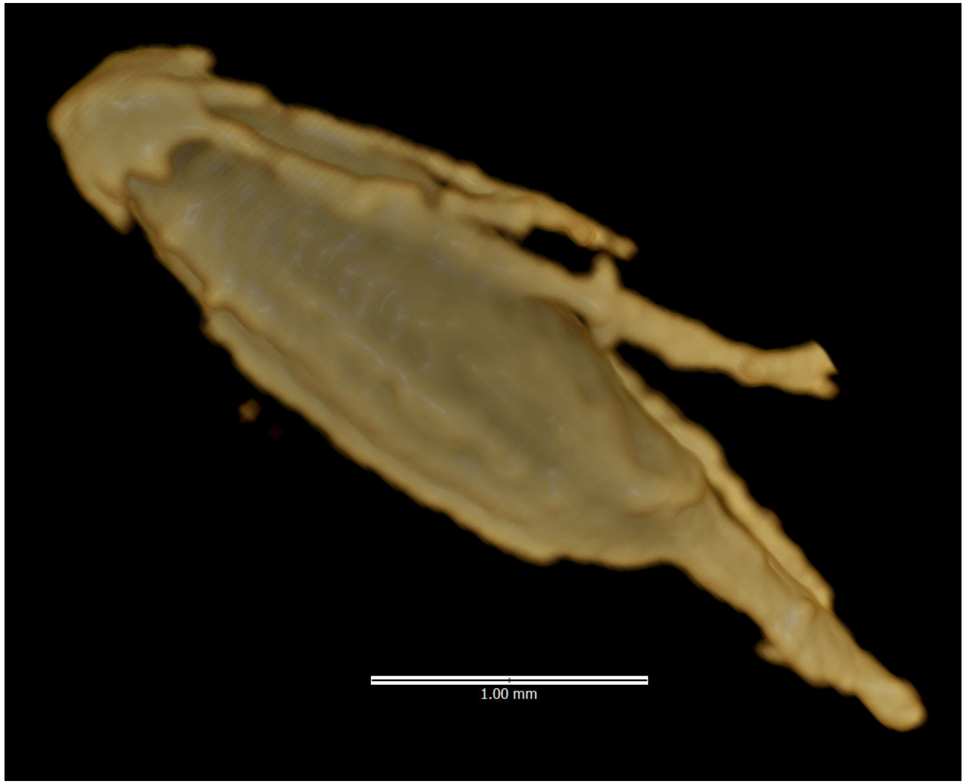

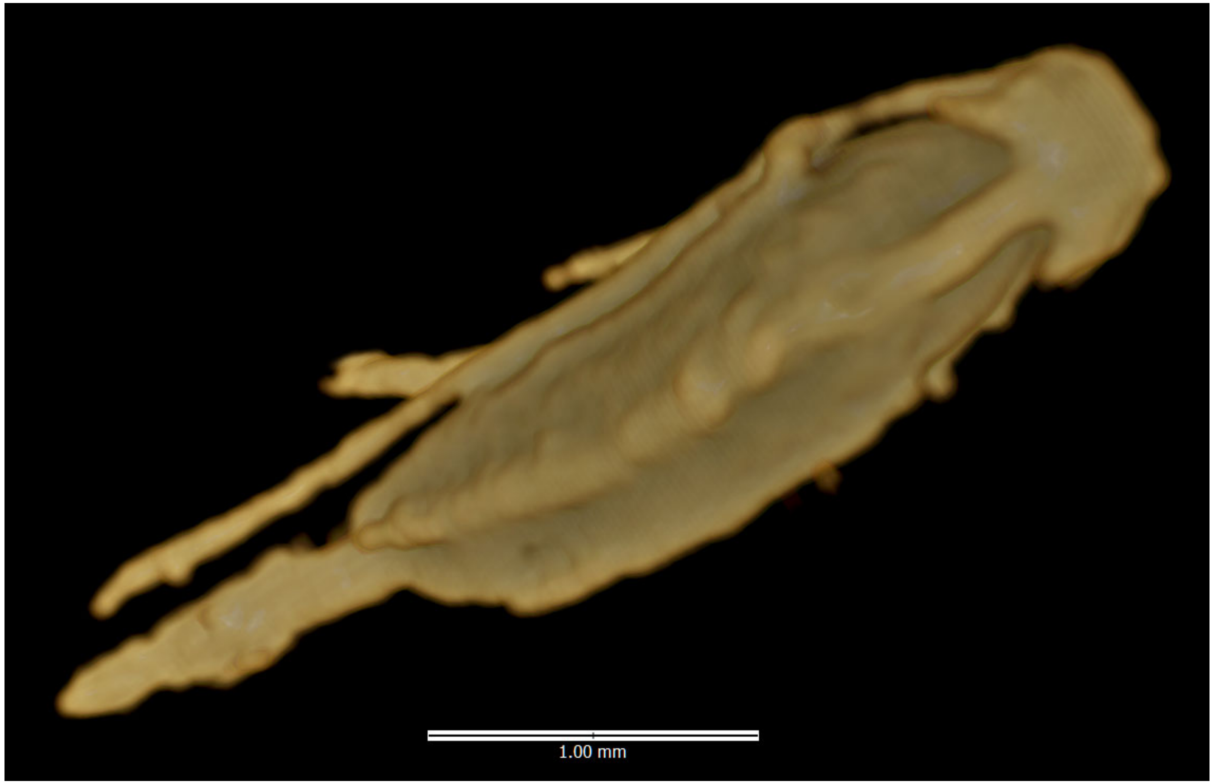

Supplement: Supplementary file 2 — (PDF 7241 kb) [file 10437_2021_9428_MOESM2_ESM.pdf]
